# Mechanochemical Studies on Coupling of Hydrazines and Hydrazine Amides with Phenolic and Furanyl Aldehydes—Hydrazones with Antileishmanial and Antibacterial Activities

**DOI:** 10.3390/molecules28135284

**Published:** 2023-07-07

**Authors:** Anna Kapusterynska, Christian Bijani, Damian Paliwoda, Laure Vendier, Valérie Bourdon, Nicolas Imbert, Sandrine Cojean, Philippe Marie Loiseau, Deborah Recchia, Viola Camilla Scoffone, Giulia Degiacomi, Abdul Akhir, Deepanshi Saxena, Sidharth Chopra, Vira Lubenets, Michel Baltas

**Affiliations:** 1 CNRS, LCC (Laboratoire de Chimie de Coordination), Université de Toulouse, UPS, INPT, Inserm ERL 1289, 205 Route de Narbonne, BP 44099, CEDEX 4, 31077 Toulouse, France; annakapusterynska@gmail.com (A.K.); christian.bijani@lcc-toulouse.fr (C.B.); damian.paliwoda@lcc-toulouse.fr (D.P.); laure.vendier@lcc-toulouse.fr (L.V.); 2 Technological and Expert Platform, Chemistry Institute of Toulouse ICT-UAR2599, University of Toulouse, CNRS, 118 Route de Narbonne, CEDEX 9, 31062 Toulouse, France; valerie.bourdon1@univ-tlse3.fr; 3 Antiparasite Chemotherapy, UMR 8076 CNRS BioCIS, Faculty of Pharmacy, University Paris-Saclay, 91400 Orsay, France; nicolas.imbert1@universite-paris-saclay.fr (N.I.); sandrine.cojean@universite-paris-saclay.fr (S.C.); philippe.loiseau@universite-paris-saclay.fr (P.M.L.); 4 Department of Biology and Biotechnology “Lazzaro Spallanzani”, University of Pavia, 27100 Pavia, Italy; deborah.recchia@unipv.it (D.R.); viola.scoffone@unipv.it (V.C.S.); giulia.degiacomi@unipv.it (G.D.); 5 Division of Microbiology, CSIR—Central Drug Research Institute, Sector 10, Janakipuram Extension, Sitapur Road, Lucknow 226031, Uttar Pradesh, India; abd.bstdli@live.com (A.A.); deepanshisaxena4@gmail.com (D.S.); skchopra.007@cdri.res.in (S.C.); 6 Department of Biologically Active Substances, Pharmacy and Biotechnology, Lviv Polytechnic National University, S. Bandery, 12, 79013 Lviv, Ukraine; vlubenets@gmail.com

**Keywords:** mechanochemistry, sustainable synthesis, hydrazones, furanyls, phenolics, anti-infectious, antileishmanial, antibacterial

## Abstract

Hydrazone compounds represent an important area of research that includes, among others, synthetic approaches and biological studies. A series of 17 hydrazones have been synthesized by mechanochemical means. The fragments chosen were phenolic and furanyl aldehydes coupled with 12 heterocyclic hydrazines or hydrazinamides. All compounds can be obtained quantitatively when operating on a planetary ball mill and a maximum reaction time of 180 min (6 cycles of 30 min each). Complete spectroscopic analyses of hydrazones revealed eight compounds (**3**–**5**, **8**–**11**, **16**) present in one geometric form, six compounds (**1**, **2**, **13**–**15**) present in two isomeric forms, and three compounds (**6**, **7**, **12**) where one rotation is restricted giving rise to two different forms. The single crystal X-ray structure of one of the hydrazones bearing the isoniazid fragment (**8**) indicates a crystal lattice consisting of two symmetry-independent molecules with different geometries. All compounds obtained were tested for anti-infectious and antibacterial activities. Four compounds (**1**, **3**, **5** and **8**) showed good activity against *Mycobacterium tuberculosis*, and one (**7**) was very potent against *Staphylococcus aureus*. Most interesting, this series of compounds displayed very promising antileishmanial activity. Among all, compound **9** exhibited an IC_50_ value of 0.3 µM on the *Leishmania donovani* intramacrophage amastigote in vitro model and a good selectivity index, better than miltefosine, making it worth evaluating in vivo.

## 1. Introduction

Hydrazone compounds represent an important area of research that includes synthetic approaches, theoretical and computational work [1,2,3], metal ion complexations [4,5,6], and biological studies [7,8,9]. Hydrazones also represent a class of important chemical building blocks with a broad range of biological activities. They can potentially exhibit properties such as antimicrobial [7], anti-inflammatory [8], antihypertensive [10], anti-infective [11], antimalarial [12], antileishmanial [13], anticancer [8], analgesic, and antioxidant [14]. Hydrazones, when suitably functionalized, can also form complexes possessing important biological properties. Hydrazones incorporating conjugated N, N, S (or O) tridentate systems can efficiently chelate transition metal ions such as iron, copper, or zinc to form metal complexes, thus interfering and acting against Fe-loading (such as α-thalassemia) [15], neurodegenerative diseases (Alzheimer) [4], or against cellular proliferation and DNA damage [16,17]. Finally, hydrazones can be transformed into hybrid azines an important class of compounds for antimicrobial therapy [18].

Hydrazones are formed by the reaction of hydrazine (or hydrazide) with aldehydes and ketones. They can also be synthesized by the Japp–Klingemann reaction from α-keto acids or α-keto esters and aryldiazonium salts. The first one, representing the most general method, has been reported in solution and under green conditions, i.e., ultrasound microwaves and mechanochemical ones. In a solution (ethanol, methanol, or butanol) under reflux (from 1 h to more than 12 h) and in the presence of an acid catalyst, hydrazones are obtained in fair to excellent yields, depending on the reactants used (30–90%). Recently, ultrasonic waves [19] were used to synthesize a series of hydrazones obtained in good to excellent yields and short reaction times. Hydrazones were also reported to be synthesized in high yields under solvent-free conditions using microwave irradiation [20].

In terms of mechanochemical syntheses of hydrazones, Hajipur et al. [21] reported for the first time the synthesis of hydrazone derivatives in quantitative yields by milling in a mortar with sodium hydroxide and silica gel as solid support. Kaupp et al. [22] obtained benzoyl hydrazones by milling stoichiometric amounts of benzhydrazine and solid aldehyde. Nun et al. [23] carried out the synthesis of hydrazones in quantitative yields by milling a large variety of protected hydrazines with equimolar amounts of carbonyl compounds. Colacino et al. [24] also reported a comparative mechanochemical study using various milling devices and jar materials for synthesizing the active pharmaceuticals, i.e., nitrofurantoin and dantrolene, as well as other hydrazone compounds bearing the hydantoin scaffold. The authors conducted the reactions in a planetary ball mill or in a SPEX mill afforded after 15 min–2 h of milling hydrazones in very good to excellent yields (87–98%).

Several years ago, we launched a research program focused on the mechanochemical synthesis of hydrazones and derivatives within potential biological activities. We first studied the reaction where the aldehyde partner has a phenol functionality. Using a P0 vibratory ball mill Pulverisette with a single ball, we obtained, after an average time of 4 h and at room temperature, hydroxy phenylhydrazones in excellent yields [25] and possessing strong antioxidant properties. Later, using the same mechanochemical equipment as before (P0 vibratory ball mill), we reported on a series of isoniazid derivatives bearing phenolic, aromatic, or heteroaromatic fragments [26]. All compounds were obtained in very good to excellent yields (80–99%). Most of them presented a very potent activity against *M. tuberculosis* [26]. More recently, we reported the synthesis of hydrazones obtained from hydralazine hydrochloride coupled with various aldehydes. The hydrazones obtained are valuable intermediates (via two steps or one-pot two steps) in the elaboration by mechanochemical means of 1,2,4-triazoles with potent activity against *M. tuberculosis.* The synthesis of hydrazones (63–98% yields) and triazoles (60–98%yields) proceeded in a planetary Micro Mill Pulverisette 7 (P7) premium line with two grinding stations in the presence of pyrogenic S13 silica and sodium acetate [27].

**Figure 1:**
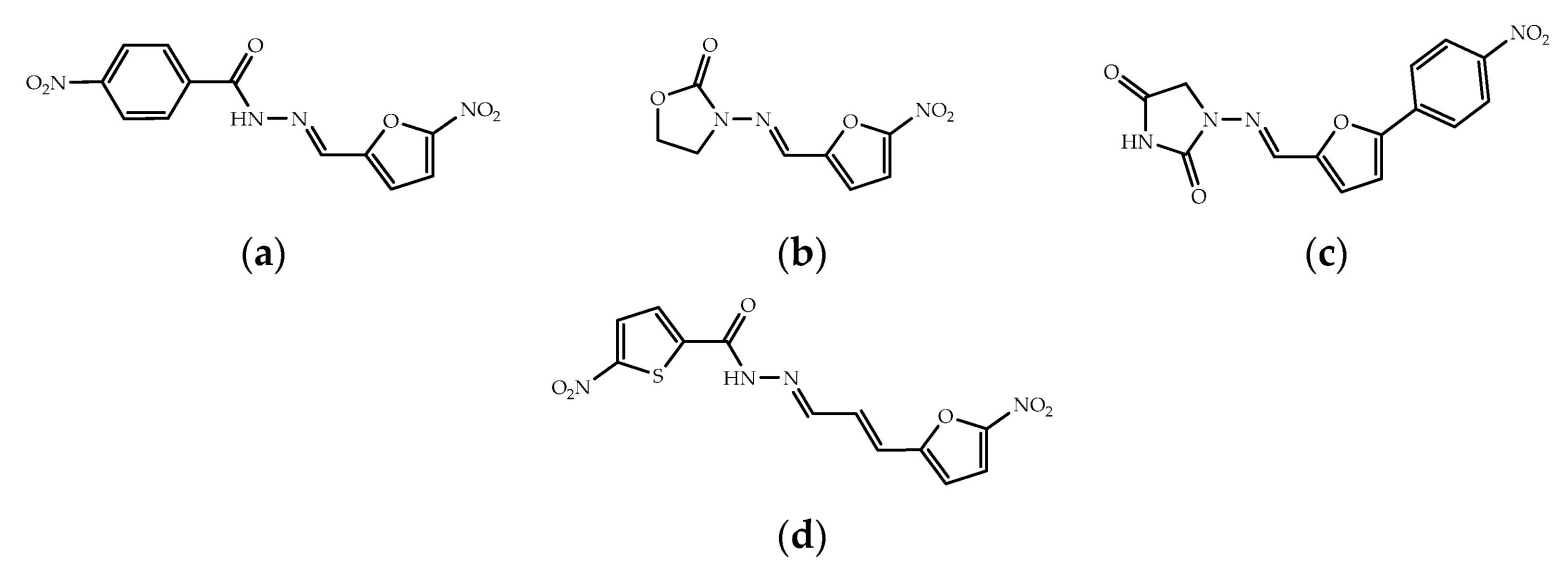
Chemical structures of active pharmaceuticals containing hydrazine-hydrazone moiety and a furanyl scaffold: (**a**) nifroxazide; (**b**) furazolidone; (**c**) dantrolene; (**d**) nifurzide.

In continuation to our work, we wish to report our findings in the construction by mechanochemical means of a series of hydrazones constructed by coupling a variety of 12 hydrazines and hydrazinamides with vanillin and furanyl aldehydes. Concerning the furanyl derivatives various hydrazones bearing the 5-nitrofuranyl-2-yl are already known as active pharmaceutical ingredients (API) like the anti-infective nifroxazide (Figure 1a) and the antibacterial and antiprotozoal furazolidone (Figure 1b). Our choice was focused on 5-nitrofuranyl-2-acryl and 5-(p-nitrophenyl) furanyl-2-yl derivatives as in the case of the muscle relaxant dantrolene (Figure 1c) and the intestinal anti-infective nifurzide (Figure 1d). In addition, intense research is conducted on these derivatives in relation to their biological activities [28,29,30].

Thus, 17 new compounds have been synthesized and evaluated in vitro against the parasite *L. donovani* and seven bacteria: *M. tuberculosis*, *S. aureus*, *Escherichia coli*, *Klebsiella pneumoniae*, *Acinetobacter baumannii*, *Pseudomonas aeruginosa,* and *Enterococcus* spp.

## 2. Results and Discussion

### 2.1. Hydrazones Bearing the Vanillin Frame

#### 2.1.1. Synthesis and Characterization

**Figure 2:**
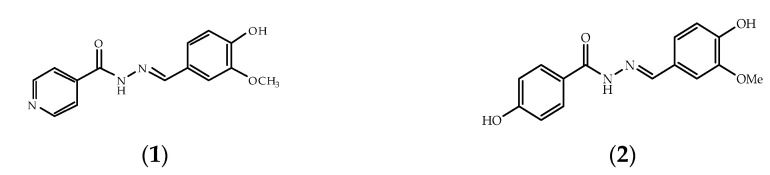
Chemical structures of synthesized hydrazones bearing the vanillin frame: (**1**) N′-[(E)-(4-Hydroxy-3-methoxyphenyl)methylene]isonicotinohydrazide; (**2**) 4-Hydroxy-N′-[(E)-(4-hydroxy-3-methoxyphenyl)methylidene]benzohydrazide.

We report first our experiments and results concerning the synthesis of hydrazones bearing the vanillin frame. This part is in direct continuation to our previous work; two compounds that have been synthesized and studied are the N′-[(E)-(4-Hydroxy-3-methoxyphenyl)methylene]isonicotinohydrazide (Ftivazide) **1** and the 4-Hydroxy-N′-[(E)-(4-hydroxy-3-methoxyphenyl)methylidene]benzohydrazide **2** (Figure 2).

Concerning the obtention of these two derivatives, we operated in two different mills: the Mixer Mill MM400 (Retsch) and the P7 premium line with two grinding stations (Fritsch). When reacting vanillin with isoniazid in a vibratory ball-mill MM400, we obtained the desired Ftivazide compound **1** in 70% yield after 30 min of milling, 90% after 60 min (^1^H NMR analysis), and quantitatively after 90 min of milling. When operating in P7, the reaction times were shorter: 90% yield after 30 min and quantitative obtention of Ftivazide **1** after 60 min of milling (Table 1).

Overall, 4-Hydroxy-N’-[(E)-(4-hydroxy-3-methoxyphenyl)methylidene]benzohydrazide **2** was obtained in 84% yield after 90 min of milling in the MM400, while it was obtained quantitatively after the same time of milling in the P7 (Table 1). The introduction of sodium acetate and/or pyrogenic S13 silica did not modify the time of the reaction needed.

The molecular structure of these two hydrazide-hydrazones was confirmed by spectral methods. In the IR spectra, the carbonyl groups give characteristic bands at 1642 cm^−1^ (C=O), 1594 cm^−1^ (C=N), and 3034 cm^−1^ (N-H) for compound **1** and 1635 cm^−1^ (C=O), 1583 cm^−1^ (HC=N), 2969 cm^−1^ (N-H), for compound **2**. In the ^1^H NMR spectra (DMSO-d6), we can observe that compounds **1** (as we have previously reported [29]) and **2** are each present in two isomeric forms EE’ (trans-E) and EZ’ (cis-E), in a ratio of 90/10. Isomeric forms were also recently reported by Kargar et al. [31] when coupling 2-hydroxy5-chloro benzaldehyde with isoniazid.

For the major form in DMSO-d6, we identify the characteristic peaks at 11.38 ppm (N-H) and 8.35 ppm (HC=N) for compound **1** and at 11.46 ppm (N-H) and 8.31 ppm (HC=N) for compound **2**. In the ^13^C NMR spectra (DMSO-d6), the prominent characteristic peaks resonate at 161.77 (C=O), 150.03 (HC=N) for compound **1**, and 162.92 (C=O), 147.86 (HC=N) for compound **2**.

#### 2.1.2. Aging

**Figure 3:**
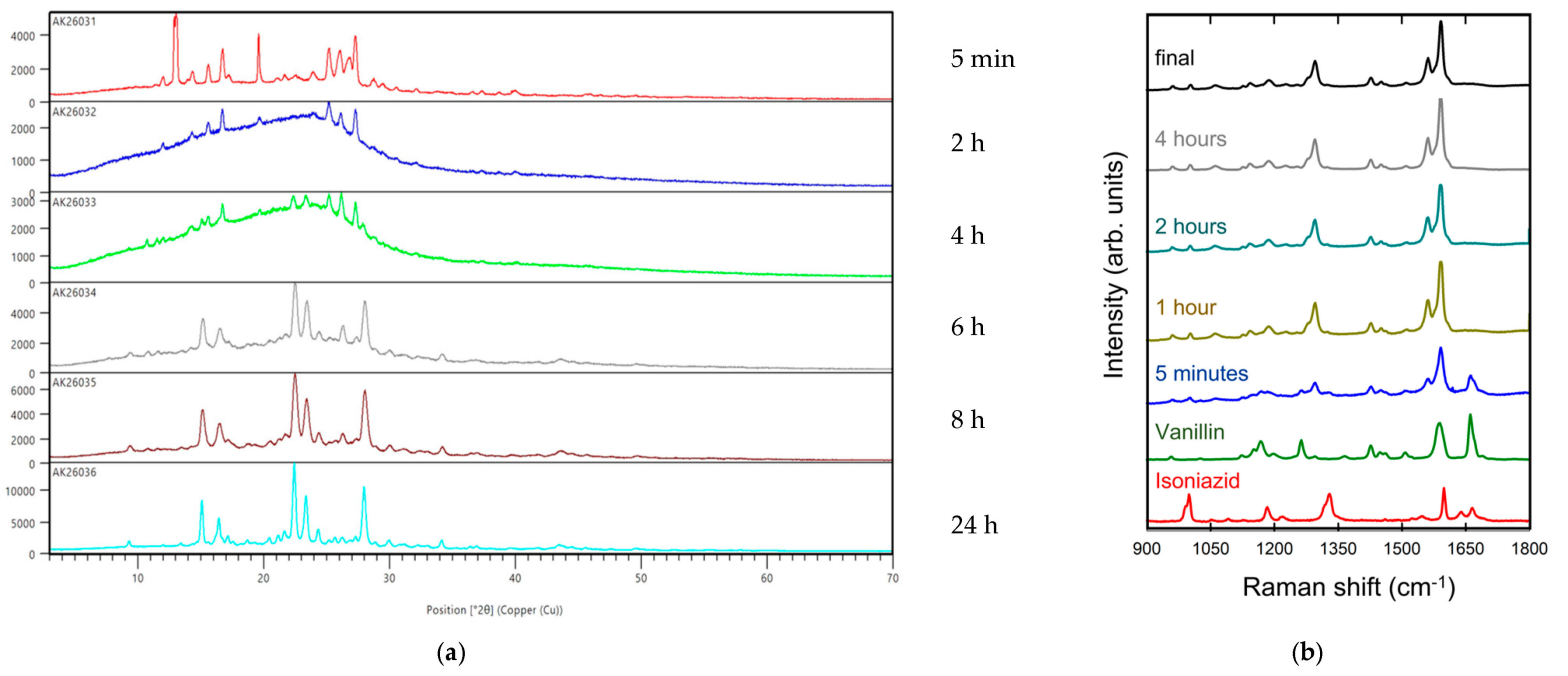
(**a**) A powder XRD study of progression in time of the aging process for the compound **1**; (**b**) A Raman spectroscopy study of progression in time of the aging process for the compound **1**.

Some chemical reactions can occur spontaneously between solid reagents, in some cases facilitated by humidity, organic vapors, the addition of catalysts, heating, or brief grinding [32]. We tried to see if, in the case of Ftivazide, the aging operates after a brief grinding. In both cases, no reaction occurs when vanillin and hydrazinamide are mixed without grinding. Without any initial energy input, when mixing the reactants and leaving them at 37 °C, we observe after 7 h less than 5% conversion to the corresponding hydrazones-hydrazides. On the contrary, for compound **1**, when grinding together vanillin and isoniazid for 5 min in the MM400 (30% conversion by ^1^H NMR) or 5 min in the P7 (50% conversion by ^1^H NMR) and then leaving each amorphous yellowish solid obtained in sealed tubes at 37 °C, the aging phenomenon occurs. The aging reaction issued from the MM400 experiment was followed every 2 h for 24 h by DRX, Raman, and ^1^H NMR spectroscopies. On the XRD spectra (Figure 3a), we can observe the substantial modification of the XRD patterns over time, going from reagents through an amorphous system to a final crystalline one. On the Raman spectra (Figure 3b), we can also observe the variation of the prominent peaks, mainly, the C=O of the aldehyde that disappears with time. At the same time, the C=N band appears very close to the carbonyl of the isoniazid.

**Figure 4:**
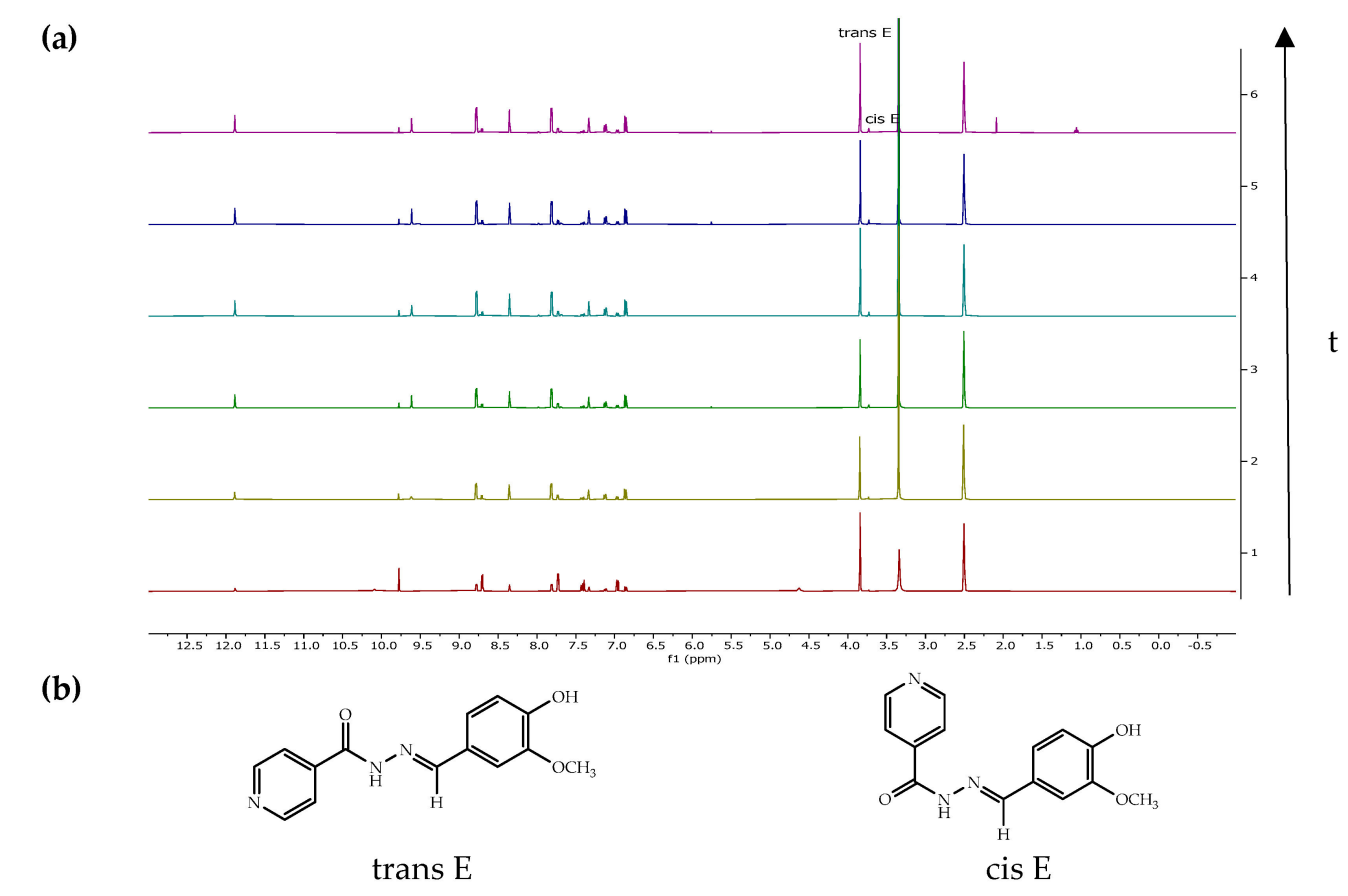
(**a**) A ^1^H NMR study of progression in time of the aging process for the compound **1:** red—analysis of the reaction mixture after 5 min of grinding; yellow—after 5 min of grinding and 2 h of aging; green—after 5 min of grinding and 4 h of aging; light blue—after 5 min of grinding and 6 h of aging; blue—after 5 min of grinding and 8 h of aging; violet—after 5 min of grinding and 8 h of aging; (**b**) two geometric isomers of compound **1**.

In the ^1^H NMR spectra (Figure 4a), we can see the transformation of the starting compounds present in the initial mixture to the final hydrazone-hydrazide **1**. It is noteworthy that the ratio between the two isomeric forms trans-E and cis-E is always the same (90/10) and remains the same whether we grind the system for 90 min or leave the aging process to operate (Figure 4b).

### 2.2. Hydrazones Bearing the 5-Nitrofuran-2-acrylaldehyde Frame

#### Synthesis and Characterization

The 5-nitrofuran-2-acrylaldehyde is also an important aldehyde in constructing biologically active hydrazones. When reacting with hydrazines, it produces 5-nitrofuryl hydrazones with important biological activities. As mentioned before, Nifurzide (Figure 1d) is an intestinal anti-infective. Additionally, 5-Nitrofuryl containing thiosemicarbazones were reported, in 2004, by Aguirre et al. [33] to act against the protozoan parasite *Trypanosoma cruzi*, the causative agent of Chagas disease, as were their platinum-based complexes reported later by Vieites et al. [34]. Moreover, 5-Nitro-2-furyl containing adamantanealkanohydrazones were reported in 2016 by Foskolos et al. [35] as promising compounds with potent in vitro trypanocidal activity.

**Scheme 1:**
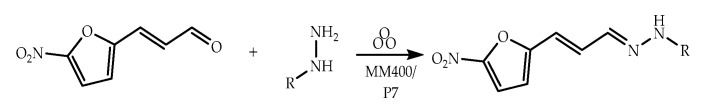
Generalized scheme for coupling 4-nitrophenyl acrolein with specified hydrazines.

In that respect, we report the mechanochemical synthesis of five hydrazones obtained by coupling 5-nitrofuran-2-acrylaldehyde with isoniazid, 1-hydrazinophthalazine hydrochloride, 2-hydrazinobenzothiazole, rhodanine, and indole-3-acetic hydrazide affording the corresponding hydrazones **3**–**7** in very good to excellent yields (Scheme 1).

All syntheses have been performed in the MM400 at 30 Hz, using the same material as before and grinding all reactions for 3 × 30 min (total of 90 min). All reactions were conducted in a 1:1 ratio between the two reactants. For the reaction with 1-hydrazinophthalazine hydrochloride, we also introduced 1eq. of sodium acetate, as we have already reported [27]. After 90 min of grinding, the compounds issued from coupling with 1-hydrazinophthalazine hydrochloride, 2-hydrazinobenzothiazole and indole-3-acetic hydrazide (compounds **4**, **5**, **7**, respectively) showed the total conversion of the aldehyde on TLC and ^1^H NMR and were obtained quantitatively. Identical results were obtained when operating in P7 (3 cycles of 30 min). When using MM400, the reaction with isoniazid gave an aldehyde conversion of 78%, while with 3-aminorhodanine, the aldehyde conversion was 75%. Both compounds were purified and obtained in 70% (compound **3**) and 63% (compound **6**) yields. Finally, for both reactions operating in the P7 (6 cycles of 30 min), we obtained a quantitative aldehyde transformation and 98% and 97% yield of the corresponding hydrazones **3** and **6** (Table 2).

A detailed analysis of all compounds was undertaken by NMR. All NMR spectra were recorded on a Bruker Avance NEO 600 spectrometer equipped with a 5 mm broadband inverse triple resonance probe ^1^H BB (31P-103Rh)/31P with Z field gradients. All chemical shifts for ^1^H and ^13^C are relative to TMS using ^1^H (residual). The results are reported in the Supplementary Materials in Tables S1a and S2a.

Compounds **3, 4**, **6** and **7** were analyzed at 298 K while compound **5** at 378 K. All the ^1^H and ^13^C signals were assigned based on the chemical shifts, spin-spin coupling constants, splitting patterns, and signal intensities using ^1^H-^1^H COSY 45, ^1^H-^13^C HSQC, and ^1^H-^13^C HMBC. A gradient-enhanced ^1^H COSY45 was realized which included four scans for per increment. ROESY spectra were recorded with a mixing time of 300 ms, ^1^H-^13^C correlation spectra using a gradient-enhanced HSQC sequence (delay was optimized for ^1^J_CH_ of 145 Hz) was obtained with eight scans per increment. A gradient-enhanced HMBC experiment was performed allowing 62.5 ms for long-range coupling evolution (32 scans were accumulated). Typically, 1024 t2 data points were collected for 256 t1 increments.

For all compounds, the 5-nitrofuran-2-acryle hydrazone frame presents comparable characteristics in terms of chemical shifts and multiplicities. For the nitrofuranyl system, the chemical shifts are between 7.65 and 7.80 ppm for the C_4_-H hydrogen atom and 115.48–116.09 ppm for the C_4_ carbon atom. For the C_3_-H position, the chemical shifts are between 7.06 and 7.21 ppm for the hydrogen atom and 113.27 and 114.21 ppm for the C_4_ carbon atom.

The acryl hydrazone frame (C_6_–C_8_) presents similar chemical shifts for compounds **3** and **4**. The ^1^H chemical shifts for C_6_H, C_7_H, C_8_H are 7.16, 7.16, 8.27 ppm (compound **4**) and 7.08, 7.26, 8.28 ppm (compound **3**), respectively. The ^13^C chemical shifts for C_6_H, C_7_H, C_8_H are 125.19, 130.74, 149.56 ppm (compound **3**) and 122.83, 132.23, 153.36 ppm (compound **4**), respectively.

Compound **5** has very broad peaks at 298 K for all hydrogen and carbon atoms of the benzothiazole frame. This is also the case to a lesser extent when we operate at an elevated temperature (378 K). At 378 K, we can identify all peaks except the quaternary C_18_ and C_19_ carbon atoms. This is due to the slow rotation even at 378 K around the C_11_-N_10_ bond.

Compound **6** is present in two forms where rotation is restricted under the NMR time scale and temperature operating. NMR experiments show separate hydrogen resonances for each isomer if the rate constant of interconverting isomers (10^−1^ to 10^3^ s^−1^) is within the NMR timescale [36]. Separate proton signals allow 2D ROESY techniques to detect such isomers. In ROESY, protons undergoing chemical exchange show the same phase as the diagonal [37] (see the Supporting Information for ROESY spectra). The two **6**:**6′** forms are in a ratio of 90:10.

For compound **7**, we also observe two forms where rotation is restricted, as the 2D ROESY techniques demonstrated (see the Supporting Information for ROESY spectra). The two **7**:**7′** forms are in a ratio of 52:48. The 2D experiments permitted the identification of all peaks. It is particularly interesting to point out that the main differences in chemical shifts between the two forms are observed for C_8_H (7.82/143.60 ppm vs. 8.01/146.82 ppm), C_11_ (173.33 vs. 167.91 ppm), and C_12_H (3.98/29.32 vs. 3.63/32.05 ppm).

### 2.3. Hydrazones Bearing the 5-(4-Nitrophenyl)-2-furaldehyde Frame

#### 2.3.1. Synthesis and Characterization

The 5-(4-nitrophenyl)-2-furaldehyde is one of the essential aldehydes in creating biologically active hydrazones. The most prominent example compound is the muscle relaxant dantrolene. Its mechanochemical synthesis and study have already been reported by Colacino et al. [24], Crawford et al. [38], and Sović et al. [39]. Dantrolene-like hydrazide and hydrazone analogs have also been reported recently by Bolognino et al. [40] as multitarget agents for neurodegenerative diseases.

**Scheme 2:**
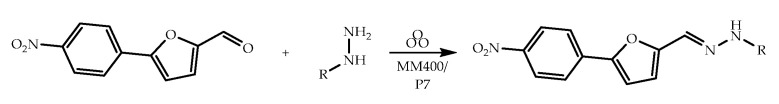
Generalized scheme for coupling 5-(4-nitrophenyl)-2-furaldehyde with specified hydrazines.

In that regard, we convey the mechanochemical synthesis of ten hydrazones obtained by coupling 5-(4-nitrophenyl)-2-furaldehyde with various hydrazinamides and hydrazines (Scheme 2).

All syntheses have been performed in MM400, grinding, in general, all starting materials for 3 × 30 min (total of 90 min). We have also performed some of the reactions in P7. As reported previously by us [25], concerning the formation of hydrazones with phenolic aldehydes, the weakest conversion was in a reaction with 3-aminorhodanine. This was also the case when reacting 5-(4-nitrophenyl)-2-furaldehyde with 3-aminorhodanine. The conversion to the corresponding hydrazone **11** was only 8% after 90 min of milling in the MM400, while it had increased up to 37% when operating in the planetary P7 apparatus. By continuing the grinding process, we obtained much better results. When operating in the planetary P7 and after six cycles of 30 min each (6 × 30 min) the desired hydrazone was obtained in 93% yield (97% aldehyde conversion).

So, for all reactions studied on a MM400 or a P7 apparatus, we operated through three cycles of 30 min each (3 × 30 min) and, when necessary, through six cycles of 30 min each (6 × 30 min), which was sufficient for all other reactions studied. Table 3 indexes all hydrazinamides and hydrazines coupled with 5-(4-nitrophenyl)-2-furaldehyde, the apparatus used, the time for the reaction, the % conversion of the aldehyde, and the yield of the hydrazone obtained.

Except for 3-aminorhodanine discussed before, three other hydrazinamides were used. The reaction of isoniazid with 5-(4-nitrophenyl)-2-furaldehyde (3 × 30 min) afforded the corresponding hydrazone **8** (>98% aldehyde conversion) in the MM400 and quantitative yield in the P7 apparatus. Reaction with indole-3-acetic hydrazide afforded virtually similar results; hydrazone-hydrazide **12** was obtained with 95% aldehyde conversion on the MM400 (yield 70%). Coupling with benzyloxycarbonyl hydrazide to obtain **17** was much less efficient. The aldehyde conversion was only at 40% after 3 × 30 min on the MM400 apparatus (yield 35%). Gratifyingly, when operating in P7 (six cycles of 30 min), we obtain a quantitative aldehyde transformation and excellent yields for compounds **12** (98% yield) and **17** (97% yield).

The aging process was also experimented with for compound **8** when operating in the P7 apparatus. While this is effective for compound **1** as we mentioned earlier, no reaction advance was observed in the case of compound **8** after 5 min of grinding (25% of aldehyde conversion).

The coupling reaction was also conducted with six hydrazines. For the reaction with 1-hydrazinophthalazine hydrochloride, we also introduced 1eq. of sodium acetate, as previously mentioned, for the 5-nitrofuranyl derivatives. Reactions with 1-hydrazinophthalazine hydrochloride, 2-hydrazinobenzothiazole and 5-bromo-2-hydrazinopyridine afforded excellent aldehyde conversions (95–99%) after 3 × 30 min in the MM400. The same was true when operating in the P7 apparatus simultaneously (3 × 30 min). The yields of the hydrazones obtained after purification varied from 80% to 95%. Reaction with three other hydrazines, namely 6-chloro-2-hydrazino-1,3-benzoxazole, 2-hydrazinylpyrazine, and 2-hydrazino-4-(trifluorométhyl) pyrimidine, gave less % of aldehyde conversion after 3 × 30 min of grinding in the MM400 apparatus (90%, 80%, and 60% aldehyde conversion, respectively) and yields varying from 47 to 60%. Again, when using the P7 apparatus for 6 × 30 min, we obtained excellent conversions and yields between 85% and 95%. It is essential to point out that for all reactions when the conversions are >95%, the silica gel Puriflash purifications afforded better yields because solubility problems can be better circumvented.

As before, a detailed analysis by NMR (in DMSO-d6) was undertaken for all compounds of this series (see Tables S1 and S2 of ^1^HNMR and ^13^C NMR data in the Supporting Information). Compounds **8**, **9**, **11** and **16** are present in a single form; no geometric or rotational isomers are observed. Compound **12**, as is the case of the previously discussed compound **7**, is present in two forms (ratio 50:50) in which rotation is restricted under the NMR time scale and temperature. Finally, compounds **13**, **14**, **15**, and **17** are present in two isomeric forms that do not exchange even at higher temperatures (348 K). The ratio determined are 82:18 (compound **13**), 78:22 (compound **14**), 70:30 (compound **15**), and 84:16 (compound **17**).

#### 2.3.2. Compound 8: X-ray Structure

Various attempts have been made to crystallize some hydrazones bearing the 5-(4-nitrophenyl)-2-furaldehyde frame (assays on compounds **8** and **9**). Solvents and a mixture of solvents used were methanol, acetonitrile, and mixtures of different ratios of each with dimethyl sulfoxide. Usually, 20 mg of hydrazone was introduced in 6 mL of solvent (or a mixture of solvents) and heated at 70 °C before cooling the solution. Compound **8** was recrystallized in acetonitrile (or methanol)/dimethyl sulfoxide (12:1 ratio); a single crystal was thus obtained and analyzed.

**Figure 5:**
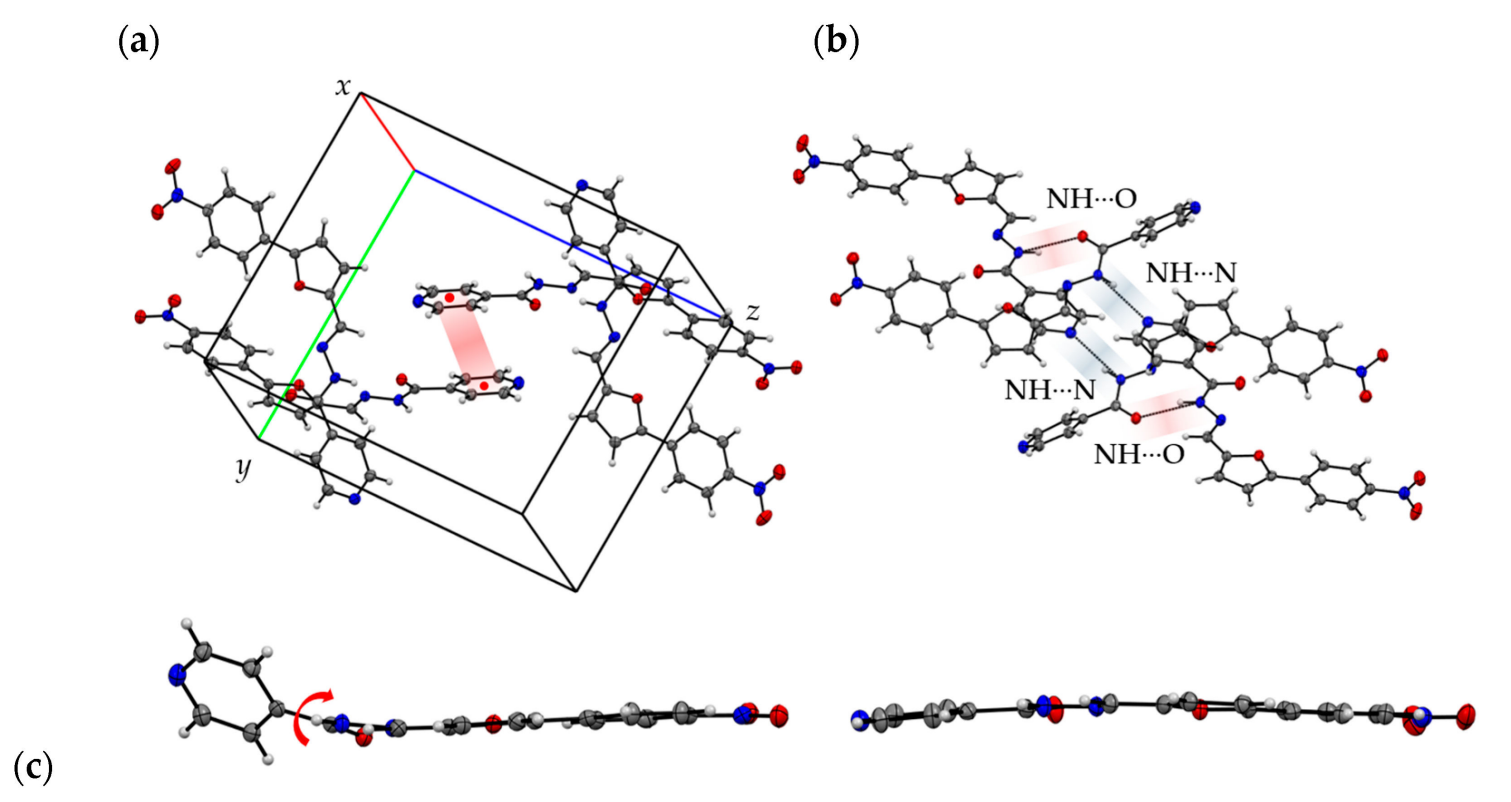
Crystal lattice and molecular arrangement of **8**: (**a**) *π-π* stacking interactions are highlighted in red, the centroids of the rings represented by red dots; (**b**) NH···O (red) and NH···N (blue) hydrogen-bonded of molecules **8** forming tetrameric arrangement; (**c**) two types of molecular geometries of molecule **8** present in a crystal lattice: pyridine ring rotation along C_9_-C_10_ bond is shown on the right, and a flat molecule on the left side of figure.

Compound **8** forms triclinic crystals of space group *P*$\bar{1}$. The crystal lattice consists of two symmetry-independent molecules, from which one adopts a nearly planar arrangement (Figure 5). In contrast, the second molecule deviates from the planarity by out-of-plane rotation of one of the aromatic pyridine rings along the C_9_-C_10_ bond. This is due to the presence of strong NH···N, NH···O hydrogen bonds and relatively weak *π-π* stacking interactions between neighboring molecules in the molecular environment (Figure 5).

Variable temperature structural resolutions have been also performed for single crystal of **8** at five individual temperature points between 100 and 300 K at atmospheric pressure (Table 4). Upon heating from 100 K to 300 K it expands its volume by nearly 3%, which is typical for molecular organic solids. The single crystal has been subject to heating and cooling in order to evaluate if there are any changes in the crystal lattice. No significant structural transformations have been detected, and the two forms of compound **8** remained unchanged.

In Table 5, we have reported as a function of the temperature, the measurements of the most relevant interactions. The *π-π* stacking interactions between neighboring molecules indicated weak centroid distance and strong centroid angle differences ranging from 3.817 Å (and 67.76°) at 100 K to 3.897 Å (and 39.73°) at 300 K. The NH···N hydrogen bonds ranged from 2.9019(16) Å at 100 K to 2.9451(17) Å at 300 K while the NH···O hydrogen bonds were relatively weak. Finally, the dihedral angle N_8_-C_9_-C_10_-C_15_ remains nearly equal to 37°.

## 3. Biological Activities

The in vitro anti-infectious activities of all synthesized compounds were determined regarding eight pathogens: one parasite, namely *Leishmania donovani* and seven bacteria: *Mycobacterium tuberculosis* H37Rv, *Staphylococcus aureus* (ATCC25923 and ATCC29213), *Escherichia coli* (ATCC25922), *Klebsiella pneumoniae* (BAA 1705), *Acinetobacter baumannii* (BAA 1605), *Pseudomonas aeruginosa* (ATCC 27853), and *Enterococcus* spp. All results are included in Table 6 along with the reference drugs: miltefosine for *L. donovani*, isoniazid, streptomycin for *M. tuberculosis,* and ciprofloxacin for other antibacterial activities. Concerning the antileishmanial activities, the molecules were tested against LV9 *L. donovani* axenic amastigote forms and intramacrophage amastigote forms. Cytotoxicity was evaluated against macrophage RAW 264.7 cells and expressed as CC_50_. The calculated Selectivity Index (SI) corresponds to the ratio CC_50_/IC_50_ on intramacrophage amastigote forms.

### 3.1. Cytotoxicity

Cytotoxicity is expressed as CC_50_ (Cytotoxic Concentration 50%). No compound was cytotoxic on RAW 264.7 macrophages at 100 µM.

### 3.2. Activities against Leishmania donovani

*Activities against the axenic forms.* All compounds were first evaluated in vitro on the axenic form of *L. donovani*, the parasite responsible for visceral leishmaniasis in humans, to check their intrinsic antileishmanial activity. The most active compound (**9**) exhibited an IC_50_ value at 0.2 µM. Five compounds had IC_50_ values less than 1 µM. Five compounds exhibited IC_50_ values in a range 1–5 µM, four compounds in a range 5–20 µM, two compounds in a range 20–100 µM and one compound was inactive at 100 µM.

*Activities against the intramacrophage amastigote forms.* Then, they were evaluated on the *L. donovani* intramacrophage amastigote model, which is closer to the pathological conditions. The most active compound was compound **9** which was also the most active on the axenic forms. Its IC_50_ value was 0.3 µM whereas the IC_50_ value of miltefosine was 2.2 µM. This series is promising as five compounds exhibited IC_50_ values less than 1 µM, one compound had an IC_50_ in a range 1–5 µM, three compounds in a range 5–20 µM, five compounds in a range 20–100 µM, and three compounds were inactive at 100 µM. There is a globally good correlation between the results obtained from the axenic and intramacrophage amastigotes.

Compound **9** also had the highest selectivity index within this series. Presently, compound **9** should be evaluated in vivo on the *L. donovani*/BALB/c mouse model.

### 3.3. Activities against Mycobacterium tuberculosis H37Rv

All compounds were tested in vitro on the wild type strain *M. tuberculosis H37Rv*. Six of the tested compounds (**1**, **3**, **5**, **6**, **7,** and **8**) were found to be active on *M. tuberculosis H37Rv* with a minimum inhibitory concentration (MIC_90_) between 0.6 and 7.4 µM (Table 6). These results bring some interest to the selected compounds.

### 3.4. Activities against other Microorganisms

Antimicrobial activities of the compounds against *S. aureus* ATCC25923 or ATCC29213 *aureus* showed that compounds **3** and **7** and to a lesser extent compound **6** possess interesting MIC values of 1.75 μM (or 7 μM) for compound **3** and 0.7 μM (or 3 μM) for compound **7** and 13 μM (or 7 μM) for compound **6** (Table 6), the results comparable to the MIC of the ciprofloxacin, the control antibiotic. Concerning activities against the strain ATCC25922 of *E. coli*, compounds **3** (28 μM) and **7** (47 μM) possess some activity but 40–60 times inferior to the control antibiotic.

No activities were found against *K. pneumoniae*, *A. baumannii*, *P. aeruginosa,* and *Enterococcus spp*. for the compounds synthesized. All compounds exhibited no activities for concentrations >256 µg/mL.

## 4. Conclusions

A series of 17 hydrazones have been synthetized by mechanochemical means. The fragments chosen were phenolic and furanyl aldehydes coupled with 12 heterocyclic hydrazines or hydrazinamides. All compounds can be obtained quantitatively when operating with a planetary ball mill and a maximum reaction time of 180 min (six cycles of 30 min each). Complete spectroscopic analyses of hydrazones revealed eight compounds (**3**–**5**, **8**–**11**, **16**) present in one form, six compounds (**1**, **2**, **13**–**15**) present in two isomeric forms, and three compounds (**6**, **7**, **12**) where one rotation is restricted giving rise to two different forms. It should be interesting to explore possible ways of purifying the different geometric and rotationally restricted forms. From a mechanochemistry point of view, it should be important to study and understand the factors that can influence the aging process operating for compound **1** but not for compound **8**. The single crystal X-ray structure of one of the hydrazones bearing the isoniazid fragment (**8**) was obtained. It indicates that the crystal lattice consists of two symmetry-independent molecules, from which one adopts a nearly planar arrangement, while the second molecule deviates from the planarity by out-of-plane rotation of one of the aromatic pyridine rings. All compounds obtained were tested for antileishmanial and antibacterial activities. Four compounds (**1**, **3**, **5** and **8**) showed good activities against *M. tuberculosis*, one (**7**) was very potent against *S. aureus* and merits further evaluation. Most interestingly, this series displayed very promising antileishmanial activity. Among all, compound **9** exhibited an IC_50_ value of 0.3 µM on the *L. donovani* intramacrophage amastigote in vitro model and a good selectivity index, better than miltefosine, making it merit an in vivo evaluation.

The extension of the chemical library of hydrazones by mechanochemical means in addition to their potential transformations (triazoles, azines...) should be an important issue for the obtention of new biologically active compounds under green chemistry processes.

## 5. Materials and Methods

### 5.1. Reagents Used

All chemicals were obtained from TCI, Aldrich, Alfa Aesar, Fluka, Apollo Scientific, Acros Organics, Carlo Erba Reagents, and BLDPharm 80–99% and used without further purification. The melting points of each compound were measured on a SMP3 apparatus (Barloworld Scientific, Staffordshire, UK).

### 5.2. NMR Analysis

^1^H and ^13^C nuclear magnetic resonance (NMR) was performed using on a Bruker Avance NEO 600 spectrometer equipped with a 5 mm broadband inverse triple resonance probe ^1^H BB (31P-103Rh)/31P with Z field gradients and Bruker 400 MHz spectrometers (except indication for 300 MHz) with IconNMR automation software that allows fully automated acquisitions. All chemical shifts for ^1^H and ^13^C were expressed in parts per million (ppm) relative to TMS using ^1^H (residual). DMSO-d_6_ was used as solvent (except indication).

### 5.3. Powder X-ray Diffraction (XRD) Analysis

The XRD analysis of a given reaction mixture was performed at defined time periods, before, during, and after the reaction process. The analysis was performed using the Miniflex600 powder diffractometer (Rigaku) equipped with a Cu anode (K-Alpha1 [Å]1.54060), a fast 1D detector with energy selection and a 6-position autosampler. The range in 2θ was 5 to 70°, the step between each point was 0.01°, the time per point was 1 s. For all compounds obtained, the XRD spectra were recorded (see Supplementary Materials) after operating under the optimal reaction conditions for each hydrazone.

### 5.4. X-ray Single Crystal

A series of low-temperature single crystal X-ray diffraction experiments were performed using an XtaLAB Synergy-S Rigaku diffractometer equipped with a hybrid photon counting Hypix-6000HE detector and Cu (λ = 1.5406 Å) microsource. The crystal was cooled down to 100 K using an Oxford Cryosystems 800 cryostream cooler device and measured at increasing temperature up to 300 K with a step of 50 K. The CrysAlisPro program suite was used for pre-experiment, data collection, determination of the UB matrices, and initial data reduction [41]. Crystal structures of **2** were solved and refined using SHELXS and SHELXL programs [42]. Hydrogen atoms were located from geometry after each refinement cycle with *U*_iso_ = 1.2*U*_eq_ of their carriers.

### 5.5. RAMAN Analysis

RAMAN acquisitions were taken on XploRA PLUS confocal Raman microscope—HORIBA using several laser wavelengths and different resolutions.

### 5.6. Fourier Transform Infrared Spectrometry (FTIR) Analysis

For Fourier transform infrared spectroscopy (FTIR), spectra were taken on Nicolet 6700 Thermoscientific (Waltham, MA, USA)—ATR diamond-resolution 4 cm^−1^, 16 scans, DLaTGS detector or on the versatile Perkin Elmer Frontier MIR/FIR infrared spectrometer with resolution better than 0.4 cm.

### 5.7. Mass Spectrometry Analysis

For mass spectrometry, samples were injected on DSQ II mass spectrometer (Thermo Fisher Scientific, Waltham, MA, USA) equipped with electron impact (EI) and chemical ionization (DCI NH_3_) sources and UPLC Xevo G2 Q TOF (Waters) and GCT Premier (Waters) for high resolution mass spectrometry.

### 5.8. Biological Tests

#### 5.8.1. Evaluation of Compound Cytotoxicity on RAW 264.7

Macrophages Cytotoxicity was evaluated on RAW 264.7 macrophages using the resazurin method as previously detailed [43].

#### 5.8.2. Evaluation of the Antileishmanial Activity

*L. donovani* (MHOM/ET/67/HU3, also called LV9) promastigotes and axenic amastigotes were maintained according to the protocols previously described [43].

In vitro antileishmanial evaluation on *L. donovani* axenic amastigotes. This evaluation was performed using the SYBR Green method as previously described. IC_50_ values were calculated using the ICEstimator version 1.2 software (http://www.antimalarial-icestimator.net/runregression1.2.htm, accessed on 5 April 2023). Miltefosine was used as the reference drug.

In vitro antileishmanial evaluation on intramacrophage amastigotes. RAW 264.7 macrophages were infected with *L. donovani* axenic amastigotes according to a ratio of 10 parasites per macrophage. In these conditions, the percentage of infected macrophages was around 80%, and the mean number of amastigotes per infected macrophage was 4 to 5 in the untreated controls. The in vitro treatment was applied 24 h post-infection, and the treatment duration was 48 h. The results of the effect of the compounds are given as percentage of parasite growth reduction, measured using the SYBR Green incorporation method. The activity of the compounds is expressed as IC_50_, calculated using the ICEstimator version 1.2 software [43]. Miltefosine was used as the reference drug.

#### 5.8.3. MIC Determination for *Mycobacterium tuberculosis* H37Rv

*Mycobacterium tuberculosis* H37Rv reference strain was used. This strain was grown at 37 °C in Middlebrook 7H9 broth (Difco, Becton Dickinson, Franklin Lakes, NJ, USA), supplemented with 0.05% *w*/*v* Tween 80 or on Middlebrook 7H10 (Difco, Becton Dickinson, Franklin Lakes, NJ, USA), both supplemented with 0.2% *w*/*v* glycerol and 10% *v/v* Middlebrook OADC enrichment (oleic acid, albumin, d-glucose, catalase; Becton Dickinson, Franklin Lakes, NJ, USA). The antibiotic resistance profile of H37Rv was obtained by the resazurin-based microtiter assay (REMA). All the tested compounds were dissolved in DMSO (Sigma Aldrich, St. Louis, MO, USA). Streptomycin (Duchefa Biochemie, Haarlem, The Netherlands) was dissolved in water.

Drug susceptibility of *M. tuberculosis* H37Rv was determined using the REMA method [44]. Log-phase cultures were diluted at concentrations of approximately 10^5^ bacteria/mL. Then, 100 μL of bacterial suspensions was added in a 96-well black plate (Fluoronunc, Thermo Fisher, Waltham, MA, USA) containing 100 μL of Middlebrook 7H9, without the addition of Tween 80, in the presence of serial compound dilution. Growth controls containing no compound and sterile controls without inoculum were also included. A volume of 10 μL of resazurin (0.025% *w*/*v*) was added to each well after 7 days of incubation at 37 °C, and bacterial viability was assessed after a further overnight incubation using a FluoroskanTM Microplate Fluorometer (Thermo Fisher Scientific, Waltham, MA, USA; excitation = 544 nm, emission = 590 nm). Bacterial viability was calculated as a percentage of resazurin turnover in the absence of compound (internal negative control). Experiments were performed in duplicate at least two times.

#### 5.8.4. MIC Determination for Antimicrobial Activities on *S. aureus* and *E. coli*

The effectiveness of compounds against *S. aureus* ATCC25923 and *E. coli* ATCC 25922, was assessed determining MICs in Mueller-Hinton Broth II cation adjusted by the 2-fold microdilution method in U-bottom 96-well microtiter plates and inoculating about 10^5^ CFU. The microtiter plates were incubated at 37 °C for 24, and growth was determined by the resazurin method [45]; the solution of resazurin sodium salt (Sigma Aldrich, St. Louis, MO, USA) was prepared at 0.01% in distilled water and filter-sterilized. Thirty microliters of resazurin solution were added to each well, and the microtiters were reincubated at 37 °C for 4 h. The MIC was defined as the lowest concentration of the drug that prevented a change in color from blue to pink, which indicates bacterial growth.

#### 5.8.5. MIC Determination for Antimicrobial Activities on *K. pneumoniae*, *A. baumannii*, *P. aeruginosa*, and *Enterococcus* spp.

All tests and MIC determinations were carried out according to the literature [46].

### 5.9. Synthesis

General procedure A. Aldehyde and hydrazine (molar ratio 1:1) were neatly grinded in a vibratory ball-mill MM400, equipped with two zirconium dioxide 10 mL jars (internal Ø 20 mm), each jar was equipped with 2 balls of 10 mm Ø, working frequency at 30 Hz, for 30–90 min. Depending on the conversion of the reaction the mixture was either purified on a Puriflash system or (when conversion >95%) washed with a small quantity of ethyl acetate then methanol (5–10 mL each) and dried.

General procedure B. Aldehyde and hydrazine (molar ratio 1:1) were neatly ground in a planetary ball-mill Pulverisette 7 (P7) (Fritsch), equipped with two zirconium dioxide 20 mL bowls (45 mm inner Ø), each bowl equipped with 5 balls of 10 mm Ø. The experiment was performed at 800 rpm for 30–90 min. As before, purification was conducted for reaction conversion <95%.

For all reactions when hydrazine hydrochlorides were used one equivalent of sodium acetate was also introduced.

Aging procedure. The method was implemented for the synthesis of Ftivazide on both the MM400 mixer-mill and the P7 planetary mill. The mixture of vanillin and isoniazid (1:1) was neatly ground for 10 and 5 min, respectively. The resulting reaction mixture in both cases was an amorphous yellowish substance. Analysis of this substance by ^1^H NMR showed a conversion of the starting products of 30 and 50%, respectively. The recovered amorphous substance was sealed in a glass tube and placed under a controlled thermal regime (T = 37 °C) for 24 h with monitoring every 2 h. After 24 h the conversion (and yield) was determined using ^1^H NMR of 90%. Finally, to accelerate the process, the solid mixture was heated at 80 °C for 3 h. A final conversion of >99% was thus reached.

N′-[(E)-(4-Hydroxy-3-methoxyphenyl)methylene]isonicotinohydrazide **1**. This compound was synthesized using general procedure A (1.04 mmol of vanillin)—reaction time 3 × 10 min (70%); reaction time 3 × 30 min (yield 99%); aging procedure—reaction time 27 h (>99%). Using general procedure B (2.76 mmol of vanillin)—reaction time 3 × 10 min (90%); reaction time 6 × 10 min (>99%).

**m.p.:** 232.5 °C. **Rf:** 0.46 DCM/MeOH (9:1).

**^1^H NMR (400 MHz, DMSO-d6) δ ppm:** 11.88 (s, 1H), 9.77 (s, 0.1 H), 8.81–8.75 (m, 2H), 8.73 (m, 0.2H), 8.35 (s, 1H), 7.98 (s, 0.1H), 7.84–7.78 (m, 2H), 7.68 (m, 0.2H), 7.39 (d, J = 1.9 Hz, 0.1H), 7.33 (d, J = 1.9 Hz, 1H), 7.12 (dd, J = 8.2, 1.9 Hz, 1H), 7.07 (d, J = 1.9 Hz, 0.1H), 6.86 (d, J = 8.1 Hz, 1H), 6.78 (d, J = 8.1 Hz, 0.1H), 3.84 (s, 3H), 3.73 (s, 0.3H).

**^13^C NMR (100 MHz, DMSO-d6) δ ppm:** 161.77 (C=O), 150.76 (CH), 150.03 (CH), 149.77 (C), 148.54 (C), 141.16 (C), 125.86 (C), 122.90 (CH), 121.95 (CH), 115.94 (CH), 109.56 (CH), 56.04 (CH_3_).

**^13^C NMR CP-MAS (100 MHz, Probe: 4G Vr = 8 kHz) δ ppm:** 160.91, 152.49, 149.35, 147.00, 137.60, 128.03, 122.90, 114.08, 107.22, 55.10.

**^15^N NMR CP-MAS (40 MHz, Probe: 4G Vr = 8 kHz) δ ppm:** 306.66 (s, 1N, =N-), 298.68 (s, 1N, N_Ar_), 169.76 (s, 1N, -NH-).

**FTIR *v* cm^−1^:** 3034 (N-H), 3075 (O-H), 3050.13 (C=C), 1643 (C=O), 1594 (HC=N), 1551, 1500, 752 (=C-H), 1251 (C-O), 1210 (C-O), 1328 (O-H).

MS (DCI-NH_3_) *m/z:* 272.1 [M+H^+^], 289.1 [M+NH_4_^+^].

**HRMS (ES, TOF, MeOH) *m/z:*** calc. for C_14_H_14_N_3_O_3_ **[M+H^+^] =** 272.1035; found: 272.1037.

4-hydroxy-N′-[(E)-(4-hydroxy-3-methoxyphenyl)methylidene]benzohydrazide **2**. This compound was synthesized using general procedure A (0.99 mmol of vanillin)—reaction time 3 × 30 min with 93% aldehyde conversion. Crude product purified with FCC using DCM:MeOH 9:1, compound **2** was obtained in 84% yield. General procedure B (2.03 mmol of vanillin)—reaction time 3 × 30 min. Aldehyde conversion was total (>99%) and compound **2** was obtained in 96%.

**m.p.:** 217.4–218.9 °C. **Rf:** 0.40 (DCM:MeOH 9:1).

**^1^H NMR (600 MHz, DMSO-d6) δ ppm:** 11.46 (s, 1H), 8.31 (s, 1H), 7.79 (d, *J* = 8.3 Hz, 2H), 7.30 (s, 1H), 7.06 (d, J = 8.2 Hz, 1H), 6.84 (m, 3H), 3.83 (s, 3H).

**^13^C NMR (150 MHz, DMSO-d6) δ ppm:** 162.99 (C=O), 161.06 (C), 149.30 (C), 148.49 (C), 147.86 (CH), 130.01 (CH), 126.36 (C), 124.51 (C), 122.43 (CH), 115.88 (CH), 115.45 (CH), 109.26 (CH), 55.99 (CH_3_).

**^13^C NMR CP-MAS** (100 MHz, Probe: 4G Vr = 10 kHz) δ ppm: 159.77, 146.66, 124.37, 113.85, 105.03, 53.61.

**FTIR *v* cm^−1^:** 3257 (O-H), 2969 (N-H), 848 (=C-H), 1635 (C=O), 1583 (HC=N), 1455 (C=C), 1259 (C-NH), 1244 (=C-O-C=), 1176 (C-O).

**MS (DCI-NH_3_)** *m/z*: 287.1 [M+H^+^], 304.1 [M+NH_4_^+^].

**HRMS (ES, TOF, MeOH) *m/z:*** calc. for C_15_H_14_N_2_O_4_ **[M+H^+^] =** 287.1032; found: 287.1030.

N′-[(1E,2E)-3-(5-Nitro-2-furyl)-2-propen-1-ylidene]isonicotinohydrazide **3**. The compound was synthesized using general procedure A (0.99 mmol 3-(4-nitro-furyl)-acrolein)—reaction time 3 × 30 min. Aldehyde conversion 78%. Crude product was purified with FCC using gradient DCM:MeOH 95:5 -> 9:1 (70%). When procedure B was used (1.2 mmol for each reactant), we obtained after 6 × 30 min grinding an aldehyde conversion > 99%; compound **3** was obtained in 98% yield after washing.

**m.p.:** 252.1–254.0 °C. **Rf:** 0.64 (DCM:MeOH 9:1).

**^1^H NMR (600 MHz, DMSO) δ ppm:** 12.20 (s, 1H), 8.81–8.77 (m, 2H), 8.27 (dd, J = 6.9, 1.9 Hz, 1H), 7.85–7.80 (m, 2H), 7.79 (d, J = 4.0 Hz, 1H), 7.21 (d, J = 4.0 Hz, 1H), 7.15–7.18 (m, 2H).

**^13^C NMR (150 MHz) δ ppm:** 162.17 (C=O), 155.21 (C), 151.91 (C), 150.84 (CH), 149.55 (C), 140.62 (C), 130.75 (CH), 125.20 (CH), 122.02 (CH), 115.84 (CH), 114.21 (CH).

**^13^C NMR CP-MAS (100 MHz, Probe: 4G Vr = 8 kHz) δ ppm:** 167.29, 154.17, 150.77, 146.15, 142.75, 140.81, 128.66, 123.61, 121.13, 114.57.

**FTIR *v* cm^−1^:** 3108 (=C-H), 2964 (N-H), 1604 (C=C), 1416 (=C-H), 1672 (C=O), 1583 (HC=N), 1370 (C-NO_2_), 1251 (=C-O-C=).

**MS (DCI-NH_3_)** *m/z*: 287.1 [M+H^+^], 304.1 [M+NH_4_^+^].

**HRMS (ES, TOF, MeOH) *m/z:*** calc. for C_13_H_10_N_4_O_4_ **[M+H^+^] =** 287.0780; found: 287.0786.

2-{(2E)-2-[(2E)-3-(5-Nitro-2-furyl)-2-propen-1-ylidene]hydrazino}phthalazine **4**. General procedure A: 5-(nitrophenyl)-furan-2carbaldehyde, 1-hydrazinophthalazine hydrochloride, and CH_3_COONa (1:1:1) were used for the reaction (for 0.67 mmol of each reagent)—reaction time 3 × 30. Aldehyde conversion 99%; compound **4** was obtained in 99% yield. General procedure B: 1.50 mmol of 5-(nitrophenyl)-furan-2carbaldehyde, 1.50 mmol of 1-hydrazinophthalazine hydrochloride and 1.50 mmol of CH3COONa (1:1:1)—reaction time 3 × 30. Aldehyde conversion 99%; compound **4** was obtained in 99% yield.

**m.p.:** 235 °C. **Rf:** 0.34 (DCM:AcOEt 92:8).

**^1^H NMR (600 MHz, DMSO-d6) δ ppm:** 12.21 (s, 1H), 8.31 (d, J = 7.9 Hz, 1H), 8.28 (d, J = 9.7 Hz, 1H), 8.20 (s, 1H), 7.84–7.75 (m, 4H), 7.26 (dd, J = 16.0, 9.7 Hz, 1H), 7.10 (s, 1H), 7.07 (d, J = 4.0 Hz, 1H).

**^13^C NMR (150 MHz, DMSO-d6) δ ppm:** 155.70 (C), 153.35 (CH), 151.78 (C), 149.56 (C), 139.07 (CH), 133.24 (CH), 132.52 (CH), 132.21 (CH), 127.68 (CH), 127.10 (CH), 127.09 (C), 124.40 (CH), 122.82 (CH), 116.10 (CH), 114.03 (CH).

**^13^C NMR CP-MAS (100 MHz, Probe: 4G Vr = 8 kHz) δ ppm:** 153.69, 149.56, 144.70, 134.49, 131.33, 123.56, 120.88, 113.11.

**FTIR *v* cm^−1^:** 3127, 3151 (=C-H), 2959 (N-H), 1602 (C=C), 1593 (HC=N), 1568 (C-NO_2_), 1281 (=C-O-C=).

**MS (DCI-NH_3_)** *m/z*: 310.0 [M+H^+^].

**HRMS (ES, TOF, MeOH) *m/z:*** calc. for C_15_H_11_N_5_O_3_ **[M+H^+^] =** 310.0940; found: 310.0945.

2-{(2E)-2-[(2E)-3-(5-Nitro-2-furyl)-2-propen-1-ylidene]hydrazino}-1,3-benzothiazole **5**. The compound was synthesized using general procedure A (0.90 mmol 3-(4-nitro-furyl)-acrolein)—reaction time 3 × 30 min. Aldehyde conversion 99% (yield 99%). Identical results were obtained with general procedure B (1.5 mmol scale).

**m.p.:** 243.1 °C. **Rf:** 0.38 (DCM:MeOH 98:2).

**^1^H NMR (600 MHz, DMSO-d6) δ ppm:** 8.00 (d, J = 9.3 Hz, 1H), 7.74 (d, J = 7.8 Hz, 1H), 7.65 (d, J = 3.9 Hz, 1H), 7.46 (d, J = 8.0 Hz, 1H), 7.31 (t, J = 7.6 Hz, 1H), 7.13 (t, J = 7.6 Hz, 1H), 7.12–7.08 (m, 1H), 7.06 (d, J = 3.9 Hz, 1H), 6.95 (d, J = 16.0 Hz, 1H).

**^13^C NMR (150 MHz, DMSO-d6) δ ppm:** 167.22 (C), 155.73 (C), 151.86 (C), 145.46 (CH), 130.92 (CH), 126.45 (CH), 122.41 (CH), 122.17 (CH), 121.95 (CH), 118.04 (CH), 115.48 (CH), 113.24 (CH).

**^13^C NMR CP-MAS (100 MHz, Probe: 4G Vr = 8 kHz) δ ppm:** 166.32, 153.44, 150.53, 147.85, 143.97, 128.66, 126.72, 120.88, 116.75, 115.05, 112.87.

**FTIR *v* cm^−1^:** 3151, 3127, 3055 (=C-H), 3023 (NH), 1626 (C=C), 1602 (HC=N), 1571 (C-NO_2_), 1351 (C-N), 1241 (=C-O-C=), 1126 (C=S).

**MS (DCI-NH_3_)** *m/z*: 315.1 [M+H^+^].

**HRMS (ES, TOF, MeOH) *m/z:*** calc. for C_14_H_10_N_4_O_3_S **[M+H^+^] =** 315.0522; found: 315.0554.

3-{(E)-[(2E)-3-(5-Nitro-2-furyl)-2-propen-1-ylidene]amino}-2-thioxo-1,3-thiazolidin-4-one **6**. The compound was synthesized using general procedure A (0.95 mmol 3-(4-nitro-furyl)-acrolein)—reaction time 3 × 30 min. Aldehyde conversion 75%. Crude product was purified with FCC using a mixture of (DCM:AcOEt 8:2):PE 6:4 (63%). Procedure B (1.2 mmol of each reactant) afforded after 6 × 30 min a total aldehyde conversion (>99%); after washing compound **6** was obtained in 97% yield.

**m.p.:** 85 °C. **Rf:** 0.41 ((DCM:AcOEt 8:2):PE 6:4).

**^1^H NMR (600 MHz, DMSO) δ ppm:** 8.63 (d, J = 9.7 Hz, 0.1H), 8.59 (d, J = 9.6 Hz, 1H), 7.81 (d, J = 3.9 Hz, 1H), 7.79 (d, J = 3.9 Hz, 0.1H), 7.51 (d, J = 16.0 Hz, 1H), 7.44 (d, J = 15.5 Hz, 0.1H)., 7.41 (d, J = 4.0 Hz, 1H), 7.38 (d, J = 4.0 Hz, 0.1H), 7.26 (dd, J = 16.0, 9.6 Hz, 1H), 6.88 (dd, J = 15.5, 9.7 Hz, 0.1H), 4.35 (s, 2H), 4.23 (s, 0.2H).

**^13^C NMR (150 MHz) δ ppm:** 197.45 (C=S), 196.90 (C=S), 170.03 (C=O), 169.97 (CH), 169.62 (C=O), 168.99 (CH), 153.80 (C), 153.42 (C), 152.77 (C), 152.47 (C), 132.45 (CH), 132.06 (CH), 127.87 (CH), 122.18 (CH), 117.93 (CH), 116.51 (CH), 115.38 (CH), 115.25 (CH), 36.01 (CH_2_), 35.23 (CH_2_).

**^13^C NMR CP-MAS (100 MHz, Probe: 4G Vr = 8 kHz) δ ppm:** 199.61, 196.69, 171.91, 170.45, 168.99, 161.70, 150.53, 132.79, 126.72, 123.56, 114.81, 36.57.

**FTIR *v* cm^−1^:** 3128 (=C-H), 1728 (C=O), 1627 (C=C), 1511 (C=N), 1349 (C-NO_2_), 1222 (=C-O-C=), 1096 (C-N), 1096 (C=S).

**MS (DCI-NH_3_)** *m/z*: 297.9 [M+H^+^].

**HRMS (DCI-CH_4_, MeOH) *m/z***: calc. for C_10_H_7_N_3_O_4_S_2_ **[M+H^+^]** = 297.9965; found: 297.9956.

3-(1H-indol-2-yl)-N′-[(1E,2E)-3-(5-nitrofuran-2-yl)prop-2-en-1-ylidene]acetohydrazide **7**. The compound was synthesized using general procedure A (0.84 mmol 3-(4-nitro-furyl)-acrolein)—reaction time 3 × 30 min. Aldehyde conversion 99%; compound **7** was obtained in 99% yield. Identical results were obtained with general procedure B (1.6 mmol scale)

**m.p.:** 219.6–221.6 °C. **Rf:** 0.90 (DCM:MeOH 9:1).

**^1^H NMR (600 MHz, DMSO-d6) δ ppm:** 11.70 (s, 1H), 11.42 (s, 1H), 10.93 (s, 1H), 10.89 (s, 1H), 8.03–8.00 (m, 1H), 7.83 (d, J = 9.3 Hz, 1H), 7.76 (d, J = 4.0 Hz, 1H), 7.75 (d, J = 4.0 Hz, 1H), 7.56 (d, J = 7.9 Hz, 1H), 7.54 (d, J = 7.9 Hz, 1H), 7.35 (t, J = 8.3 Hz, 2H), 7.24 (d, J = 2.4 Hz, 1H), 7.22 (d, J = 2.4 Hz, 1H), 7.12 (t, J = 4.3 Hz, 2H), 7.03–7.11 (m, 5H), 6.96–7.02 (m, 3H), 3.98 (s, 1H), 3.63 (s, 1H).

**^13^C NMR (150 MHz, DMSO-d6) δ ppm:** 173.32 (C=O), 167.91 (C=O), 155.40 (C), 155.33 (C), 151.77 (C), 146.81 (CH), 143.60 (CH), 136.53 (C), 136.43 (C), 131.03 (CH), 130.84 (CH), 127.81 (C), 127.54 (C), 124.56 (CH), 124.46 (CH), 123.97 (CH), 123.35 (CH), 121.54 (CH), 121.41 (CH), 119.16 (CH), 119.05 (CH), 118.93 (CH), 118.83 (CH), 115.95 (CH), 115.88 (CH), 113.85 (CH), 111.87 (CH), 111.79 (CH), 108.32 (C), 108.24 (C), 31.16 (CH_2_), 29.32 (CH_2_).

**^13^C NMR CP-MAS (100 MHz, Probe: 4G Vr = 8 kHz) δ ppm:** 175.07, 152.71, 150.77, 142.27, 134.73, 126.47, 120.88, 117.24, 112.62, 110.19, 107.76, 26.85.

**FTIR *v* cm^−1^:** 3058 (=C-H), 2949 (NH), 1660 (C=O), 1586 (HC=N), 1379 (C-NO_2_), 1240 (=C-O-C=).

**MS (DCI-NH_3_)** *m/z*: 339.1 [M+H^+^], 356.0 [M+NH_4_^+^].

**HRMS (ES, TOF, MeOH) *m/z:*** calc. for C_17_H_14_N_4_O_3_ **[M+H^+^] =** 339.1093; found: 339.1094.

N′-{(E)-[5-(4-Nitrophenyl)-2-furyl]methylene}isonicotinohydrazide **8**. The compound was synthesized using general procedure A (0.85 mmol of 5-(4-nitrophenyl)furan-2-carbaldehyde)—reaction time 3 × 30 min with aldehyde conversion > 98%; after washing, compound **8** was obtained in 98% yield. General procedure B (2.76 mmol of 5-(4-nitrophenyl)furan-2-carbaldehyde)—reaction time 3 × 30 min. Aldehyde conversion > 99%; after washing, compound **8** was obtained in 99% yield.

**m.p.:** 262.3 °C (DSC). **Rf:** 0.48 DCM/MeOH (9:1).

**^1^H NMR (600 MHz, DMSO-d6) δ ppm:** 12.19 (s, 1H), 8.80 (d, J = 5.8 Hz, 2H), 8.42 (s, 1H), 8.32 (d, J = 8.9 Hz, 2H), 8.05 (d, J = 8.8 Hz, 2H), 7.83 (d, J = 5.8 Hz, 2H), 7.50 (d, J = 3.7 Hz, 1H), 7.21 (d, J = 3.6 Hz, 1H).

**^13^C NMR (150 MHz, DMSO-d6) δ ppm:** 162.15 (C=O), 153.21 (C), 151.13 (C), 150.87 (CH), 146.87 (C), 140.78 (C), 138.53 (CH), 135.57(C), 125.20 (CH), 125.04 (CH), 121.99 (CH), 117.51 (CH), 112.96 (CH).

**^13^C NMR CP-MAS (100 MHz, Probe: 4G V r= 8 kHz) δ ppm:** 165.01, 158.45, 152.86, 150.19, 148.97, 146.30, 144.35, 139.01, 137.06, 133.17, 124.91, 122.47, 122.15, 119.56, 116.15, 111.05.

**FTIR *v* cm^−1^:** 3051 (=C-H), 2927 (N-H), 1662 (C=O), 1599 (HC=N), 1513, 1330 (C-NO_2_), 1110 (C-O), 850, 750 (=C-H).

**MS (DCI-NH_3_)** *m/z*: 337.1 [M+H^+^], 354.1 [M+NH_4_^+^].

**HRMS (ES, TOF, MeOH) *m/z:*** calc. for C_17_H_12_N_4_O_4_ **[M+H^+^] =** 337.0937; found: 337.0936.

1-[(2E)-2-{[5-(4-Nitrophenyl)-2-furyl]methylene}hydrazino]phthalazine **9**. The compound was synthesized using modified general procedure A, 0.61 mmol of 5-(nitrophenyl)-furan-2carbaldehyde, 0.61 mmol of 1-hydrazinophthalazine hydrochloride, and 0.61 mmol of CH_3_COONa (1:1:1) were used for the reaction,—reaction time 3 × 30 min. Obtained powder was washed with EtOH (5 mL) and water (5 mL) to recover acetic acid and sodium chloride then filtered; the obtained precipitate was dried under pressure. Aldehyde conversion 95%. Crude product was purified with FCC using gradient DCM ->DCM:MeOH 99:1 (85%). When general procedure B (1 mmol of each reactant) was used in the presence of CH_3_COONa (1:1:1) we obtained >99% aldehyde conversion and after washing 91% yield of compound **9**.

**m.p.:** 239.5–241.5 °C. **Rf:** 0.48 DCM/MeOH (99:1).

**^1^H NMR (600 MHz, DMSO-d6) δ ppm:** 12.33 (s, 0.2H), 12.15 (s, 1H), 8.47 (s, 0.2H), 8.35 (s, 1H), 8.33–8.30 (m, 3H), 8.20 (s, 0.2H), 8.19 (s, 1H), 8.13 (d, J = 8.5 Hz, 2H), 8.07 (d, J = 8.6 Hz, 0.4H), 7.87–7.82 (m, 0.8 H), 7.81 (d, J = 7.3 Hz, 1H), 7.74–7.79 (m, 2H), 7.54 (d, J = 3.7 Hz, 0.2H), 7.50 (d, J = 3.7 Hz, 1H), 7.28 (d, J = 3.7 Hz, 1H).

**^13^C NMR (150 MHz, DMSO-d6) δ ppm:** 153.25 (C), 152.47 (C), 149.40 (C), 146.56 (C), 141.85 (CH), 138.73 (CH), 135.98 (C), 133.07 (CH), 132.43 (CH), 127.58 (C), 127.05 (CH), 126.36 (C), 124.98 (CH), 124.93 (CH), 124.31 (CH), 115.66 (CH), 113.19 (CH).

**^13^C NMR CP-MAS (100 MHz, Probe: 4G Vr = 8 kHz) δ ppm:** 154.90, 149.56, 143.97, 138.38, 133.76, 130.12, 125.74, 124.77, 122.10, 111.41, 109.22.

**FTIR *v* cm^−1^:** 3081 (=C-H), 2974 (N-H), 1609, 1327 (HC=N), 1598 (C=C), 1532, 1342 (C-NO_2_), 1251 (=C-O-C=), 750 (=C-H).

**MS (DCI-NH_3_)** *m/z*: 360.1 [M+H^+^].

**HRMS (ES, TOF, MeOH) *m/z:*** calc. for C_19_H_13_N_5_O_3_ **[M+H^+^] =** 360.1097; found: 360.1100.

*2-[(2E)-2-{[5-(4-Nitrophenyl)-2-furyl]methylene}hydrazino]-1,3-benzothiazole* **10**. The compound was synthesized using general procedure A (0.78 mmol 5-(nitrophenyl)-furan-2carbaldehyde)—reaction time 3 × 30 min. Aldehyde conversion 95%. Crude product was purified with FCC using DCM:MeOH 95:5 (84%). When general procedure B was used (1.2 mmol each reactant) for 3 × 30 min, we obtained >99% aldehyde conversion and, after washing, 92% yield of compound **10**.

**m.p.:** 271.9 °C. **Rf:** 0.62 (DCM:MeOH 95:5).

**^1^H NMR (600 MHz, DMSO-d6) δ ppm:** 8.30 (d, J = 8.9 Hz, 2H), 8.13 (s, 1H), 8.01 (d, J = 9.0 Hz, 1H), 7.73 (d, J = 7.8 Hz, 1H), 7.43 (d, J = 7.9 Hz, 1H), 7.35 (d, J = 3.7 Hz, 1H), 7.31 (t, J = 7.1 Hz, 1H), 7.12 (t, J = 5.1 Hz, 1H), 7.01 (d, J = 3.7 Hz, 1H).

**^13^C NMR (150 MHz, DMSO-d6) δ ppm:** 167.12 (C), 152.60 (C), 152.03 (C), 149.20 (C), 147.13 (C), 135.95 (CH), 134.64 (C), 129.20 (C), 126.42 (CH), 124.97 (CH), 124.81 (CH), 122.22 (CH), 121.95 (CH), 117.60 (CH), 114.68 (CH), 112.68 (CH).

**^13^C NMR CP-MAS (100 MHz, Probe: 4G Vr = 8 kHz) δ ppm:** 167.29, 150.77, 147.85, 144.70, 133.28, 129.63, 124.29, 122.10, 120.88, 114.57, 109.95.

**FTIR *v* cm^−1^:** 3074 (=C-H), 2961 (N-H), 1894 (C=S), 1609 (HC=N), 1598 (C=C), 1527, 1326 (C-NO_2_), 1326 (HC=N), 1107 (=C-O-C=).

**MS (DCI-NH_3_)** *m/z*: 365.1 [M+H^+^].

**HRMS (ES, TOF, MeOH) *m/z:*** calc. for C_18_H_12_N_4_O_3_S **[M+H^+^] =** 365.0708; found: 365.0704.

3-[(E)-{[5-(4-Nitrophenyl)-2-furyl]methylene}amino]-2-thioxo-1,3-thiazolidin-4-one **11**. General procedure A (0.82 mmol 5-(nitrophenyl)-furan-2carbaldehyde)—reaction time 3 × 30 min. Aldehyde conversion 8%; using general procedure B (2.19 mmol 5-(nitrophenyl)-furan-2carbaldehyde)—reaction time 3 × 30 min. Aldehyde conversion 37%. General procedure B (1.91 mmol)—reaction time 6 × 30 min. Aldehyde conversion 97% and yield after washing 93%. For other reactions, compound **11** was purified with FCC using gradient M1:PE 4:6 -> 1:0 (M1 = DCM:AcOEt 8:2).

**m.p.:** 175 °C (DSC). **Rf:** 0.26 (DCM).

**^1^H NMR (600 MHz, DMSO-d6) δ ppm:** 8.65 (s, 1H), 8.36 (d, J = 8.9 Hz, 2H), 8.12 (d, J = 8.9 Hz, 2H), 7.62 (d, J = 3.7 Hz, 1H), 7.58 (d, J = 3.8 Hz, 1H), 4.36 (s, 2H).

**^13^C NMR (150 MHz, DMSO-d6) δ ppm:** 197.32 (C=S), 170.14 (C=O), 158.46 (CH), 155.55 (C), 148.51 (C), 147.54 (C), 134.91 (C), 125.99 (CH), 125.10 (CH), 124.15 (CH), 113.08 (CH), 35.27 (CH_2_).

**^13^C NMR CP-MAS (100 MHz, Probe: 4G Vr = 8 kHz) δ ppm:** 168.99, 155.63, 145.91, 133.52, 124.04, 109.46, 31.96.

**FTIR *v* cm^−1^:** 3116 (=C-H), 2955 (N-H), 1598 (C=C), 1730 (C=O), 1332 (=C-O-C=), 1688 (HC=N), 1556 (C-NO_2_), 1227 (C=S).

**MS (DCI-NH_3_)** *m/z*: 348.0 [M+H^+^].

**HRMS (DCI-CH_4_, MeOH) *m/z:*** calc. for C_14_H_9_N_3_O_4_S_2_ **[M+H^+^] =** 348.0113; found: 348.0113.

3-(1H-indol-2-yl)-N′-[(E)-[5-(4-nitrophenyl)furan-2-yl]methylidene]acetohydrazide **12**. The compound was synthesized using general procedure A (0.74 mmol 5-(nitrophenyl)-furan-2carbaldehyde)—reaction time 3 × 30 min. Aldehyde conversion 95%. Crude product was purified with FCC using DCM:MeOH 99:1 (yield 70%). When procedure B was used (1.2 mmol, 3 × 30 min) the aldehyde conversion > 99%, and compound **12** was obtained in 98% yield.

**m.p.:** 248–249 °C. **Rf:** 0.63 (DCM:MeOH 9:1).

**^1^H NMR (600 MHz, DMSO-d6) δ ppm:** 11.67 (s, 1H), 11.42 (s, 1H), 10.95 (s, 1H), 10.92 (s, 1H), 8.33 (d, J = 8.9 Hz, 2H), 8.31 (d, J = 8.9 Hz, 2H), 8.20 (s, 1H), 8.05 (d, J = 8.9 Hz, 2H), 8.01 (d, J = 8.8 Hz, 2H), 7.93 (s, 1H), 7.67 (d, J = 7.9 Hz, 1H), 7.58 (d, J = 8.0 Hz, 1H), 7.48 (d, J = 3.7 Hz, 1H), 7.45 (d, J = 3.6 Hz, 1H), 7.36 (d, J = 7.4 Hz, 1H), 7.34 (d, J = 8.3 Hz, 1H), 7.28 (d, J = 2.4 Hz, 1H), 7.26 (d, J = 2.4 Hz, 1H), 7.12–7.07 (m, 3H), 7.05 (t, J = 7.5 Hz, 1H), 7.00 (t, J = 7.3 Hz, 2H), 6.94 (t, J = 7.5 Hz, 1H), 4.06 (s, 2H), 3.66 (s, 2H).

**^13^C NMR (150 MHz, DMSO-d6) δ ppm:** 173.28 (C=O), 167.78 (C=O), 153.08 (C), 152.42 (C), 151.50 (C), 151.47 (C), 146.74 (C), 146.72 (C), 136.58 (C), 136.42 (C), 135.98 (CH), 135.76 (C), 135.67 (C), 132.30 (CH), 127.84 (C), 127.56 (C), 125.08 (CH), 125.03 (CH), 124.90 (CH), 124.60 (CH), 124.43 (CH), 121.54 (CH), 121.38 (CH), 119.26 (CH), 119.09 (CH), 118.91 (CH), 118.76 (CH), 116.45 (CH), 115.81 (CH), 112.93 (CH), 112.89 (CH), 111.87 (CH), 111.78 (CH), 108.43 (C), 108.41 (C), 32.20 (CH_2_), 29.57 (CH_2_).

**^13^C NMR CP-MAS (100 MHz, Probe: 4G Vr = 8 kHz) δ ppm:** 177.25, 150.04, 144.94, 142.27, 138.38, 136.43, 133.52, 126.96, 120.88, 118.70, 111.17, 105.58, 27.82.

**FTIR *v* cm^−1^:** 3079 (=C-H), 1659 (C=O), 1597 (HC=N), 1358 (C-NO_2_), 1220 (=C-O-C=).

**MS (DCI-NH_3_)** *m/z*: 389.0 [M+H^+^], 406.0 [M+NH_4_^+^].

**HRMS (ES, TOF, MeOH) *m/z:*** calc. for C_21_H_16_N_4_O_4_ **[M+H^+^] =** 389.1250; found: 389.1252.

5-Bromo-2-[(2E)-2-{[5-(4-nitrophenyl)furan-2-yl]methylidene}hydrazin-1-yl]pyridine **13**. The compound was synthesized using general procedure A (0.74 mmol 5-(nitrophenyl)-furan-2carbaldehyde)—reaction time 3 × 30 min. Aldehyde conversion 99%. Compound **13** was obtained in 95% yield. Procedure B (1.2 mmol each reactant, 3 × 30 min) afforded identical results.

**m.p.:** 241.4–243.3 °C. **Rf:** 0.76 (DCM:AcOEt 9:1).

**^1^H NMR (600 MHz, DMSO-d6) δ ppm:** 11.31 (s, 1H), 10.29 (s, 0.2H), 8.36 (d, J = 8.9 Hz, 0.4H), 8.33 (d, J = 2.4 Hz, 0.2H), 8.31 (d, J = 8.9 Hz, 2H), 8.23 (d, J = 2.4 Hz, 1H), 8.07 (d, J = 8.7 Hz, 0.4H), 8.01 (d, J = 9.0 Hz, 2H), 7.99 (s, 1H), 7.91 (dd, J = 8.9, 2.5 Hz, 0.2H), 7.86 (dd, J = 9.0, 2.5 Hz, 1H), 7.55 (d, J = 3.8 Hz, 0.2H), 7.45 (d, J = 3.6 Hz, 1H), 7.41 (s, 0.2H), 7.27 (d, J = 8.9 Hz, 0.2H), 7.23–7.19 (m, 1.2H), 6.99 (d, J = 3.6 Hz, 1H).

**^13^C NMR (150 MHz, DMSO-d6) δ ppm:** 156.22 (C), 155.93 (C), 152.41 (C), 152.38 (C), 151.77 (C), 149.64 (C), 148.66 (CH), 146.99 (C), 146.44 (C), 141.12 (CH), 140.86 (CH), 135.91 (C), 135.35 (C), 129.85 (CH), 126.35 (CH), 125.16 (CH), 125.03 (CH), 124.68 (CH), 117.57 (CH), 113.85 (CH), 113.15 (CH), 112.78 (CH), 110.58 (CH), 109.42 (CH), 109.33 (CH), 108.81 (CH).

**^13^C NMR CP-MAS (100 MHz, Probe: 4G Vr = 8 kHz) δ ppm:** 152.47 151.74, 149.56, 147.37, 144.45, 139.35, 132.30, 126.72, 124.53, 122.34, 116.51, 113.11, 108.98.

**FTIR *v* cm^−1^:** 3201, 838 (=C-H), 2918 (N-H), 1505 (HC=N), 1323 (NO_2_), 1026 (=C-O-C=), 691 (C-Br).

**MS (DCI-NH_3_)***m/z*: 387.0; 389.0 [M+H^+^].

**HRMS (ES, TOF, MeOH) *m/z:*** calc. for C_16_H_11_BrN_4_O_3_ **[M+H^+^] =** 387.0093; found: 387.0094.

*2-[(2E)-2-{[5-(4-Nitrophenyl)furan-2-yl]methylidene}hydrazin-1-yl]-4-(trifluoromethyl) pyrimidine* **14**. The compound was synthesized using general procedure A (0.76 mmol 5-(nitrophenyl)-furan-2carbaldehyde)—reaction time 3 × 30 min. Aldehyde conversion 60%. Crude product was purified with FCC using DCM:AcOEt 9:1 (47%). Procedure B (1.2 mmol each reactant, 6 × 30 min) afforded >99% aldehyde conversion and after washing compound **14** was obtained in 90% yield.

**m.p.:** 212.4–214.2 °C. **Rf:** 0.33 (DCM:AcOEt 9:1).

**^1^H NMR (600 MHz, DMSO-d6) δ ppm:** 11.98 (s, 1H), 10.93 (s, 0.3H), 8.90 (d, J = 4.9 Hz, 0.3H), 8.85 (d, J = 4.9 Hz, 1H), 8.35 (d, J = 8.9 Hz, 0.6H), 8.32 (d, J = 9.0 Hz, 2H), 8.14 (s, 1H), 8.08 (d, J = 8.9 Hz, 0.6H), 8.02 (d, J = 8.9 Hz, 2H), 7.58 (s, 0.3H), 7.55 (d, J = 3.7 Hz, 0.3H), 7.47 (d, J = 3.7 Hz, 1H), 7.41 (d, J = 4.9 Hz, 0.3H), 7.31 (d, J = 4.9 Hz, 1H), 7.27 (d, J = 3.7 Hz, 0.3H), 7.07 (d, J = 3.7 Hz, 1H).

**^13^C NMR (150 MHz, DMSO-d6) δ ppm:** 162.65 (CH), 162.54 (CH), 160.99 (C), 160.24 (C), 155.25 (q, J = 34.8 Hz, C), 152.67 (C), 152.44 (C), 151.86 (C), 149.15 (C), 147.12 (C), 146.64 (C), 135.75 (C), 135.32 (C), 133.64 (CH), 130.56 (CH), 125.32 (CH), 125.05 (CH), 124.97 (CH), 124.93 (CH), 121.06 (q, J = 271.0 Hz, C), 118.67 (CH), 115.43 (CH), 113.09 (CH), 112.68 (CH), 109.62 (CH), 108.62 (CH).

**^13^C NMR CP-MAS (100 MHz, Probe: 4G Vr = 8 kHz) δ ppm:** 158.06, 156.36, 154.66, 151.99, 150.53, 149.31, 144.21, 134.25, 132.55, 129.63, 126.80–118.61 (m), 115.30, 112.14, 109.95, 107.76.

**FTIR *v* cm^−1^:** 3049, 850 (=C-H), 1511 (HC=N), 1328 (NO_2_), 1105 (=C-O-C=), 1134 (C-F).

**MS (DCI-NH_3_)** *m/z*: 377.9 [M+H^+^].

**HRMS (ES, TOF, MeOH) *m/z:*** calc. for C_16_H_10_F_3_N_5_O_3_ **[M+H^+^] =** 378.0825; found: 378.0821.

2-[(2E)-2-{[5-(4-nitrophenyl)furan-2-yl]methylidene}hydrazin-1-yl]pyrazine **15**. The compound was synthesized using general procedure A (0.76 mmol 5-(nitrophenyl)-furan-2carbaldehyde)—reaction time 3 × 30 min. Aldehyde conversion 80%. Crude product was purified with FCC using gradient DCM:AcOEt 9:1 -> 5:5 (60%).%). Procedure B (1.2 mmol each reactant, 6 × 30 min) afforded 97% aldehyde conversion, and after washing compound **15**, was obtained in 95% yield.

**m.p.:** 272.2–274.0 °C. **Rf:** 0.24 (DCM:AcOEt 9:1).

**^1^H NMR (600 MHz, DMSO-d6) δ ppm:** 11.41 (s, 1H), 10.36 (s, 0.4H), 8.64 (s, 0.4H), 8.63 (s, 1H), 8.36 (d, J = 9.0 Hz, 0.8H), 8.31 (d, J = 8.8 Hz, 2H), 8.25 (s, 0.4H), 8.16–8.14 (m, 1.4H), 8.08 (d, J = 8.9 Hz, 0.8H), 8.05–8.03 (m, 3H), 8.01 (s, 1H), 7.55 (d, J = 3.7 Hz, 0.4H), 7.47 (s, 0.4H), 7.45 (d, J = 3.6 Hz, 1H), 7.25 (d, J = 3.8 Hz, 0.4H), 7.04 (d, J = 3.6 Hz, 1H).

**^13^C NMR (150 MHz, DMSO-d6) δ ppm:** 153.19 (C), 152.90 (C), 152.55 (C), 152.13 (C), 151.94 (C), 149.38 (C), 147.04 (C), 146.49 (C), 142.46 (CH), 137.03 (CH), 135.94 (CH), 135.88 (C), 135.32 (C), 131.71 (CH), 131.17 (CH), 130.78 (CH), 127.64 (CH), 125.26 (CH), 125.02 (CH), 124.80 (CH), 117.99 (CH), 114.28 (CH), 113.06 (CH), 112.73 (CH).

**^13^C NMR CP-MAS (100 MHz, Probe: 4G Vr = 10 kHz) δ ppm:** 151.50, 149.80, 143.72, 138.62, 134.00, 130.36, 123.80, 121.37, 114.57, 108.49.

**FTIR *v* cm^−1^:** 3092, 850 (=C-H), 1597 (HC=N), 1323 (NO_2_), 1107 (=C-O-C=).

**MS (DCI-NH_3_)** *m/z*: 310.0 [M+H^+^].

**HRMS (ES, TOF, MeOH) *m/z:*** calc. for C_15_H_11_N_5_O_3_ **[M+H^+^] =** 310.0940; found: 310.0946.

6-chloro-2-[(2E)-2-{[5-(4-nitrophenyl)furan-2-yl]methylidene}hydrazin-1-yl]-1,3-benzoxazole **16**. The compound was synthesized using general procedure A (0.75 mmol 5-(nitrophenyl)-furan-2-carbaldehyde)—reaction time 3 × 30 min. Aldehyde conversion 90%. Crude product was purified with FCC using DCM:AcOEt 9:1 (54%). Procedure B (1.2 mmol each reactant, 6 × 30 min) afforded >99% aldehyde conversion and after washing compound **16** was obtained in 95% yield.

**m.p.:** 190.9 °C. **Rf:** 0.65 (DCM:AcOEt 9:1).

**^1^H NMR (600 MHz, DMSO-d6) δ ppm:** 12.40 (s, 1H), 8.33 (d, J = 8.9 Hz, 2H), 8.16 (s, 1H), 8.04 (d, J = 8.8 Hz, 2H), 7.75 (d, J = 2.1 Hz, 1H), 7.49 (d, J = 3.7 Hz, 1H), 7.42 (d, J = 8.3 Hz, 1H), 7.28 (dd, J = 8.4, 2.0 Hz, 1H), 7.12 (d, J = 3.7 Hz, 1H).

**^13^C NMR (150 MHz, DMSO-d6) δ ppm:** 159.97 (C), 152.67 (C), 151.27 (C), 148.83 (C), 146.73 (C), 141.93 (C), 135.68 (C), 134.83 (CH), 125.82 (C), 125.03 (CH), 124.92 (CH), 117.90 (CH), 116.13 (CH), 113.02 (CH), 110.63 (CH).

**^13^C NMR CP-MAS (100 MHz, Probe: 4G Vr = 10 kHz) δ ppm:** 158.79, 150.53, 146.15, 142.75, 136.68, 133.28, 124.04, 121.37, 119.43, 112.14, 106.79.

**FTIR *v* cm^−1^:** 3136, 851 (=C-H), 1516 (HC=N), 1462 (C=C), 1329 (NO_2_), 1107 (=C-O-C=), 851 (C-Cl).

**MS (DCI-NH_3_)** *m/z*: 383.1 [M+H^+^].

**HRMS (ES, TOF, MeOH) *m/z:*** calc. for C_18_H_11_ClN_4_O_4_ **[M+H^+^] =** 383.0547; found: 383.0542.

N′-[(E)-[5-(4-nitrophenyl)furan-2-yl]methylidene](benzyloxy)carbohydrazide **17**. The compound was synthesized using general procedure A (0.78 mmol 5-(nitrophenyl)-furan-2carbaldehyde)—reaction time 3 × 30 min. Aldehyde conversion 40%. Crude product was purified with FCC using DCM:AcOEt 96:4 (35%). Procedure B (1.2 mmol each reactant, 6 × 30 min) afforded >98% aldehyde conversion, and after washing, compound **17** was obtained in 97% yield.

**m.p.:** 238.1–239.9 °C. **Rf:** 0.67 (DCM:AcOEt 96:4).

**^1^H NMR (600 MHz, DMSO) δ ppm:** 11.44 (s, 1H), 10.45 (s, 0.2H), 8.35–8.28 (m, 2.4H), 8.08–8.03 (m, 0.4H), 8.01 (d, J = 8.5 Hz, 2H), 7.98 (s, 1.2H), 7.51 (d, J = 3.7 Hz, 0.2H), 7.49–7.46 (m, 0.4H), 7.46–7.39 (m, 5.4H), 7.38–7.34 (m, 1.2H), 7.24 (d, J = 3.8 Hz, 0.2H), 7.04 (d, J = 3.7 Hz, 1H), 5.25 (s, 0.4H), 5.20 (s, 2H).

**^13^C NMR (150 MHz) δ ppm:** 154.56 (C=O), 153.55 (C=O), 152.66 (C), 152.48 (C), 151.43 (C), 148.37 (C), 147.11 (C), 146.70 (C), 136.93 (C), 136.88 (C), 135.71 (C), 135.31 (C), 134.32 (CH), 128.95 (CH), 128.92 (CH), 128.62 (CH), 128.57 (CH), 128.44 (CH), 125.42 (CH), 125.02 (CH), 124.98 (CH), 124.90 (CH), 118.76 (CH), 115.70 (CH), 112.87 (CH), 112.52 (CH), 66.86 (CH_2_), 66.61 (CH_2_).

**^13^C NMR CP-MAS (100 MHz, Probe: 4G Vr = 10 kHz) δ ppm:** 154.17, 151.50, 148.58, 147.85, 145.18, 143.97, 136.92, 135.71, 133.52, 131.87, 127.93, 125.99, 122.59, 117.97, 111.17, 110.44, 108.74, 67.43, 65.73.

**FTIR *v* cm^−1^:** 3054, 850 (=C-H), 1703 (C=O), 1515 (HC=N), 1462 (C=C), 1330 (NO_2_), 1238 (=C-O-C=).

**MS (DCI-NH_3_)** *m/z*: 366.1 [M+H^+^], 383.1 [M+NH_4_^+^].

**HRMS (ES, TOF, MeOH) *m/z:*** calc. for C_19_H_15_N_3_O_5_ **[M+H^+^] =** 366.1090; found: 366.1086.

## Figures and Tables

**Table 1:** Hydrazines used for the synthesis of hydrazones **1** and **2**, apparatus, time, degree of vanillin conversion, and yields of corresponding reactions.

Hydrazines (R^1^R^2^N–NH_2_)	MM400		P7	
	Time, Cycles × Min	Ald. Conversion (Yield), %	Time, Cycles × Min	Ald. Conversion (Yield), %
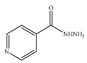	3 × 10	**1**:**1**′ = 90:10 70	2 × 30 **1**	>99 (99)
	2 × 30	**1**:**1**′ = 90:10 90		
	3 × 30	**1**:**1**′ = 90:10 >99 (99)		
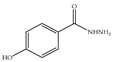	3 × 30	**2** >84	3 × 30 **2**	>99 (99)

**Table 2:** Hydrazines used for the synthesis of hydrazones, apparatus, time, degree of aldehyde conversion, and yields of corresponding reactions.

Hydrazines (R^1^R^2^N–NH_2_)	MM400		P7	
	Time, Cycles × Min	Ald. Conversion (Yield), %	Time, Cycles × Min	Ald. Conversion (Yield), %
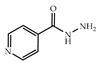	3 × 30	**3** 78 (70)	6 × 30	>99 (98)
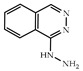	3 × 30	**4**^1^ >99 (99)	3 × 30	>99 (99)
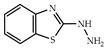	3 × 30	**5** >99 (99)	3 × 30	>99 (99)
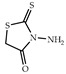	3 × 30	**6**:**6**′ = 90:10 75 (63)	6 × 30	>99 (97)
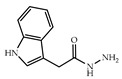	3 × 30	**7**:**7′** = 52:48 >99 (99)	6 × 30	>99 (99)

^1^ Synthesis preformed with 1 eq. of sodium acetate and the hydrazone was obtained after washing with brine and water to eliminate AcOH and NaCl that are formed.

**Table 3:** Hydrazines used for the synthesis of hydrazones, apparatus, time, degree of aldehyde conversion, and yields of corresponding reactions.

Hydrazines (R^1^R^2^N–NH_2_)	MM400		P7	
	Time, Cycles × Min	Ald. Conversion (Yield), %	Time, Cycles × Min	Ald. Conversion (Yield), %
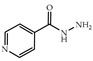	3 × 30	**8** >98 (98)	3 × 30	>99 (99)
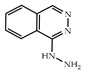	3 × 30	**9**^1^ 99 (85)	3 × 30	>99 (91)
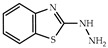	3 × 30	**10** 95 (84)	3 × 30	>99 (92)
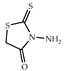	3 × 30	**11** 8	3 × 30	37
			6 × 30	97 (93)
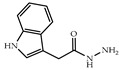	3 × 30	**12**:**12′** = 50:50 95 (70)	6 × 30	>99 (98)
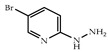	3 × 30	**13**:**13′** = 82:18 99 (95)	3 × 30	>99 (95)
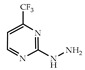	3 × 30	**14**:**14′** = 78:22 60 (47)	6 × 30	>99 (90)
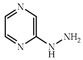	3 × 30	**15**:**15′** = 70:30 80 (60)	6 × 30	>97 (95)
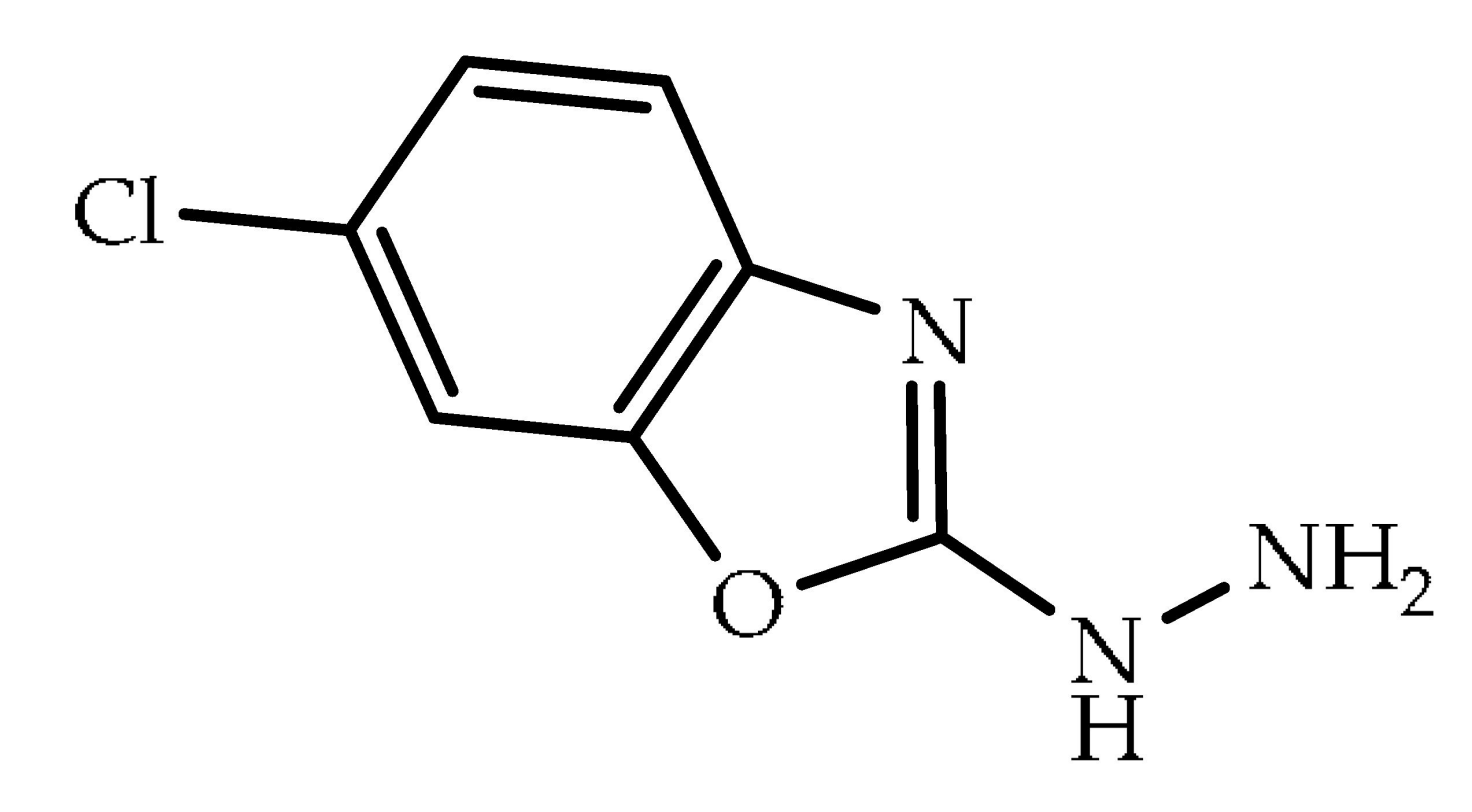	3 × 30	**16** 90 (54)	6 × 30	>99 (95)
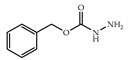	3 × 30	**17**:**17′** = 84:16 40 (35)	6 × 30	>98 (97)

^1^ Synthesis preformed with 1 eq. of sodium acetate and the hydrazone was obtained after washing with EtOH and water to eliminate AcOH and NaCl that are formed.

**Table 4:** Details of the refinement, crystal structures, and lattice parameters of compound **8**.

Formula, fw, g/mol	C_17_H_12_N_4_O_4,_ 336.31 g/mol				
temperature, K	100(2)	150(2)	200(2)	250(2)	300(2)
crystal size, mm	0.1 × 0.05 × 0.02	0.1 × 0.05 × 0.02	0.1 × 0.05 × 0.02	0.1 × 0.05× 0.02	0.1 × 0.05 × 0.02
crystal color	orange	orange	orange	orange	orange
crystal system	triclinic	triclinic	triclinic	triclinic	triclinic
space group, *Z*	*P*$\bar{1}$, 4	*P*$\bar{1}$, 4	*P*$\bar{1}$, 4	*P*$\bar{1}$, 4	*P*$\bar{1}$, 4
*a*, Å	8.6900(1)	8.7108(2)	8.7423(1)	8.7685(1)	8.7970(1)
*b*, Å	12.7764(1)	12.8057(2)	12.8394(1)	12.8688(1)	12.9010(2)
*c*, Å	14.5517(2)	14.5707(3)	14.5736(2)	14.5959(1)	14.6224(2)
*α*, °	94.261(1)	94.255(2)	94.210(1)	94.170(1)	108.031(2)
*β*, °	107.297(1)	107.188(2)	107.000(1)	106.818(1)	100.416(2)
*γ*, °	98.283(1)	98.309(2)	98.305(2)	98.309(1)	107.389(2)
V, Å^3^	1514.70(3)	1524.65(6)	1536.34(3)	1548.56(3)	1562.09(4)
*ρ*, g/cm^3^	1.475	1.465	1.454	1.460	1.447
*µ*, mm^–1^	0.910	0.904	0.897	0.890	0.883
F (000)	696	696	696	696	696
*θ* max, °	77.528	77.241	77.549	77.497	77.556
limiting indices	−10⇒h⇒8	−11⇒h⇒8	−11⇒h⇒8	−11⇒h⇒8	−11 ⇒ h⇒9
	−16⇒k⇒16	−16⇒k⇒16	−16⇒k⇒16	−16⇒k⇒16	−16⇒ k⇒16
	−16⇒l⇒18	−16⇒l⇒18	−16⇒l⇒18	−16⇒l⇒18	−16⇒l⇒18
reflns collected	6124	6151	6221	6260	6303
R_int_	0.0240	0.0238	0.0241	0.0237	0.0363
data/parameters	5467/451	5373/451	5295/451	5178/451	4974/451
GOF on F^2^	1.083	1.082	1.072	1.054	1.076
*R*_1_ [I > 2σ(I)]	0.0365	0.0369	0.0380	0.0381	0.0393
*R*_1_ (all data)	0.0411	0.0423	0.0452	0.0467	0.0537
*wR*_2_ (all data)	0.1005	0.1013	0.1026	0.1018	0.1031
completeness to theta max	95%	95.1%	95.1%	95%	94.7%
lrgst diff peak, e/Å^3^	0.23	0.20	0.21	0.20	0.17
lrgst diff hole, e/Å^3^	−0.31	−0.27	−0.30	−0.28	−0.24

**Table 5:** Short interactions and dihedral angles in crystals of **8** in the function of temperature.

T (K)	*π-π* Stacking (Centroid- Centroid Distance, Å)	*π-π* Stacking (Centroid- Centroid Angle, °)	N_8A_-O_25_ Hydrogen Bond Distance (Å)	H_8A_-O_25_ Hydrogen Bond Distance (Å)	N_8_-N_13_ Hydrogen Bond Distance (Å)	H_8_-N_13_ Hydrogen Bond Distance (Å)	Torsion Angles N_8_-C_9_-C_10_-C_15_ (°)
100	3.817	67.76	2.8661(14)	2.0374	2.9019(16)	2.1483	38.31(17)
150	3.835	68.15	2.8727(14)	2.0444	2.9169(16)	2.1596	38.19(17)
200	3.852	68.63	2.8781(14)	2.0507	2.9253(16)	2.1713	37.94(18)
250	3.874	69.1	2.8855(14)	2.0583	2.9333(16)	2.1818	37.74(17)
300	3.897	39.73	2.8935(15)	2.0657	2.9451(17)	2.1962	37.34(19)

**Table 6:** Biological evaluation of reported hydrazones.

Compound	*L. donovani* Axenic Amastigote	*L. donovani* Intramacrophage Amastigote	SI * (Selectivity Index)	*M. tuberculosis* H37Rv	*S. aureus* ATCC25923 and (ATCC29213)	*E. coli* ATCC25922
	IC_50_ ± SD (µM)	IC_50_ ± SD (µM)		MIC (µM)	MIC (µM)	MIC (µM)
**1**	24.9 ± 2.9	40.2 ± 3.1	>2.5	0.6	>944	>944
**2**	2.1 ± 0.1	5.9 ± 0.9	>17	>100	>894	>894
**3**	0.6 ± 0.1	0.5 ± 0.1	>200	1.0	1.75 (7)	28
**4**	1.9 ± 0.3	0.6 ± 0.1	>166	129	>828	>828
**5**	2.3 ± 0.3	12.4 ± 1.2	>8	1.0	>814	>814
**6**	0.7 ± 0.1	0.8 ± 0.1	>125	4.2	13 (7)	>861
**7**	0.4 ± 0.1	0.8 ± 0.2	>142	7.4	0.7 (3)	>757 (47)
**8**	16.2 ± 1.2	36.2 ± 1.4	>2	0.9	>761	>761
**9**	0.2 ± 0.1	0.3 ± 0.1	>333	>100	>711	>711
**10**	0.7 ± 0.1	5.8 ± 1.0	>2	>100	>703	>703
**11**	5.2 ± 0.3	>100	ND	>100	>737	>737
**12**	2.7 ± 0.7	20.5 ± 2.1	>5	52	>659	>659
**13**	54.3 ± 4.5	>100	ND	>100	>661	>661
**14**	2.9 ± 0.2	11.9 ± 1.1	>8	>100	>669	>669
**15**	9.9 ± 0.8	23.3 ± 3.1	>4	>100	>678	>678
**16**	>100	>100	ND	>100	>828	>828
**17**	12.2 ± 1.6	32.4 ± 2.8	>3	>100	>701	>701
Reference	1.5 ± 0.3 ^a^	2.2 ± 0.3	9 ^a^	0.4 ^b^; 0.4 ^c^	3 (5) ^d^	0.75 ^d^

* Selectivity index (SI) corresponds to CC_50_/IC_50_, CC50 being defined for macrophage RAW 264.7 and IC_50_ being defined for the intramacrophage form of L. donovani; ^a^ Miltefosine, ^b^ Isoniazid, ^c^ Streptomycin, ^d^ Ciprofloxacin.

## Data Availability

The crystallographic data are deposited in the Cambridge Crystallographic Data Base (Refs. CCDC 2258486-2258490). All other data are present in the experimental section and the supplementary material.

## Supplementary Materials

The following supporting information can be downloaded at: https://www.mdpi.com/article/10.3390/molecules28135284/s1, All recorded NMR spectra on Figures S1–S77; Table S1: ^1^H NMR Spectroscopic Data of 6−10 in DMSO-d6 (δ in ppm, J in Hz) and Table S2: ^13^C NMR Spectroscopic Data of 6−10 in DMSO-d6 (δ in ppm). Crystal structures can be found in the Cambridge Crystallographic Data Base (Refs. CCDC 2258486-2258490).

## Sample Availability

Samples of all compounds are available from the authors.

## References

[1] Houngue M.T.A.K., N’bouke M., Atchade B., Doco R.C., Kuevi U.A., Kpotin G.A., Kpoviessi S.D.S., Atohoun Y.G.S., Badawi M., Mensah J.-B. (2018). Quantum Chemical Studies of Some Hydrazone Derivatives. Comput. Chem..

[2] Salah B.A., Kandil A.T., Abd El-Nasser M.G. (2019). Synthesis, Characterization, Computational and Biological Activity of Novel Hydrazone Complexes. J. Radiat. Res. Appl. Sci..

[3] Ali A., Khalid M., Rehman M.A., Anwar F., Zain-Ul-Aabidin H., Akhtar M.N., Khan M.U., Braga A.A.C., Assiri M.A., Imran M. (2020). An Experimental and Computational Exploration on the Electronic, Spectroscopic, and Reactivity Properties of Novel Halo-Functionalized Hydrazones. ACS Omega.

[4] Palanimuthu D., Wu Z., Jansson P.J., Braidy N., Bernhardt P.V., Richardson D.R., Kalinowski D.S. (2018). Novel Chelators Based on Adamantane-Derived Semicarbazones and Hydrazones That Target Multiple Hallmarks of Alzheimer’s Disease. Dalton Trans..

[5] Anjum R., Palanimuthu D., Kalinowski D.S., Lewis W., Park K.C., Kovacevic Z., Khan I.U., Richardson D.R. (2019). Synthesis, Characterization, and in Vitro Anticancer Activity of Copper and Zinc Bis(Thiosemicarbazone) Complexes. Inorg. Chem..

[6] Liu R., Cui J., Ding T., Liu Y., Liang H. (2022). Research Progress on the Biological Activities of Metal Complexes Bearing Polycyclic Aromatic Hydrazones. Molecules.

[7] Popiołek Ł. (2017). Hydrazide–Hydrazones as Potential Antimicrobial Agents: Overview of the Literature since 2010. Med. Chem. Res..

[8] Wahbeh J., Milkowski S. (2019). The Use of Hydrazones for Biomedical Applications. SLAS Technol..

[9] Pahlavani E., Kargar H., Rad N.P. (2015). A Study on Antitubercular and Antimicrobial Activity of Isoniazid Derivative. Zahedan J. Res. Med. Sci..

[10] Gil-Longo J., Laguna M.D.L.R., Verde I., Castro M.E., Orallo F., Fontenla J.A., Calleja J.M., Ravina E., Teran C. (1993). Pyridazine Derivatives. XI: Antihypertensive Activity of 3-Hydrazinocycloheptyl[1,2-c]Pyridazine and Its Hydrazone Derivatives. J. Pharm. Sci..

[11] Sharma P.C., Sharma D., Sharma A., Saini N., Goyal R., Ola M., Chawla R., Thakur V.K. (2020). Hydrazone Comprising Compounds as Promising Anti-Infective Agents: Chemistry and Structure-Property Relationship. Mater. Today Chem..

[12] Kumar P., Kadyan K., Duhan M., Sindhu J., Singh V., Saharan B.S. (2017). Design, Synthesis, Conformational and Molecular Docking Study of Some Novel Acyl Hydrazone Based Molecular Hybrids as Antimalarial and Antimicrobial Agents. Chem. Cent. J..

[13] Vargas E., Echeverri F., Upegui Y., Robledo S., Quiñones W. (2017). Hydrazone Derivatives Enhance Antileishmanial Activity of Thiochroman-4-Ones. Molecules.

[14] Ali R., Marella A., Alam T., Naz R., Saha R., Tanwar O., Hooda J. (2012). Review of Biological Activities of Hydrazones. Indones. J. Pharm..

[15] Potůčková E., Hrušková K., Bureš J., Kovaříková P., Špirková I.A., Pravdíková K., Kolbabová L., Hergeselová T., Hašková P., Jansová H. (2014). Structure-Activity Relationships of Novel Salicylaldehyde Isonicotinoyl Hydrazone (SIH) Analogs: Iron Chelation, Anti-Oxidant and Cytotoxic Properties. PLoS ONE.

[16] Świątek P., Saczko J., Rembiałkowska N., Kulbacka J. (2019). Synthesis of New Hydrazone Derivatives and Evaluation of Their Efficacy as Proliferation Inhibitors in Human Cancer Cells. Med. Chem..

[17] Amato J., Miglietta G., Morigi R., Iaccarino N., Locatelli A., Leoni A., Novellino E., Pagano B., Capranico G., Randazzo A. (2020). Monohydrazone Based G-Quadruplex Selective Ligands Induce DNA Damage and Genome Instability in Human Cancer Cells. J. Med. Chem..

[18] Amariucai-Mantu D., Mangalagiu V., Bejan I., Aricu A., Mangalagiu I.I. (2022). Hybrid azine derivatives: A useful approach for antimicrobial therapy. Pharmaceutics.

[19] Younis A., Awad G. (2019). Utilization of Ultrasonic as an Approach of Green Chemistry for Synthesis of Hydrazones and Bishydrazones as Potential Antimicrobial Agents. Egypt. J. Chem..

[20] Ješelnik M., Varma R.S., Polanc S., Kočevar M. (2002). Solid-State Synthesis of Heterocyclic Hydrazones Using Microwaves under Catalyst-Free ConditionsPresented, in Part, at the 5th Electronic Conference on Synthetic Organic Chemistry, (ECSOC-5), 1–30 September 2001, E0014, Http://Www.Mdpi.Org/Ecsoc-5.Htm. Green Chem..

[21] Hajipour A.R., Mohammadpoor-Baltork I., Bigdeli M. (1999). A Convenient and Mild Procedure for the Synthesis of Hydrazones and Semicarbazones from Aldehydes or Ketones under Solvent-Free Conditions. J. Chem. Res..

[22] Kaupp G., Schmeyers J., Boy J. (2000). Iminium Salts in Solid-State Syntheses Giving 100% Yield. J. Für Prakt. Chem..

[23] Nun P., Martin C., Martinez J., Lamaty F. (2011). Solvent-Free Synthesis of Hydrazones and Their Subsequent N-Alkylation in a Ball-Mill. Tetrahedron.

[24] Colacino E., Porcheddu A., Halasz I., Charnay C., Delogu F., Guerra R., Fullenwarth J. (2018). Mechanochemistry for “No Solvent, No Base” Preparation of Hydantoin-Based Active Pharmaceutical Ingredients: Nitrofurantoin and Dantrolene. Green Chem..

[25] Oliveira P.F.M., Baron M., Chamayou A., André-Barrès C., Guidetti B., Baltas M. (2014). Solvent-Free Mechanochemical Route for Green Synthesis of Pharmaceutically Attractive Phenol-Hydrazones. RSC Adv..

[26] Oliveira P., Guidetti B., Chamayou A., André-Barrès C., Madacki J., Korduláková J., Mori G., Orena B., Chiarelli L., Pasca M. (2017). Mechanochemical Synthesis and Biological Evaluation of Novel Isoniazid Derivatives with Potent Antitubercular Activity. Molecules.

[27] Gonnet L., André-Barrès C., Guidetti B., Chamayou A., Menendez C., Baron M., Calvet R., Baltas M. (2020). Study of the Two Steps and One-Pot Two-Step Mechanochemical Synthesis of Annulated 1,2,4-Triazoles. ACS Sustain. Chem. Eng..

[28] Verbitskiy E.V., Rusinov G.L., Charushin V.N., Chupakhin O.N. (2019). Development of new antituberculosis drugs among of 1,3- and 1,4-diazines. Highlights and perspectives. Russ. Chem. Bull. Int. Ed..

[29] Elsaman T., Mohamed M.S., Mohamed M.A. (2019). Current Development of 5-Nitrofuran-2-yl Derivatives as Antitubercular Agents. Bioorganic Chem..

[30] Le V.V.H., Rakonjac J. (2021). Nitrofurans: Revival of an “Old” Drug Class in the Fight against Antibiotic Resistance. PLOS Pathog..

[31] Kargar H., Moghimi A., Fallah-Mehrjardi M., Behjatmanesh-Ardakani R., Rudbari H.A., Munawar K.S. (2022). New Oxovanadium and Dioxomolybdenum Complexes as Catalysts for Sulfoxidation: Experimental and Theoretical Investigations of E and Z Isomers of ONO Tridentate Schiff Base Ligand. J. Sulfur. Chem..

[32] Huskić I., Lennox C.B., Friščić T. (2020). Accelerated Ageing Reactions: Towards Simpler, Solvent-Free, Low Energy Chemistry. Green Chem..

[33] Aguirre G., Boiani L., Cerecetto H., Fernández M., González M., Denicola A., Otero L., Gambino D., Rigol C., Olea-Azar C. (2004). In Vitro Activity and Mechanism of Action against the Protozoan Parasite Trypanosoma Cruzi of 5-Nitrofuryl Containing Thiosemicarbazones. Bioorg. Med. Chem..

[34] Vieites M., Otero L., Santos D., Olea-Azar C., Norambuena E., Aguirre G., Cerecetto H., González M., Kemmerling U., Morello A. (2009). Platinum-Based Complexes of Bioactive 3-(5-Nitrofuryl)Acroleine Thiosemicarbazones Showing Anti-Trypanosoma Cruzi Activity. J. Inorg. Biochem..

[35] Foscolos A.-S., Papanastasiou I., Foscolos G.B., Tsotinis A., Kellici T.F., Mavromoustakos T., Taylor M.C., Kelly J.M. (2016). New Hydrazones of 5-Nitro-2-Furaldehyde with Adamantanealkanohydrazides: Synthesis and in Vitro Trypanocidal Activity. MedChemComm.

[36] Harwood L.M., Claridge T.D.W. (2011). Introduction to Organic Spectroscopy.

[37] Nikitin K., O’Gara R. (2019). Mechanisms and Beyond: Elucidation of Fluxional Dynamics by Exchange NMR Spectroscopy. Chem.–Eur. J..

[38] Crawford D.E., Porcheddu A., McCalmont A.S., Delogu F., James S.L., Colacino E. (2020). Solvent-Free, Continuous Synthesis of Hydrazone-Based Active Pharmaceutical Ingredients by Twin-Screw Extrusion. ACS Sustain. Chem. Eng..

[39] Sović I., Lukin S., Meštrović E., Halasz I., Porcheddu A., Delogu F., Ricci P.C., Caron F., Perilli T., Dogan A. (2020). Mechanochemical Preparation of Active Pharmaceutical Ingredients Monitored by In Situ Raman Spectroscopy. ACS Omega.

[40] Bolognino I., Giangregorio N., Tonazzi A., Martínez A.L., Altomare C.D., Loza M.I., Sablone S., Cellamare S., Catto M. (2021). Synthesis and Biological Evaluation of Dantrolene-Like Hydrazide and Hydrazone Analogues as Multitarget Agents for Neurodegenerative Diseases. ChemMedChem.

[41] (2010). CrysAlisPro.

[42] Sheldrick G.M. (2008). A Short History of *SHELX*. Acta Crystallogr. A.

[43] Pomel S., Cojean S., Pons V., Cintrat J.-C., Nguyen L., Vacus J., Pruvost A., Barbier J., Gillet D., Loiseau P.M. (2021). An Adamantamine Derivative as a Drug Candidate for the Treatment of Visceral Leishmaniasis. J. Antimicrob. Chemother..

[44] Palomino J.-C., Martin A., Camacho M., Guerra H., Swings J., Portaels F. (2002). Resazurin Microtiter Assay Plate: Simple and Inexpensive Method for Detection of Drug Resistance in *Mycobacterium Tuberculosis*. Antimicrob. Agents Chemother..

[45] Martin A. (2006). A New Rapid and Simple Colorimetric Method to Detect Pyrazinamide Resistance in Mycobacterium Tuberculosis Using Nicotinamide. J. Antimicrob. Chemother..

[46] CLSI (2011). CLSI Document M100-S21.

