# Supplementary material for: Mechanochemical Studies on Coupling of Hydrazines and Hydrazine Amides with Phenolic and Furanyl Aldehydes—Hydrazones with Antileishmanial and Antibacterial Activities
Source: Molecules. 2023 Jul 7;28(13):5284. doi: 10.3390/molecules28135284 (PMC10343660; doi:10.3390/molecules28135284)
Supplement: Supplementary file 1 [file molecules-28-05284-s001.zip › molecules-2466658-supplementary.pdf]

Supplementary Materials

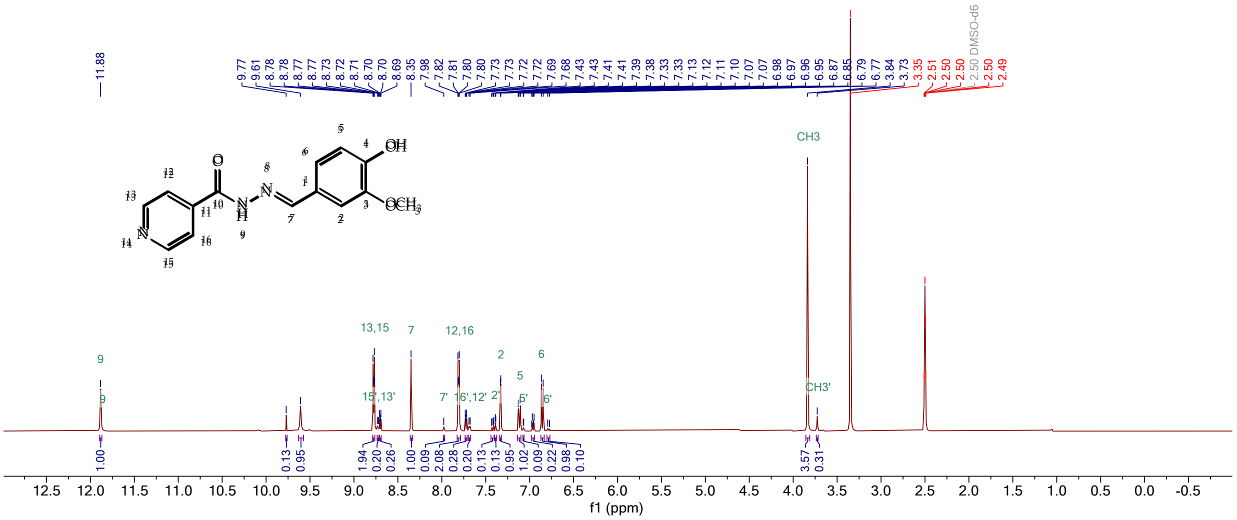

Figure S1: ^{1}H NMR (400 MHz, DMSO-d6, 298 K) of compound **1**

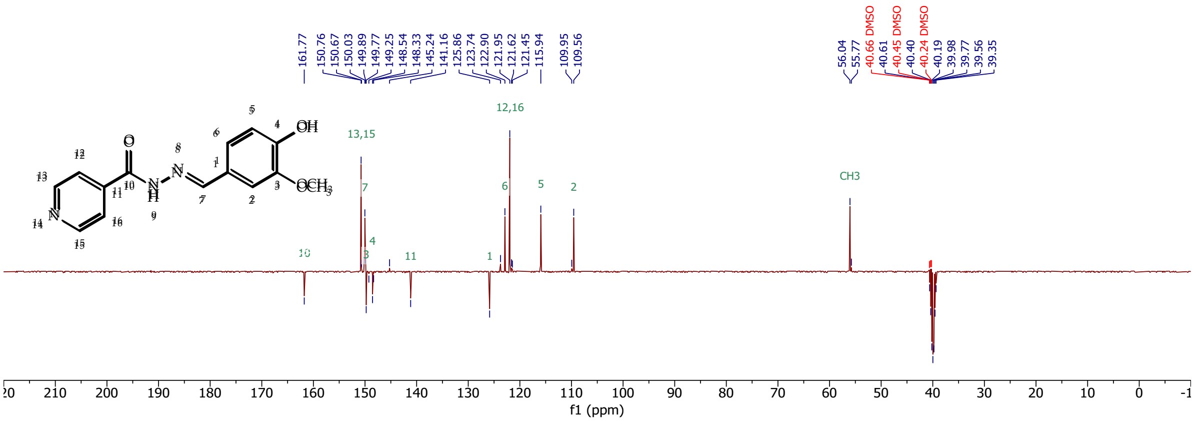

Figure S2: ^{13}C NMR (100 MHz, DMSO-d6, 298 K) of compound **1**

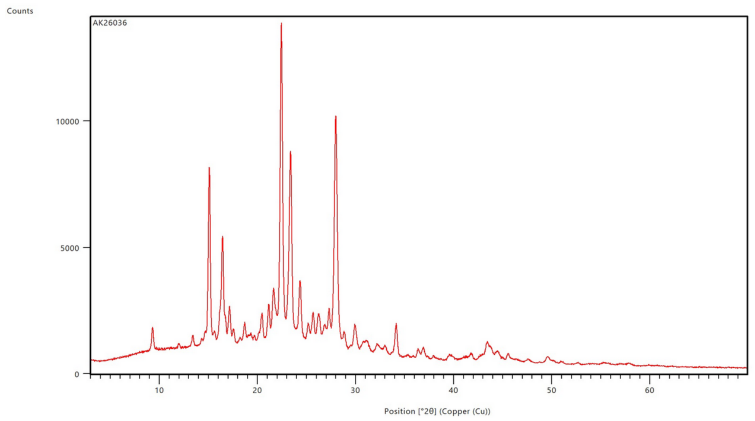

Figure S3: Powder XRD of compound **1**

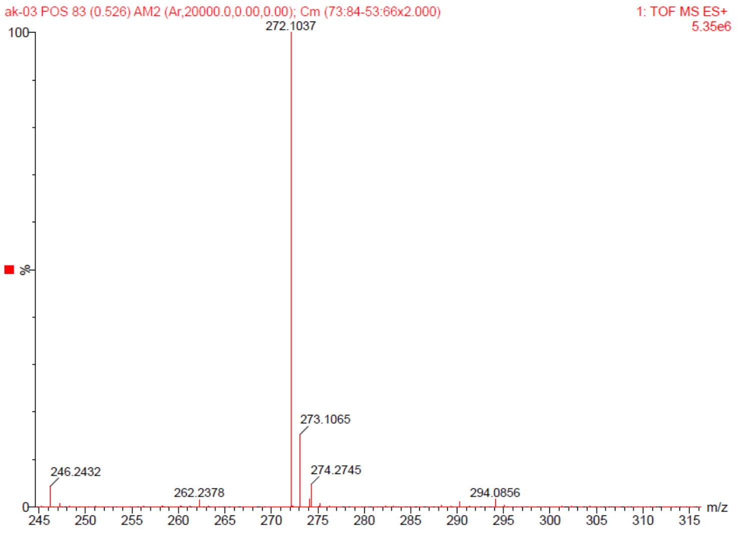

Figure S4: HRMS of compound **1**

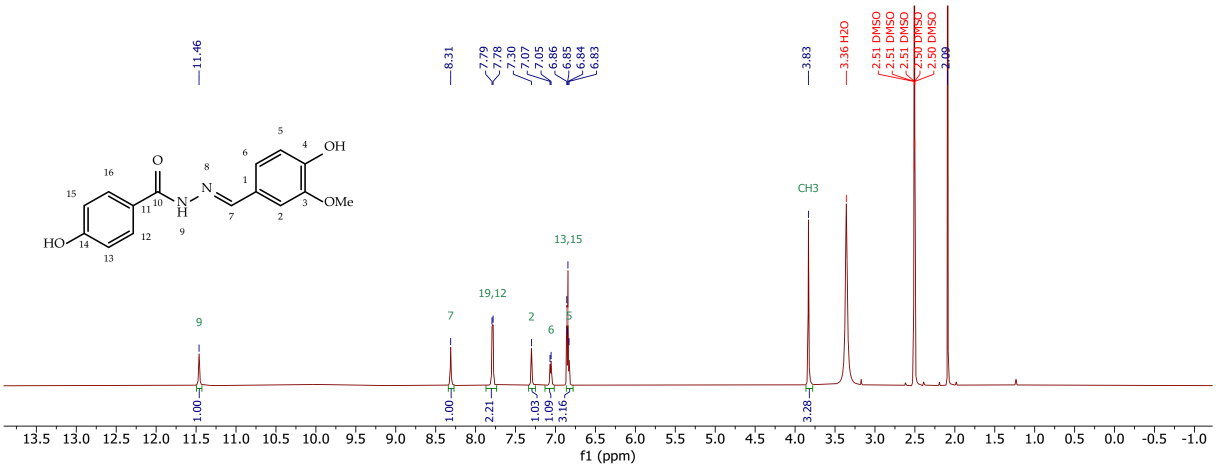

Figure S5: ^{1}H NMR (600 MHz, DMSO-d6, 298 K) of compound **2**

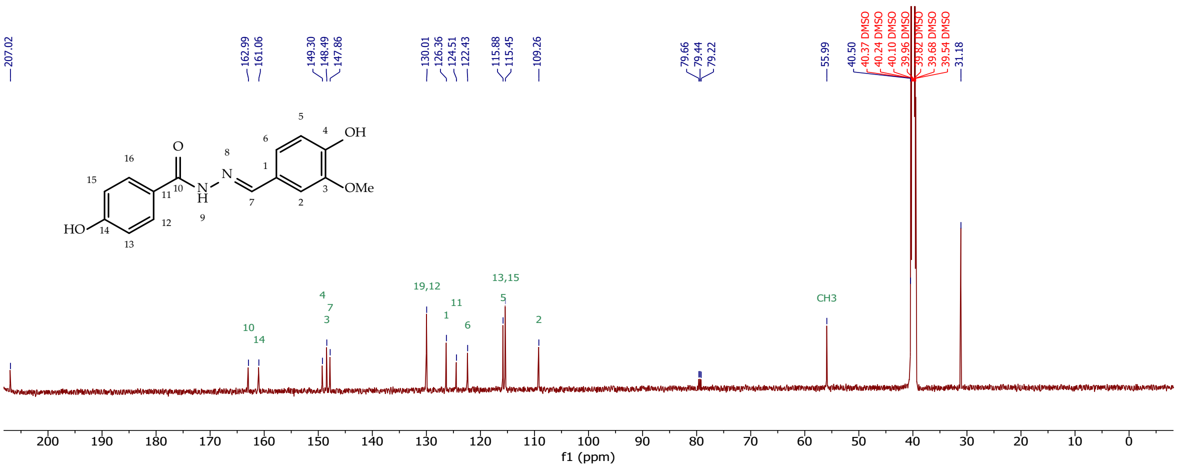

Figure S6: ^{13}C NMR (150 MHz, DMSO-d6, 298 K) of compound **2**

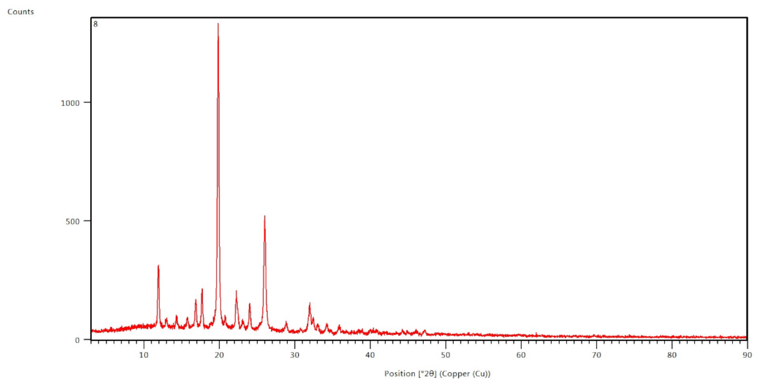

Figure S7: Powder XRD of compound **2**

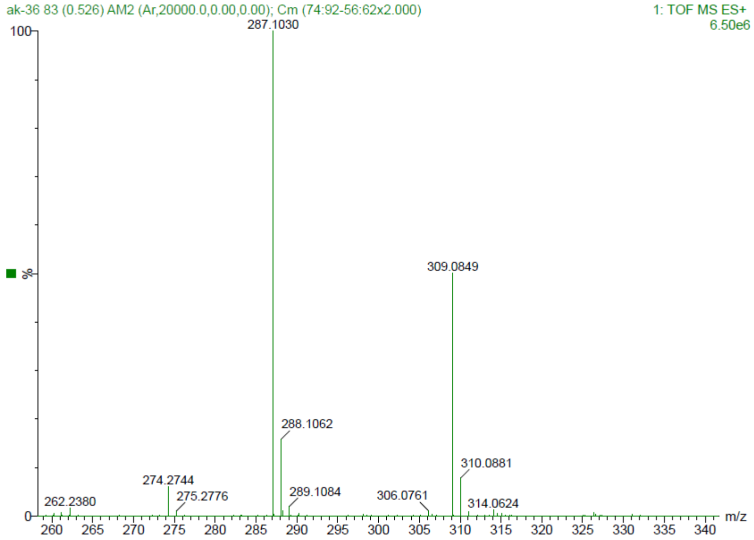

Figure S8: HRMS of compound **2**

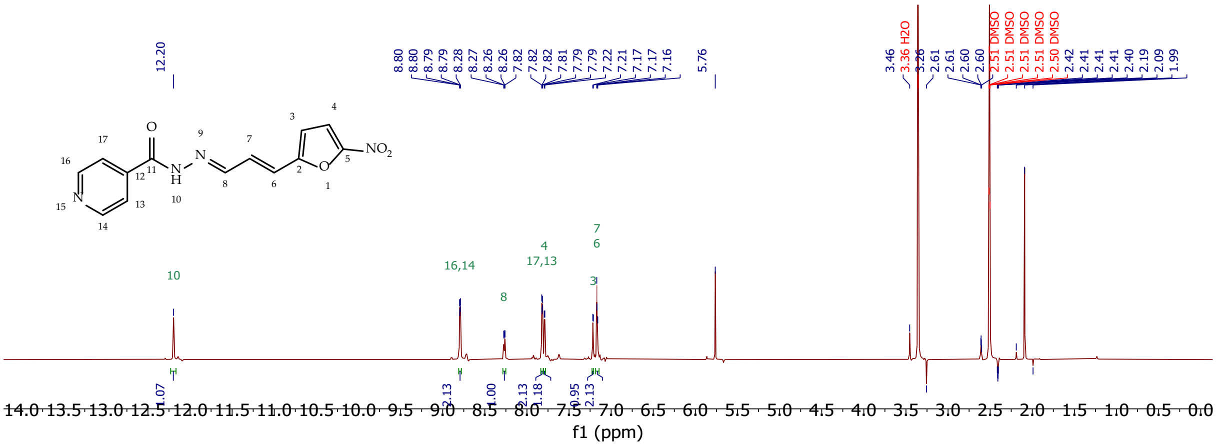

Figure S9: ^{1}H NMR (600 MHz, DMSO-d6, 298 K) of compound **3**

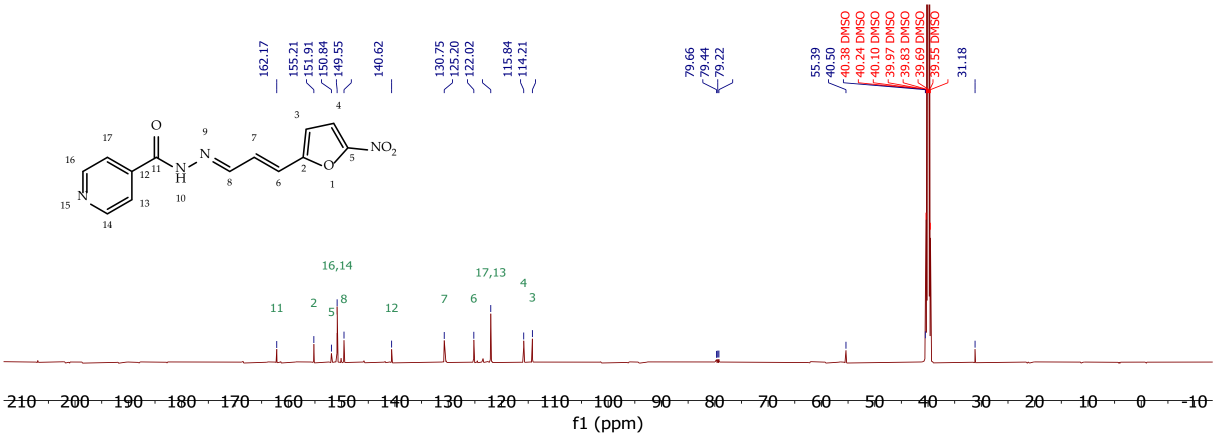

Figure S10: ^{13}C NMR (150 MHz, DMSO-d6, 298 K) of compound **3**

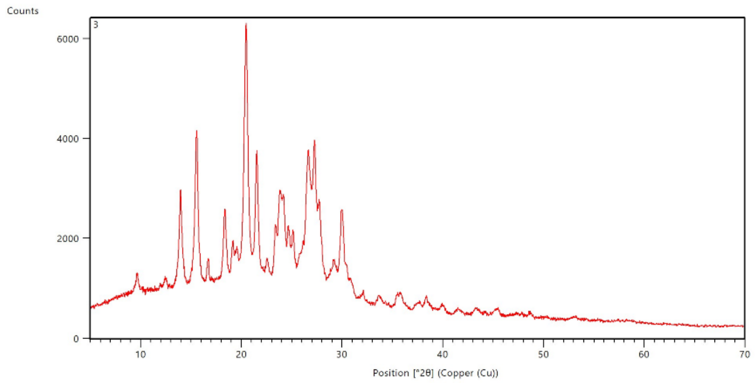

Figure S11: Powder XRD of compound **3**

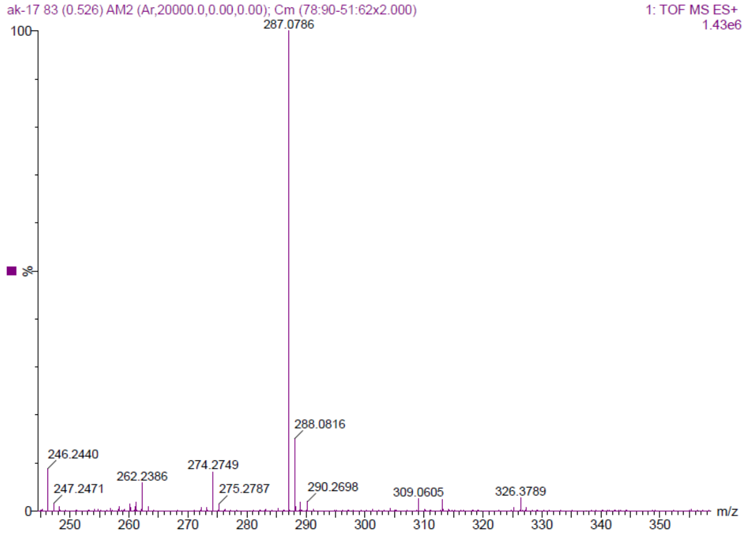

Figure S12: HRMS of compound **3**

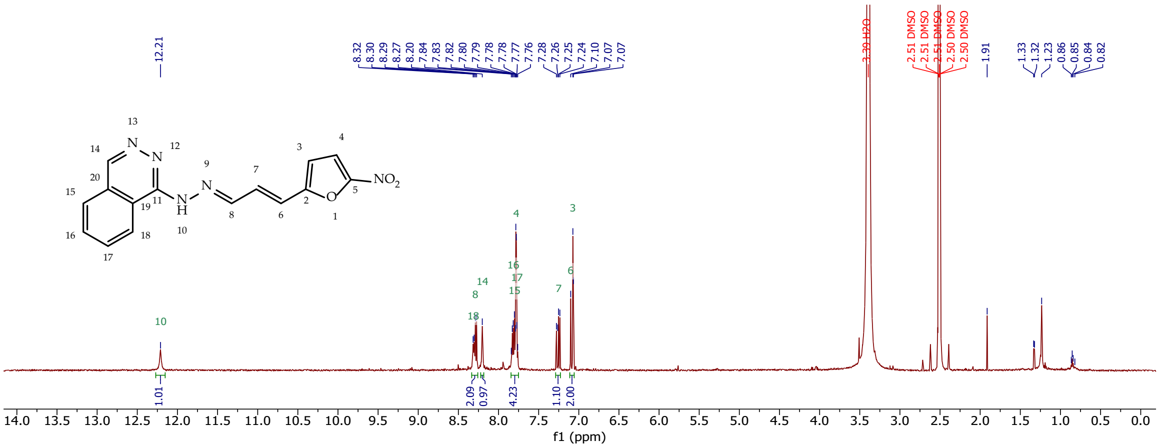

Figure S13: ¹H NMR (600 MHz, DMSO-d6, 298 K) of compound **4**

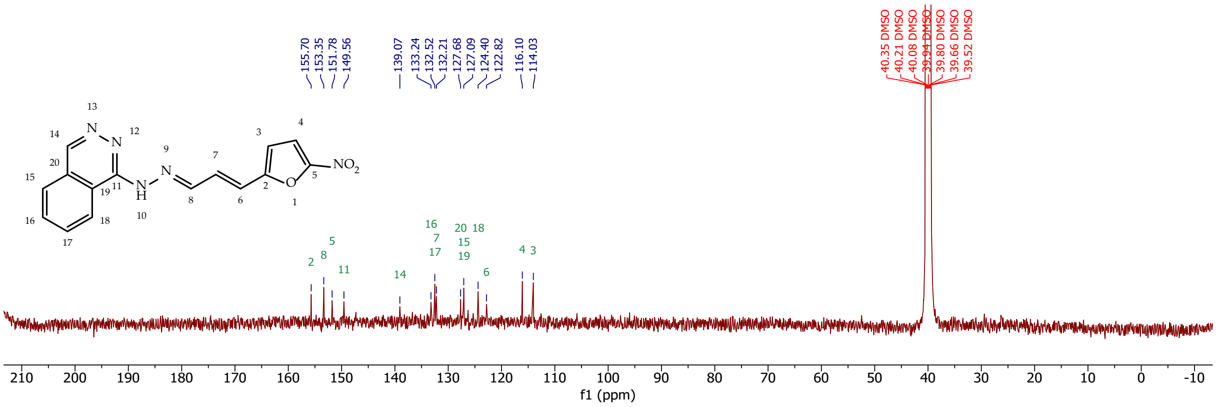

Figure S14: ¹³C NMR (150 MHz, DMSO-d6, 298 K) of compound **4**

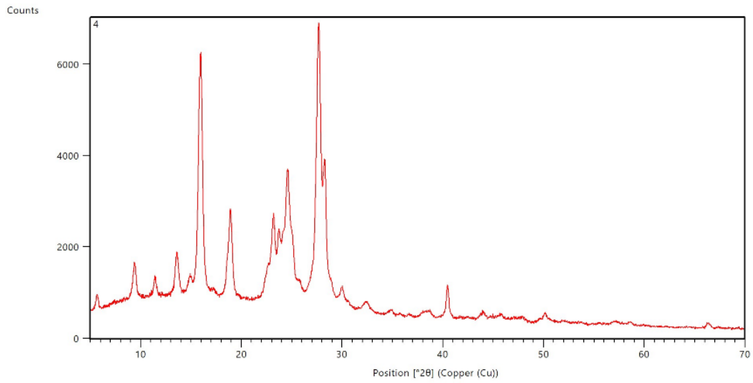

Figure S15: Powder XRD of compound **4**

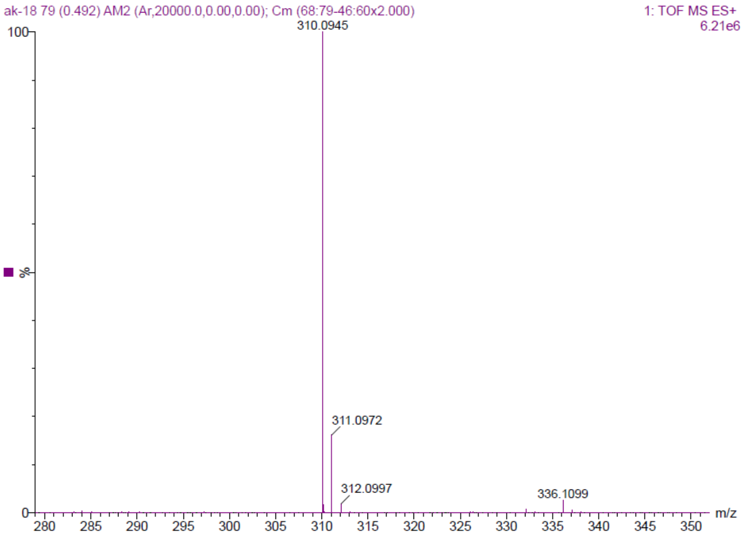

Figure S16: HRMS of compound **4**

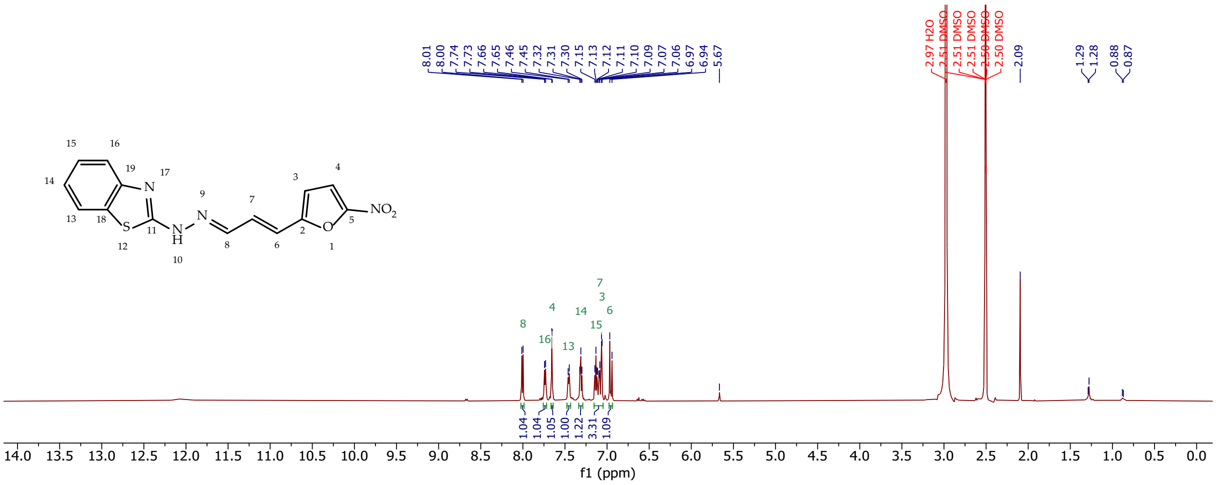

Figure S17: ^{1}H NMR (600 MHz, DMSO-d6, 378 K) of compound **5**

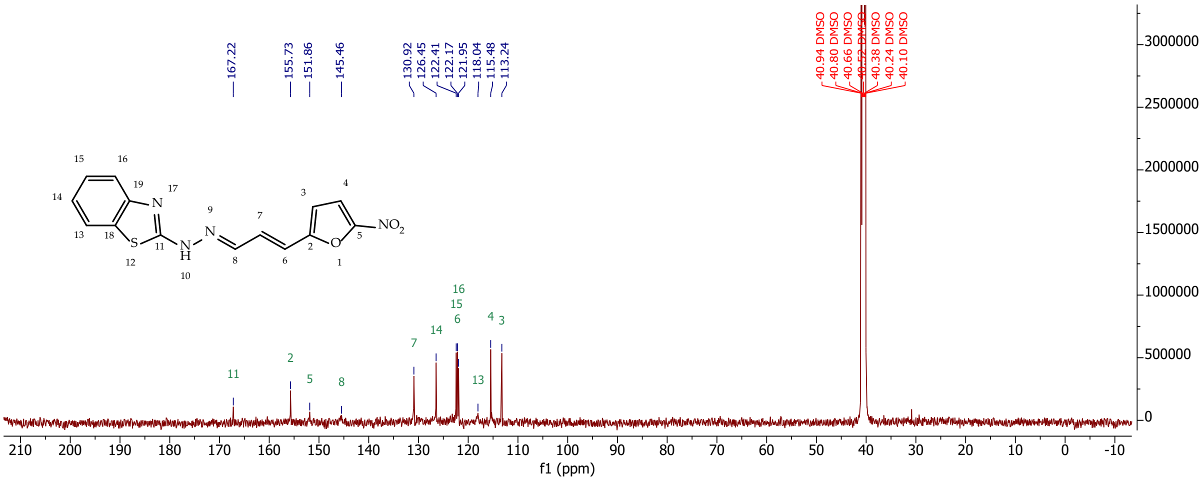

Figure S18: ^{13}C NMR (150 MHz, DMSO-d6, 378 K) of compound **5**

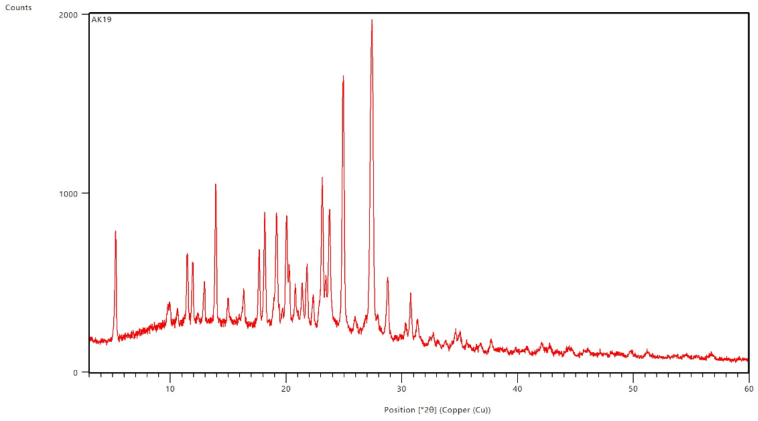

Figure S19: Powder XRD of compound **5**

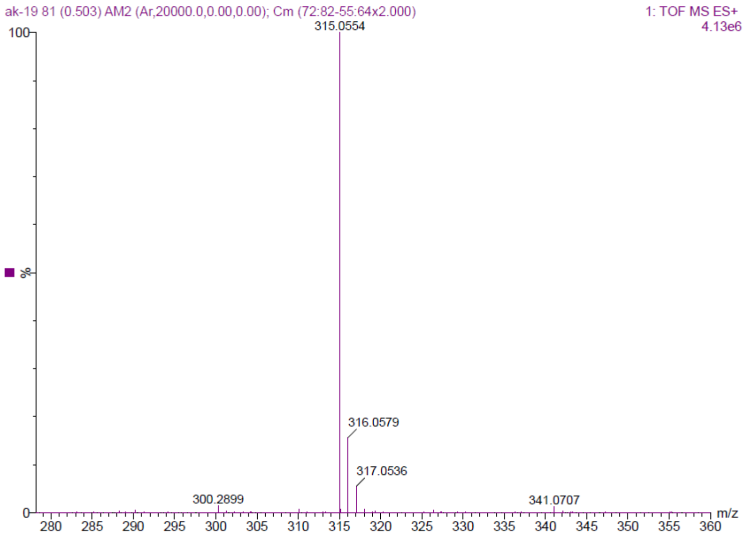

Figure S20: HRMS of compound **5**

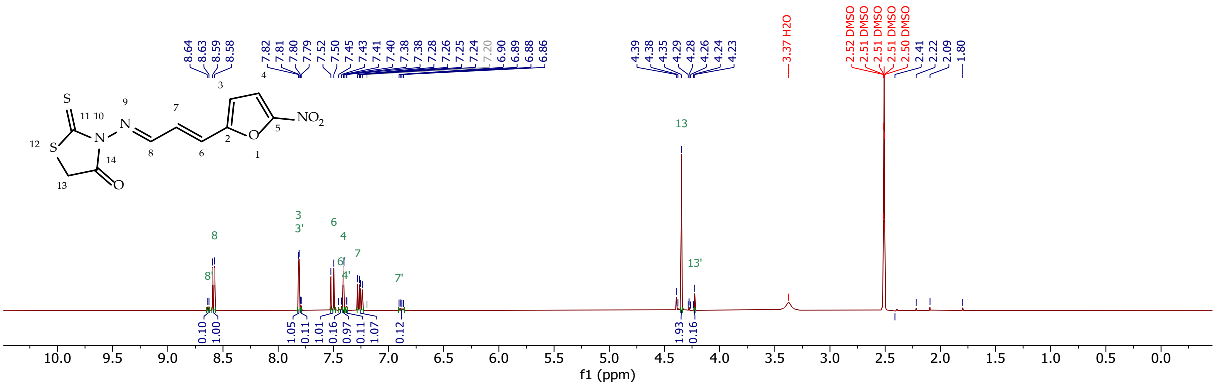

Figure S21: ¹H NMR (600 MHz, DMSO-d6, 298 K) of compound **6**

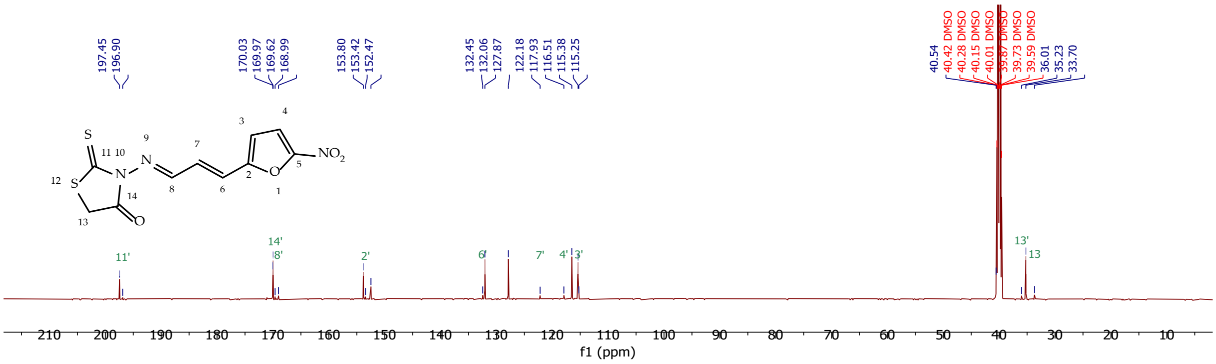

Figure S22: ^{13}C NMR (150 MHz, DMSO-d6, 298 K) of compound **6**

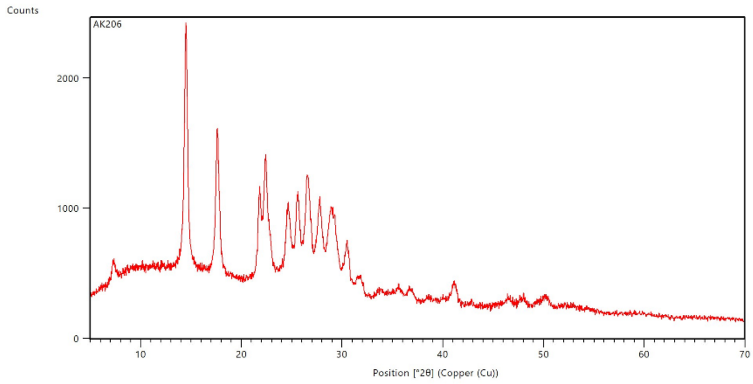

Figure S23: Powder XRD of compound **6**

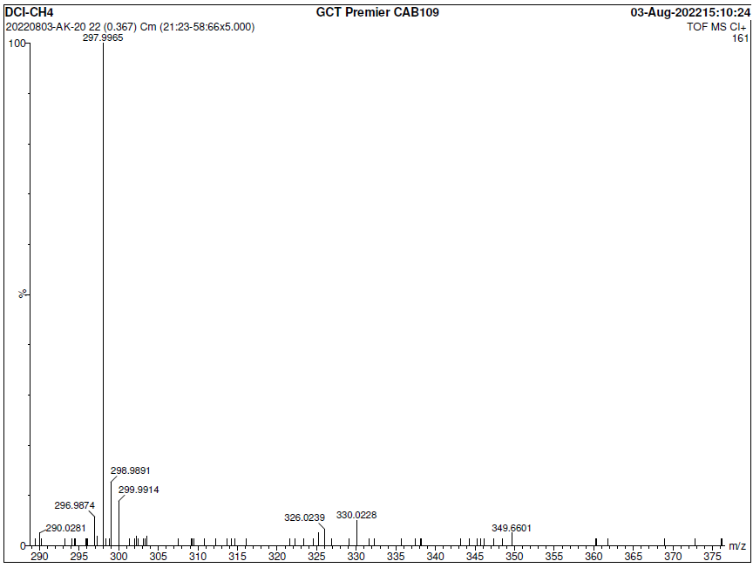

Figure S24: HRMS of compound **6**

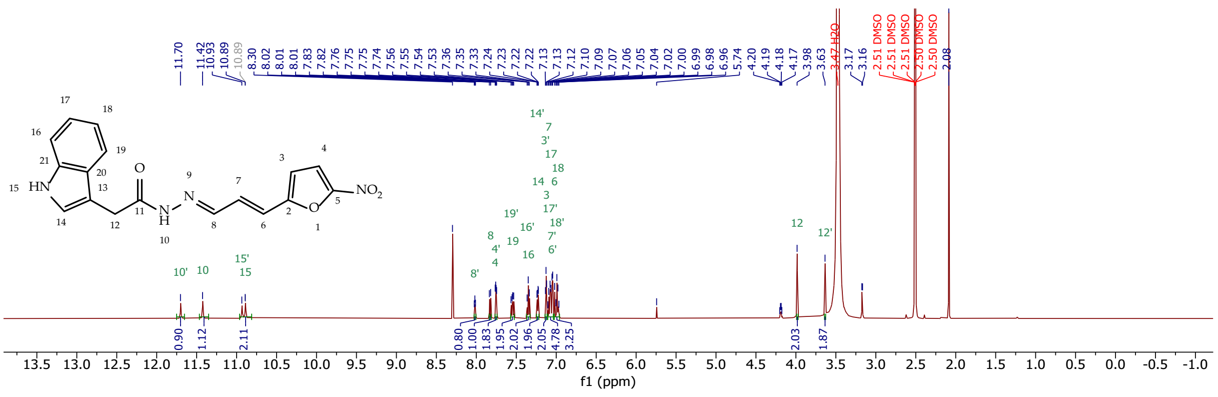

Figure S25: ^{1}H NMR (600 MHz, DMSO-d6, 298 K) of compound **7**

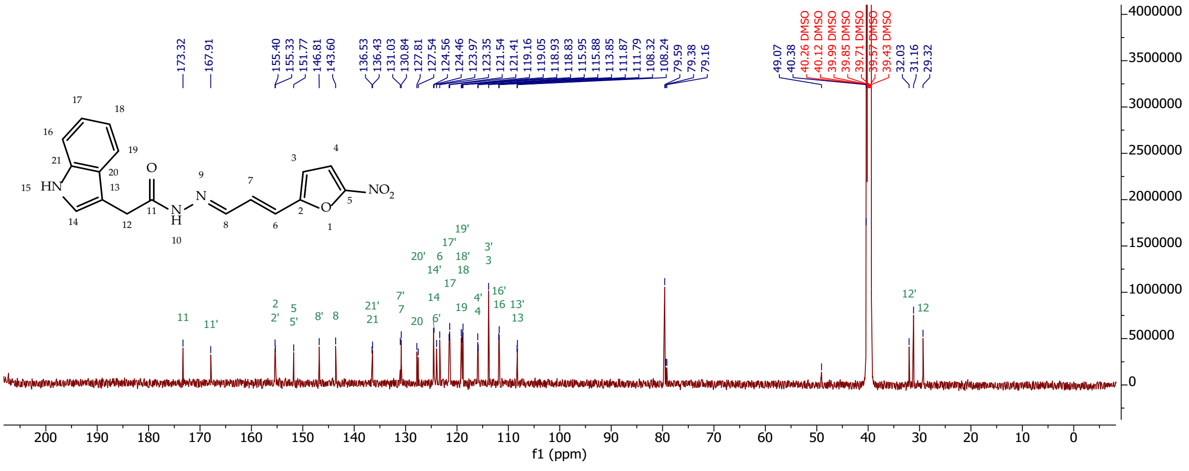

Figure S26: ^{13}C NMR (150 MHz, DMSO-d6, 298 K) of compound **7**

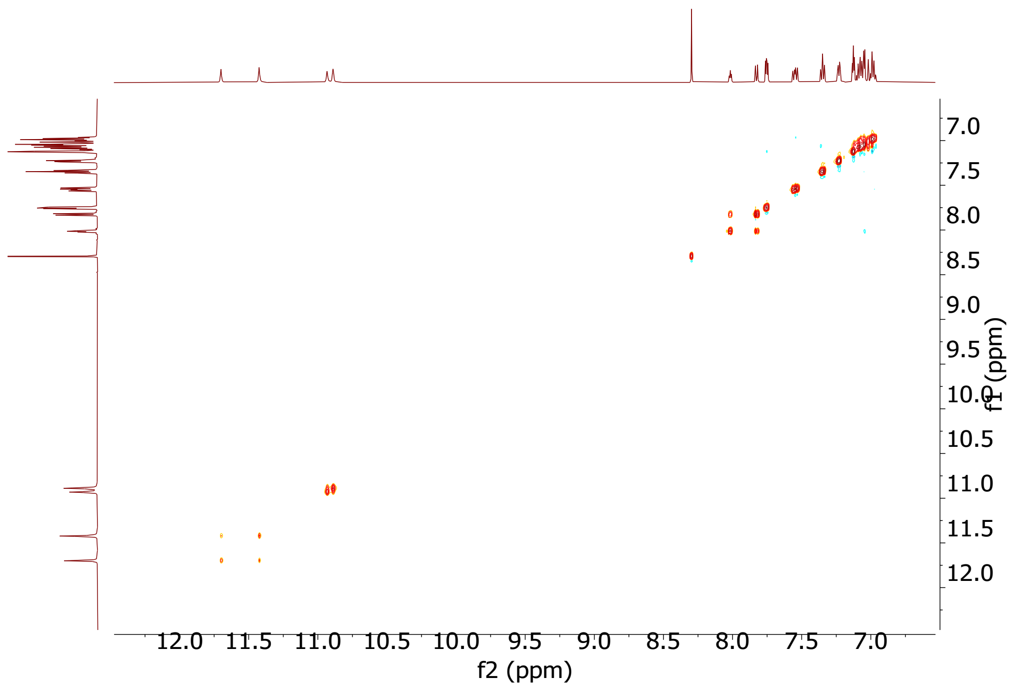

Figure S27: ROESY NMR (DMSO-d6, 298 K) of compound **7**

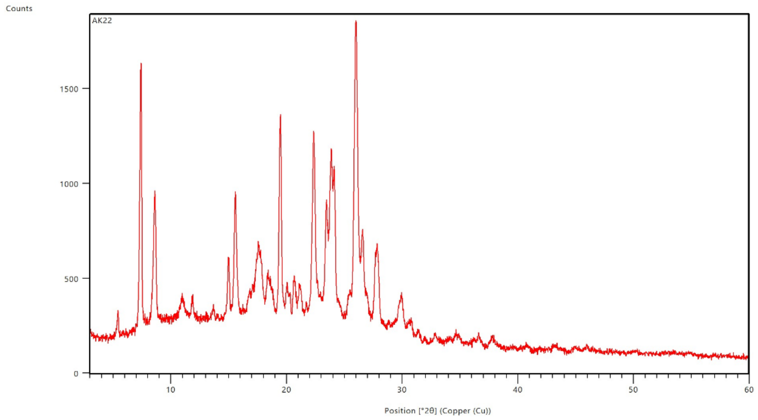

Figure S28: Powder XRD of compound **7**

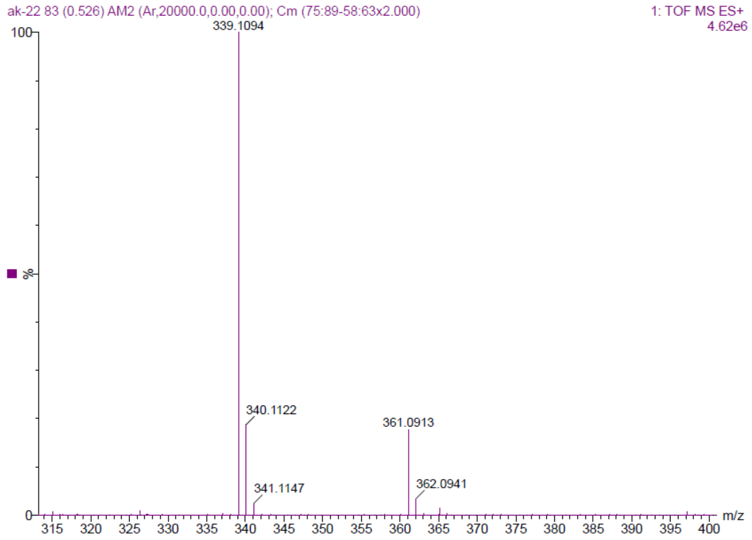

Figure S29: HRMS of compound **7**

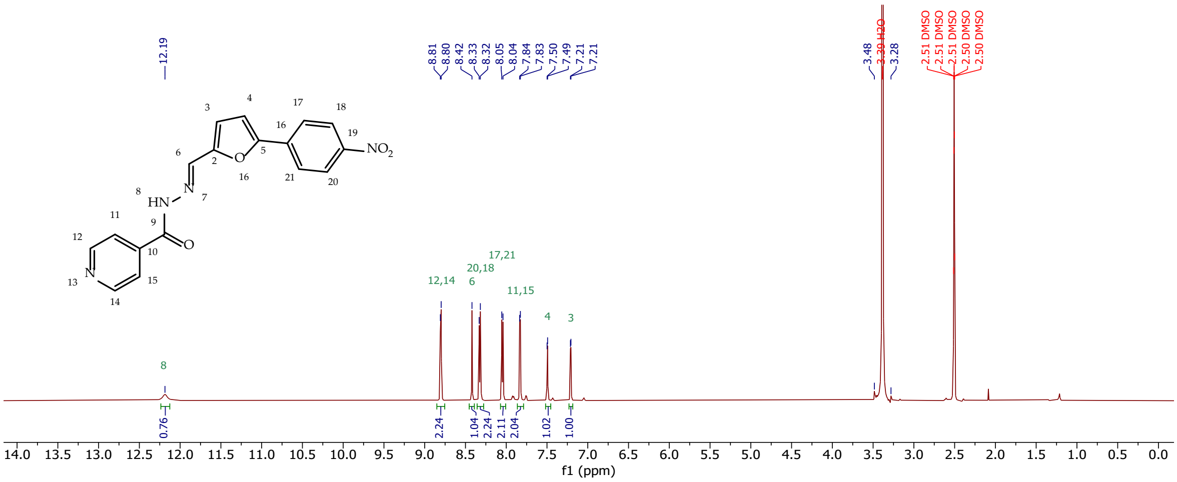

Figure S30: ^{1}H NMR (600 MHz, DMSO-d6, 298 K) of compound **8**

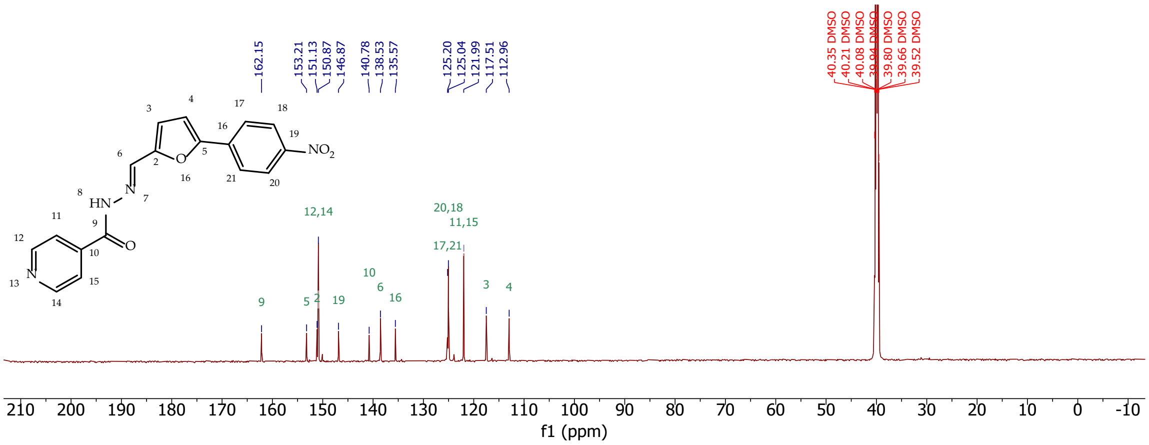

Figure S31: ^{13}C NMR (150 MHz, DMSO-d6, 298 K) of compound **8**

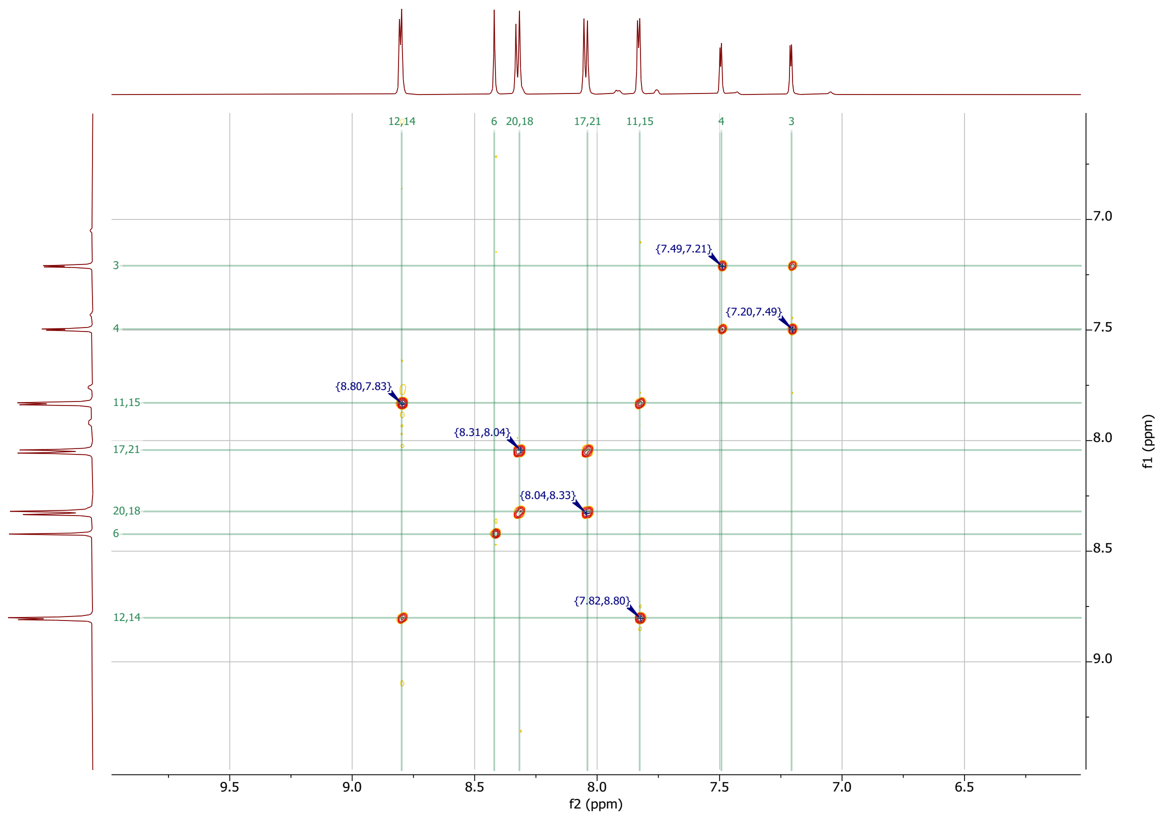

Figure S32: COSY NMR (DMSO-d6, 298 K) of compound **8**

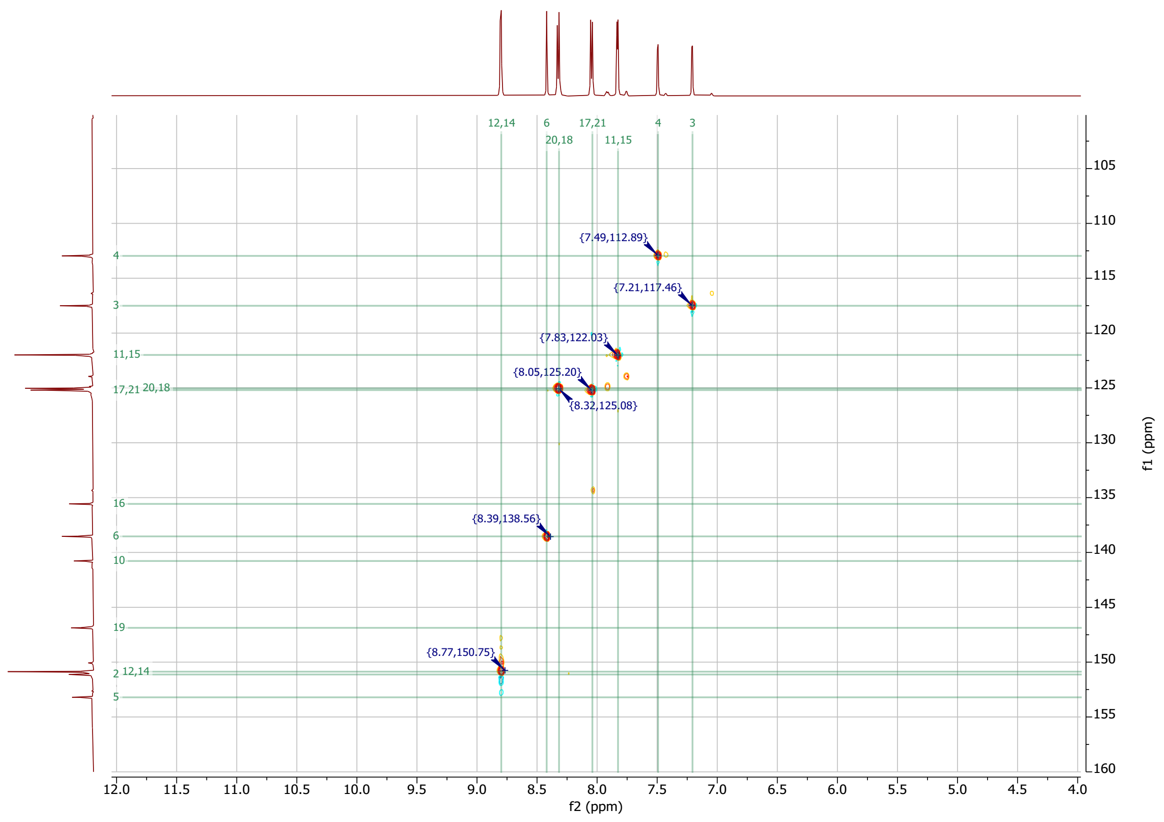

Figure S33: HSQS NMR (DMSO-d6, 298 K) of compound **8**

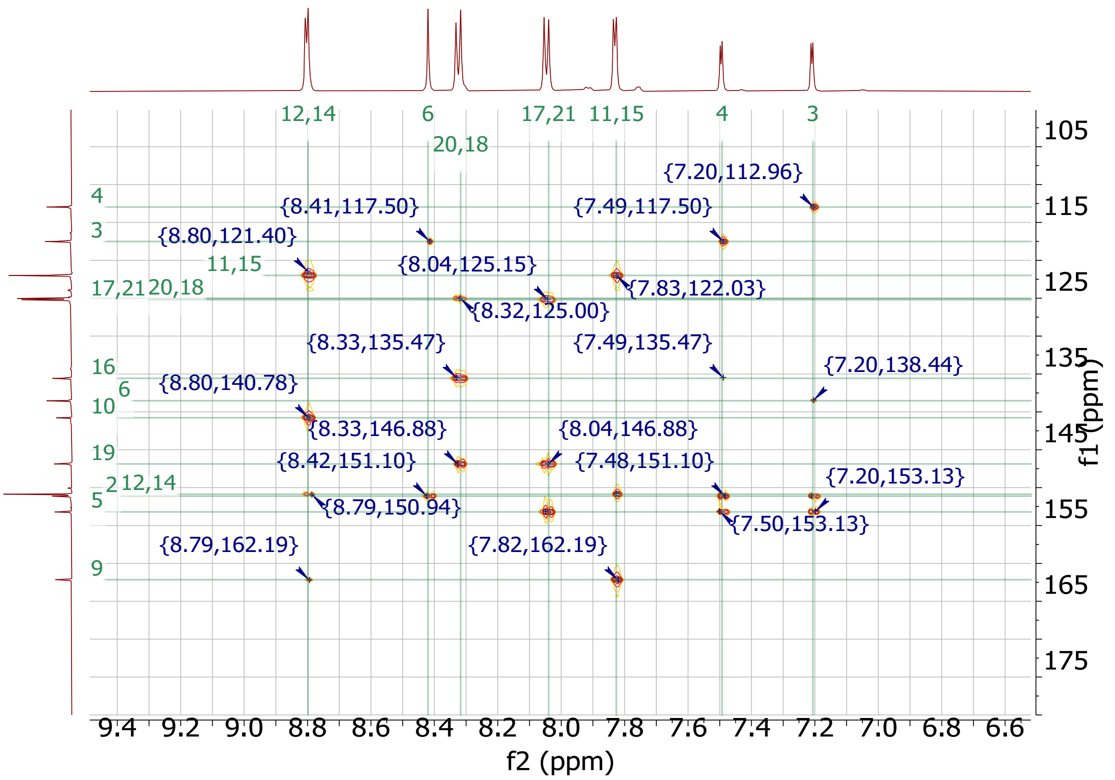

Figure S34: HMBC NMR (DMSO-d6, 298 K) of compound **8**

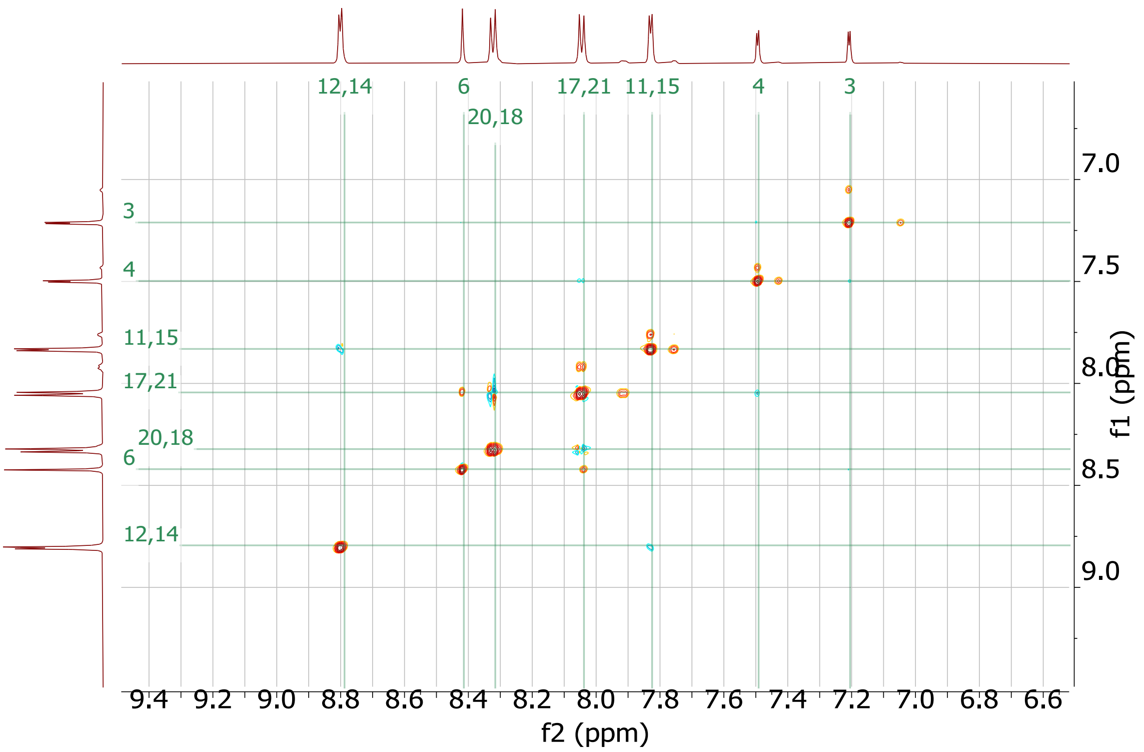

Figure S35: ROESY NMR (DMSO-d6, 298 K) of compound **8**

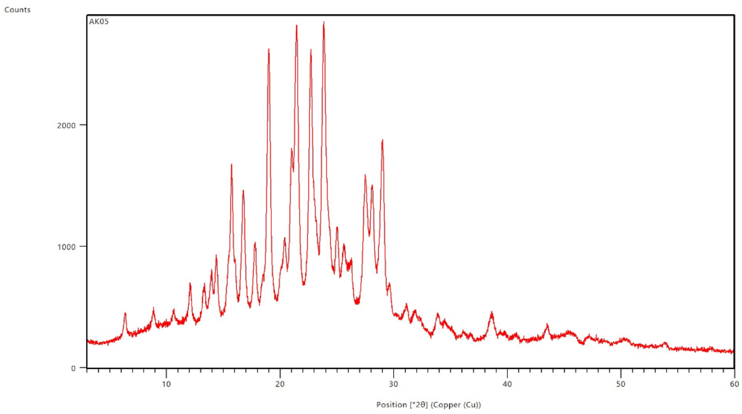

Figure S36: Powder XRD of compound **8**

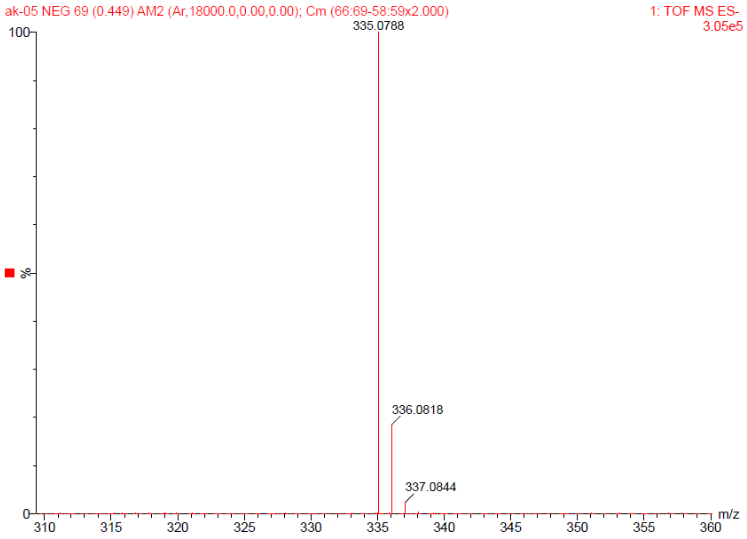

Figure S37: HRMS of compound **8**

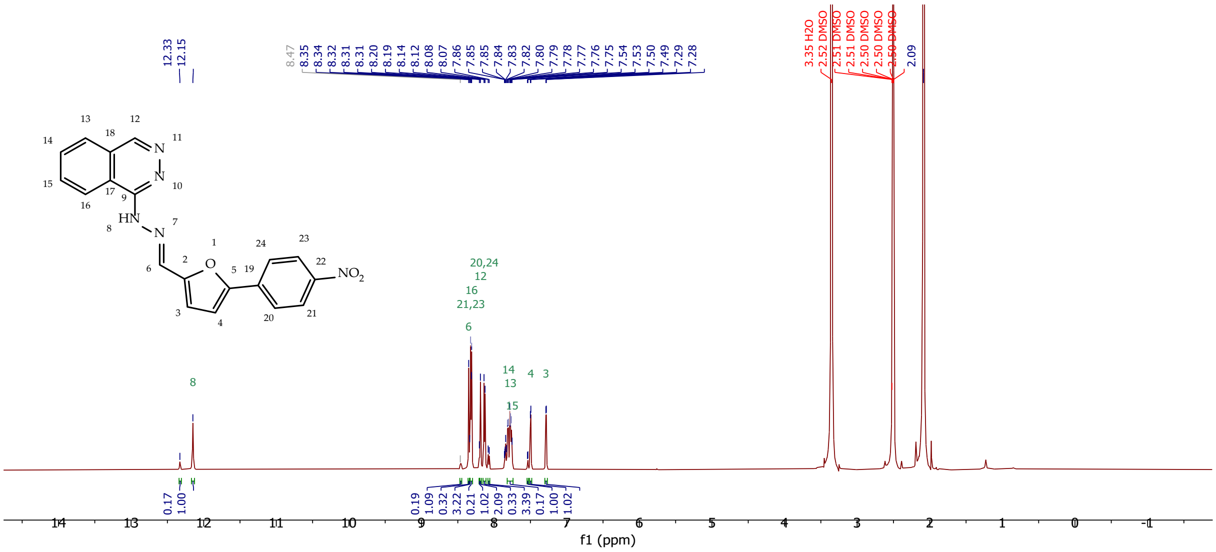

Figure S38: ^{1}H NMR (600 MHz, DMSO-d6, 298 K) of compound **9**

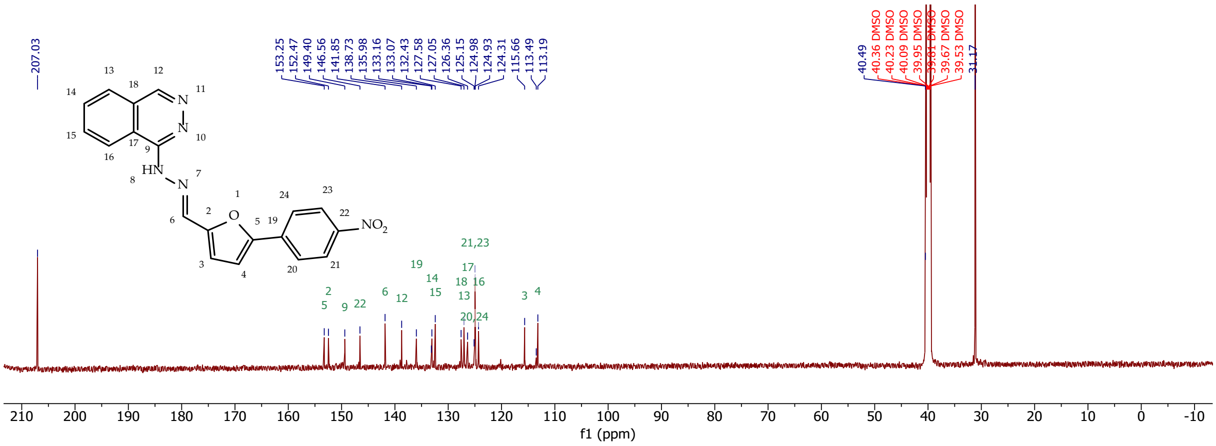

Figure S39: ^{13}C NMR (150 MHz, DMSO-d6, 298 K) of compound **9**

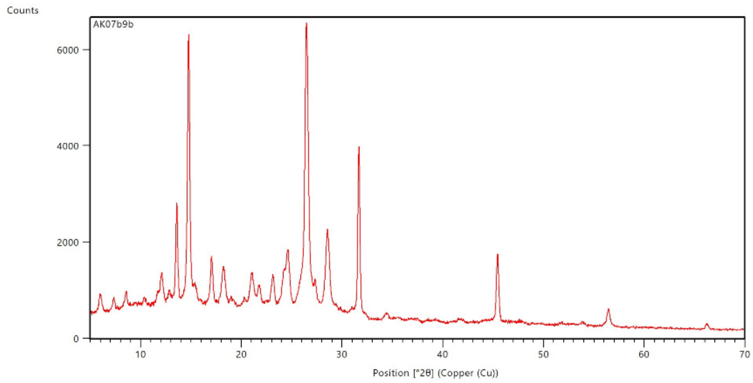

Figure S40: Powder XRD of compound **9**

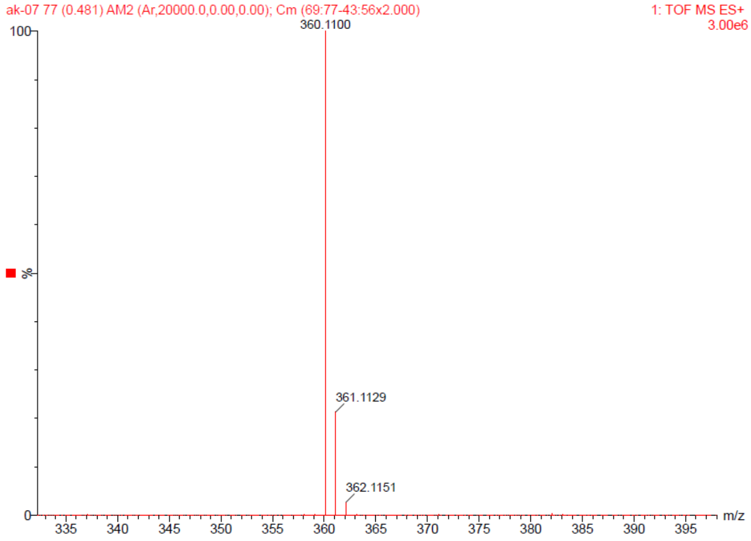

Figure S41: HRMS of compound **9**

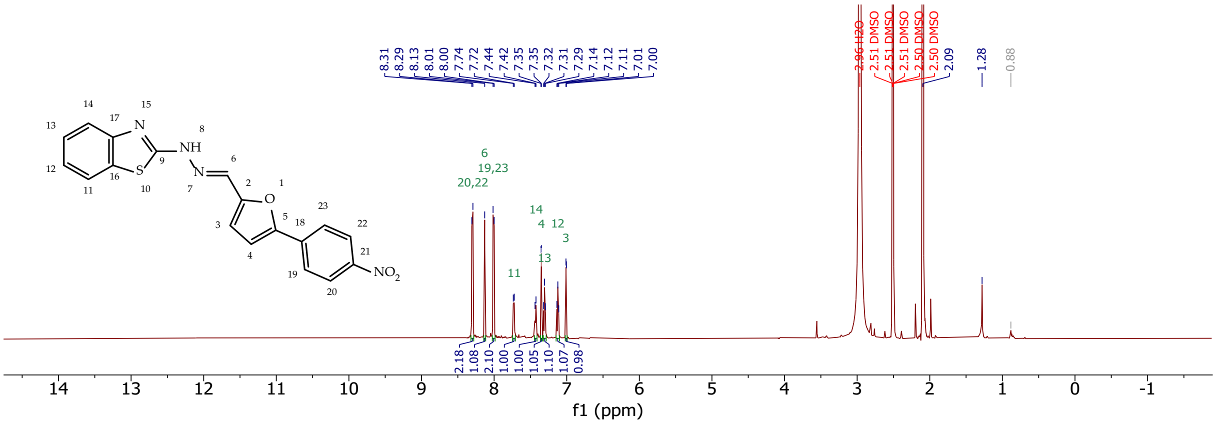

Figure S42: ^{1}H NMR (600 MHz, DMSO-d6, 383 K) of compound **10**

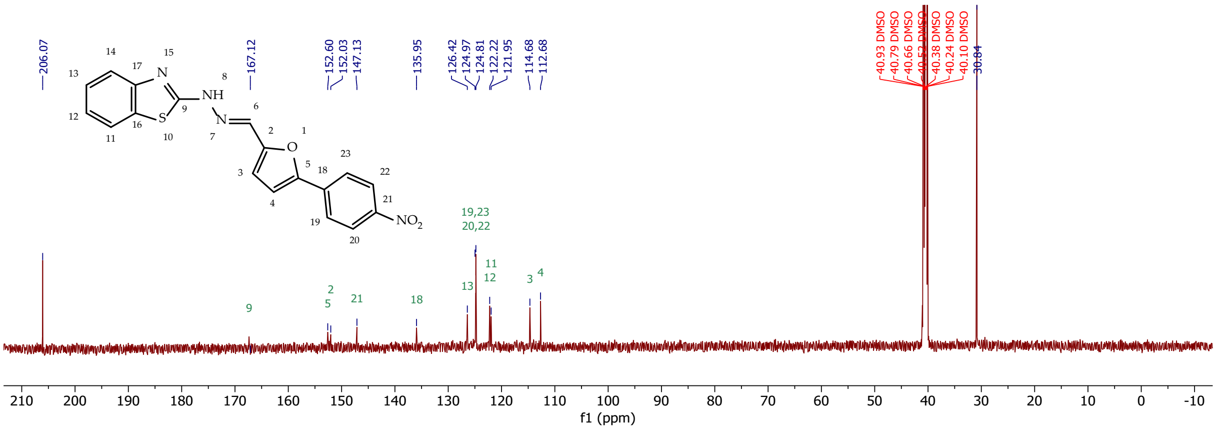

Figure S43: ^{13}C NMR (150 MHz, DMSO-d6, 383 K) of compound **10**

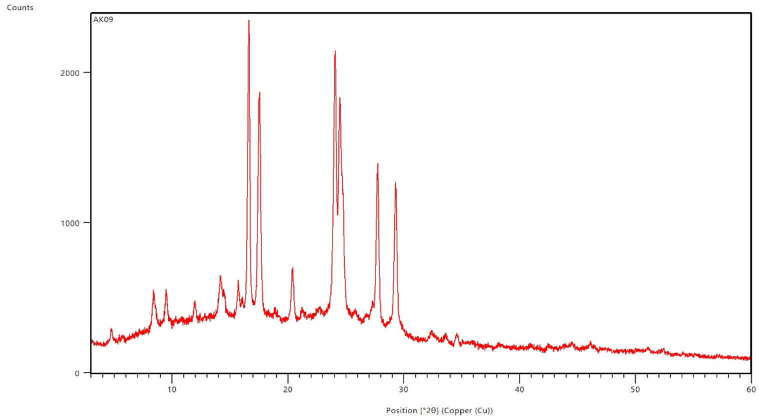

Figure S44: Powder XRD of compound **10**

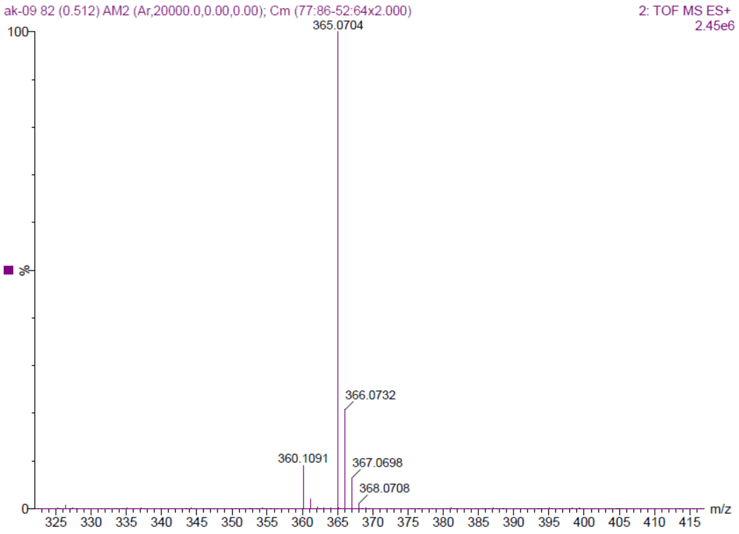

Figure S45: HRMS of compound **10**

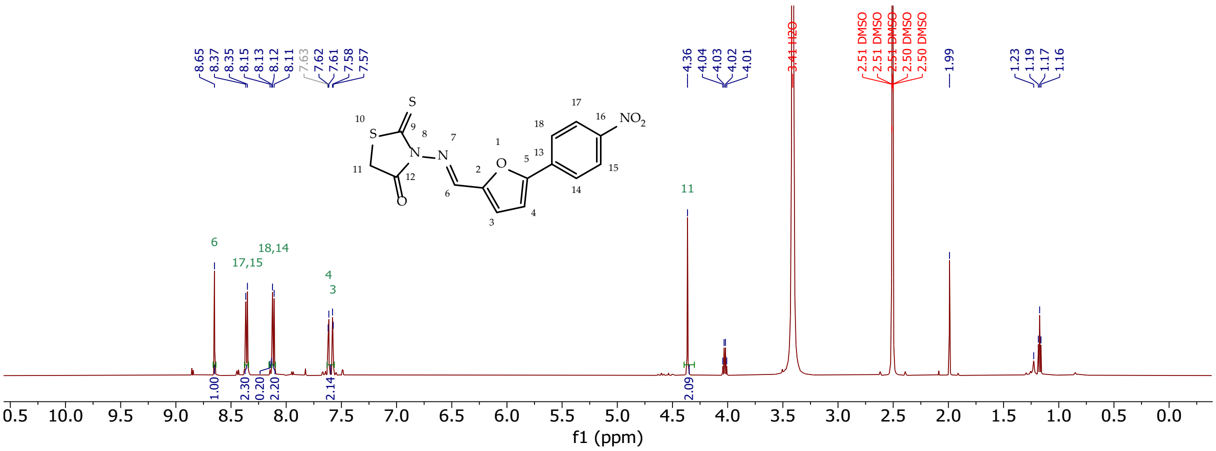

Figure S46: ^{1}H NMR (600 MHz, DMSO-d6, 298 K) of compound **11**

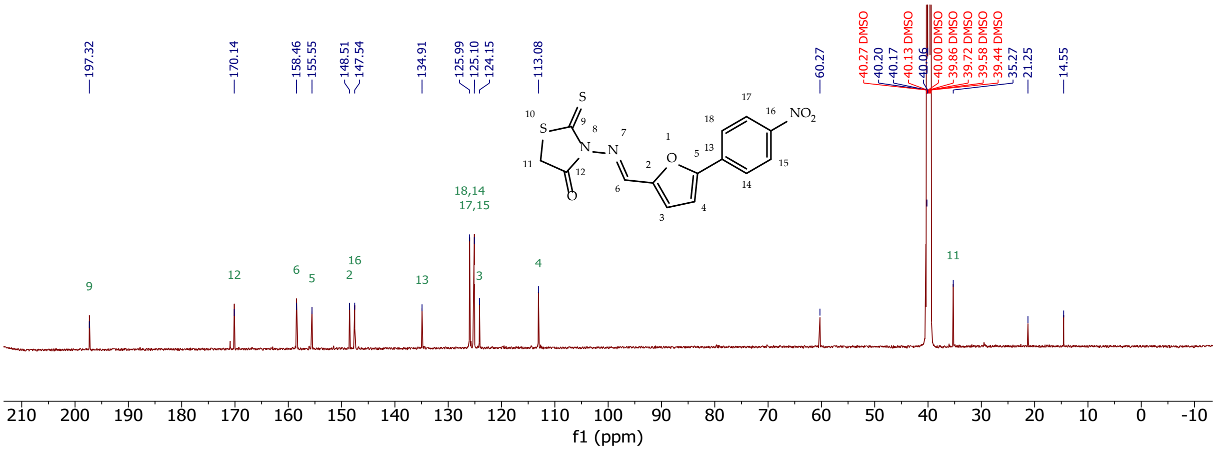

Figure S47: ^{13}C NMR (150 MHz, DMSO-d6, 298 K) of compound **11**

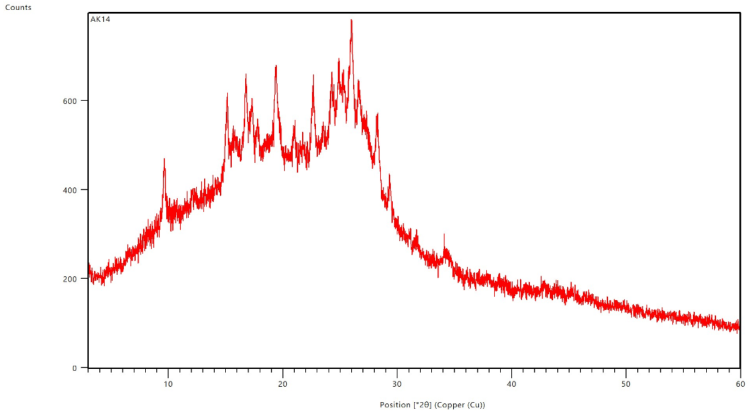

Figure S48: Powder XRD of compound **11**

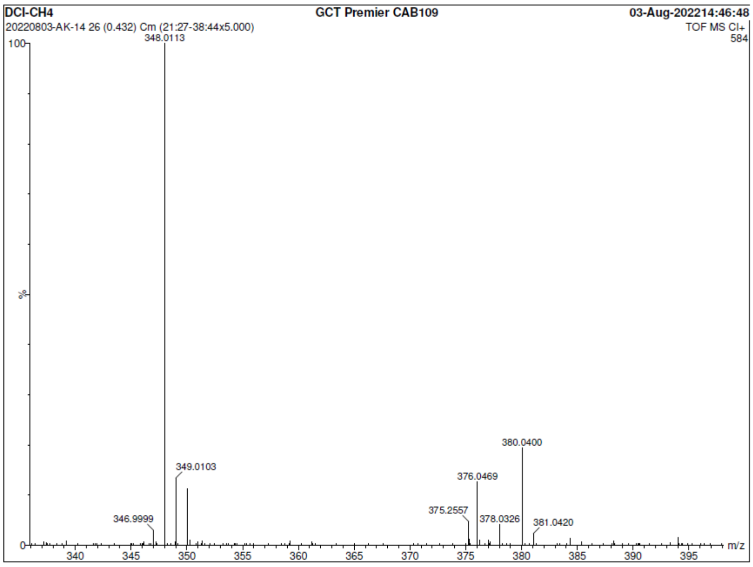

Figure S49: HRMS of compound **11**

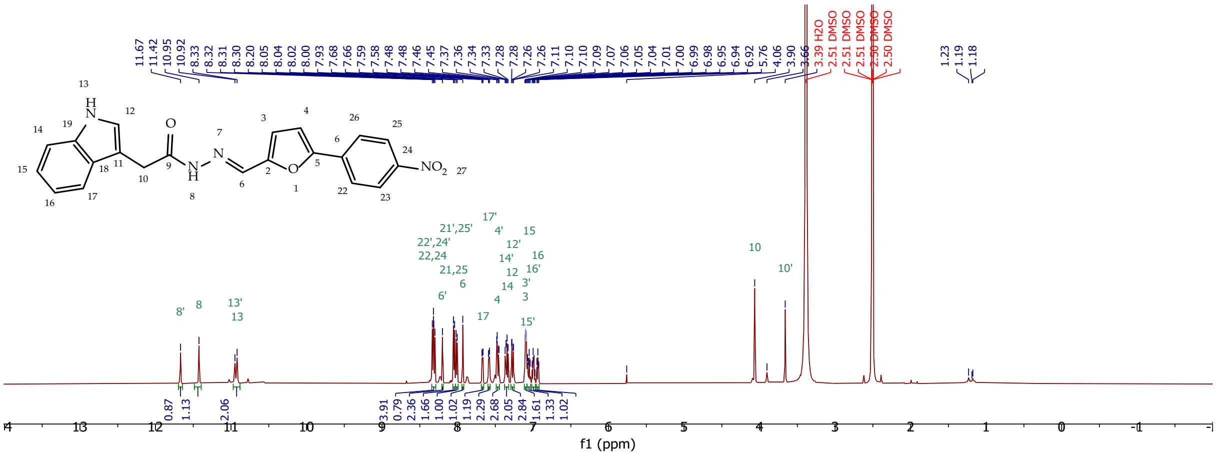

Figure S50: ^{1}H NMR (600 MHz, DMSO-d6, 298 K) of compound **12**

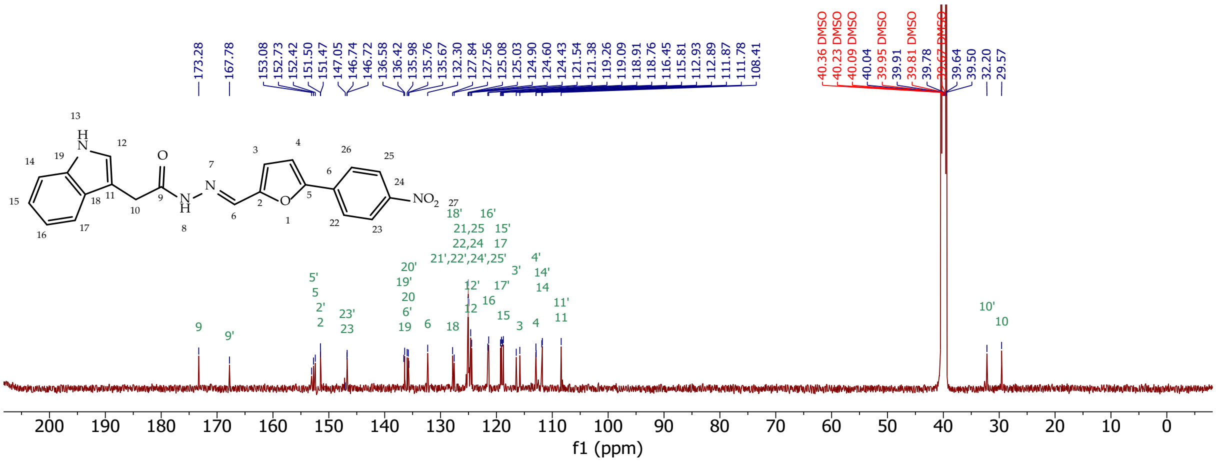

Figure S51: ^{13}C NMR (150 MHz, DMSO-d6, 298 K) of compound **12**

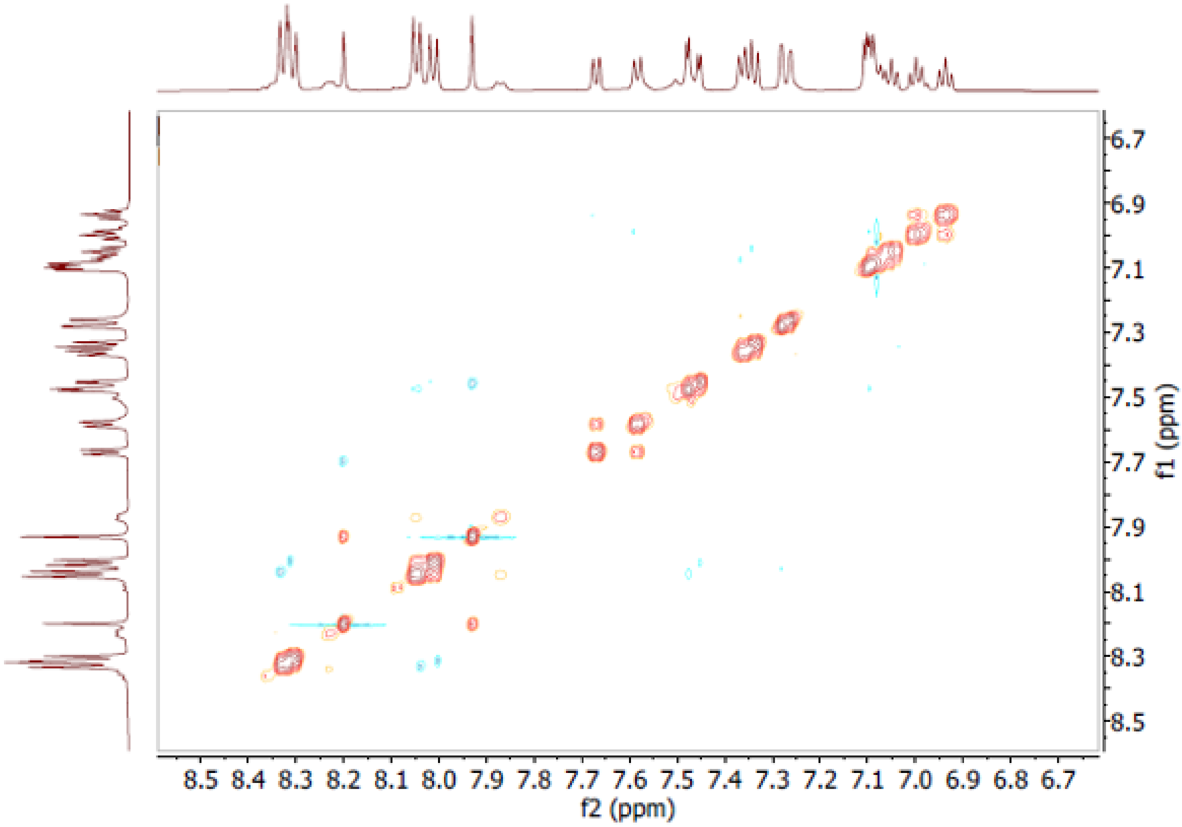

Figure S52: ROESY NMR (DMSO-d6, 298 K) of compound **12**

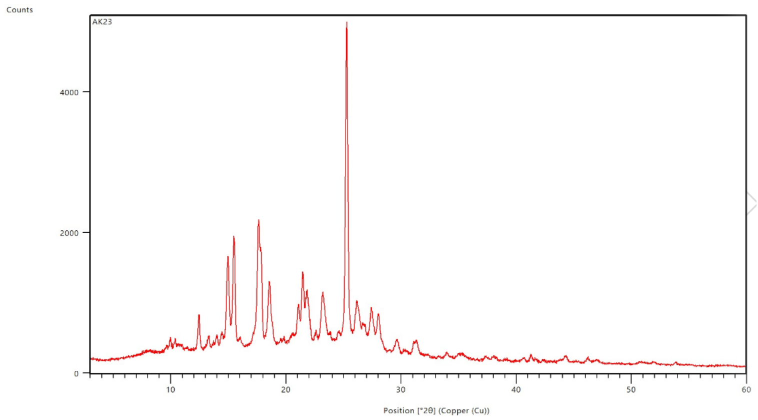

Figure S53: Powder XRD of compound **12**

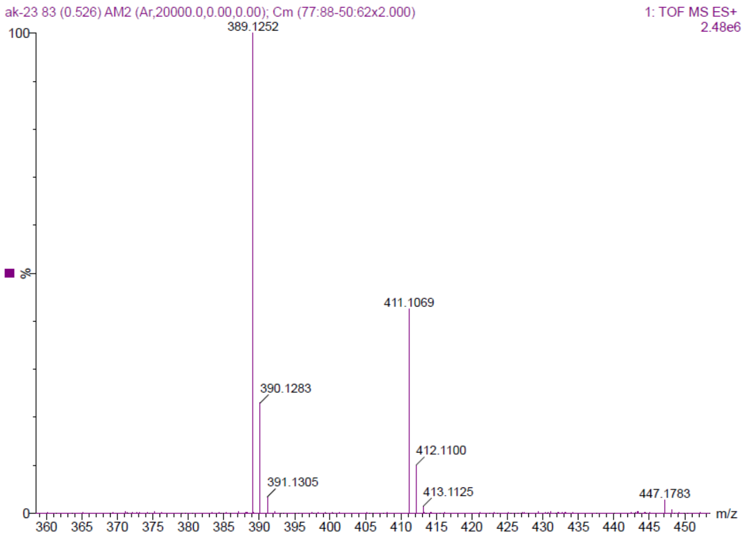

Figure S54: HRMS of compound **12**

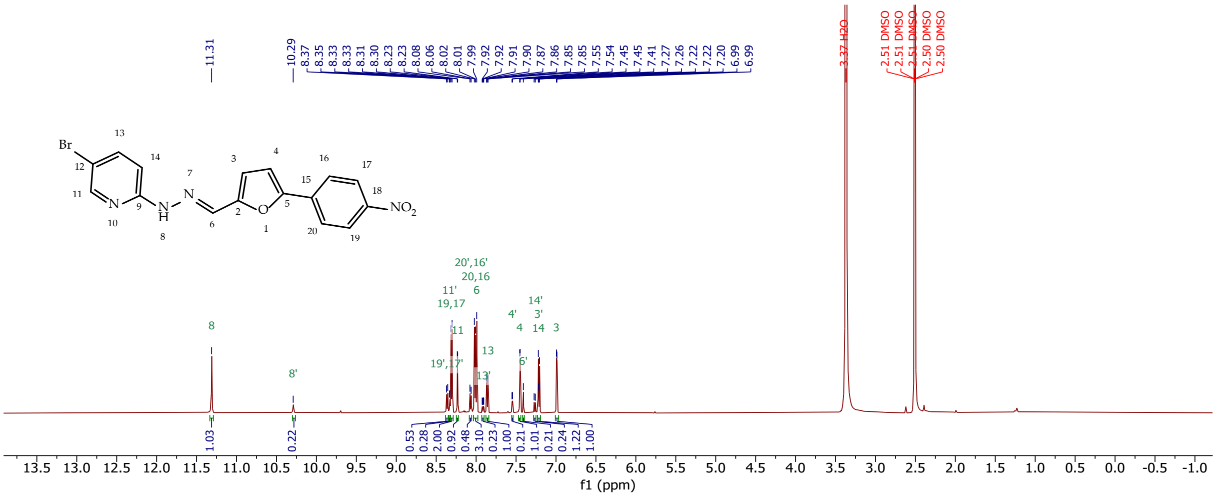

Figure S55: ^{1}H NMR (600 MHz, DMSO-d6, 298 K) of compound **13**

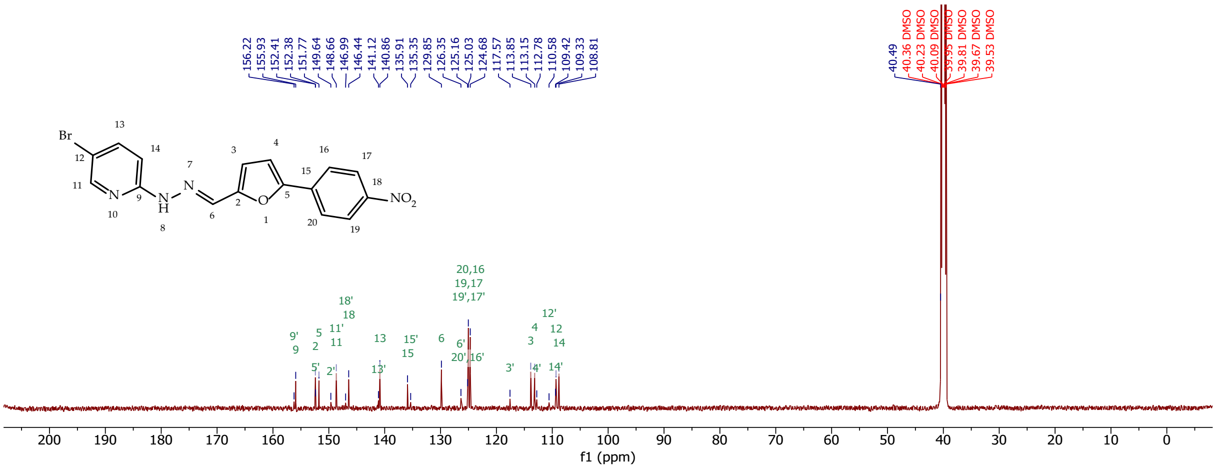

Figure S56: ^{13}C NMR (150 MHz, DMSO-d6, 298 K) of compound **13**

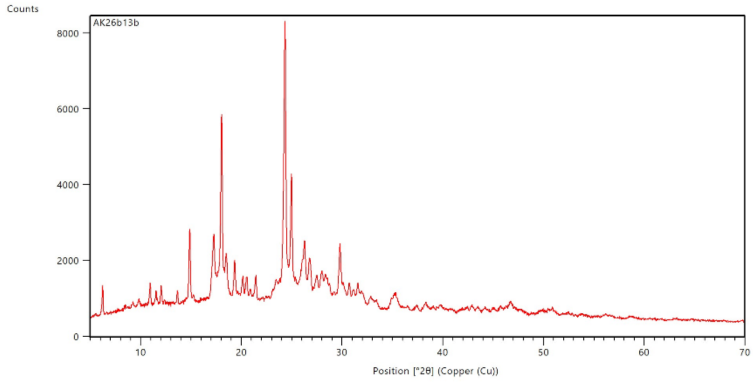

Figure S57: Powder XRD of compound **13**

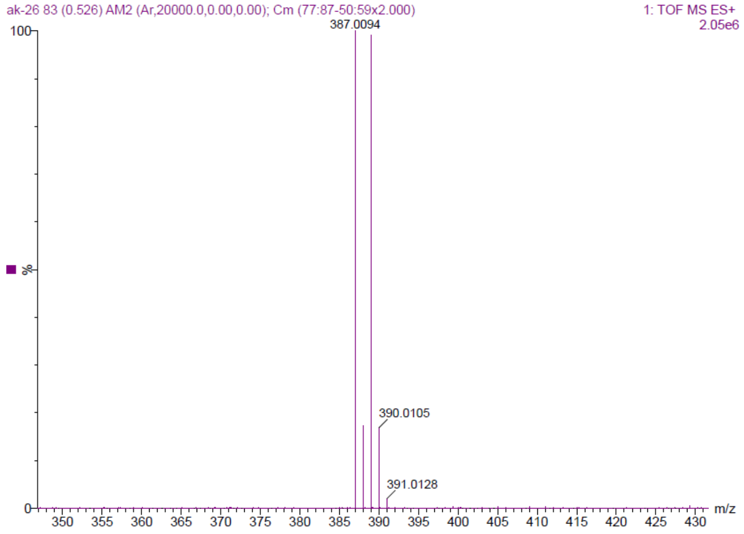

Figure S58: HRMS of compound **13**

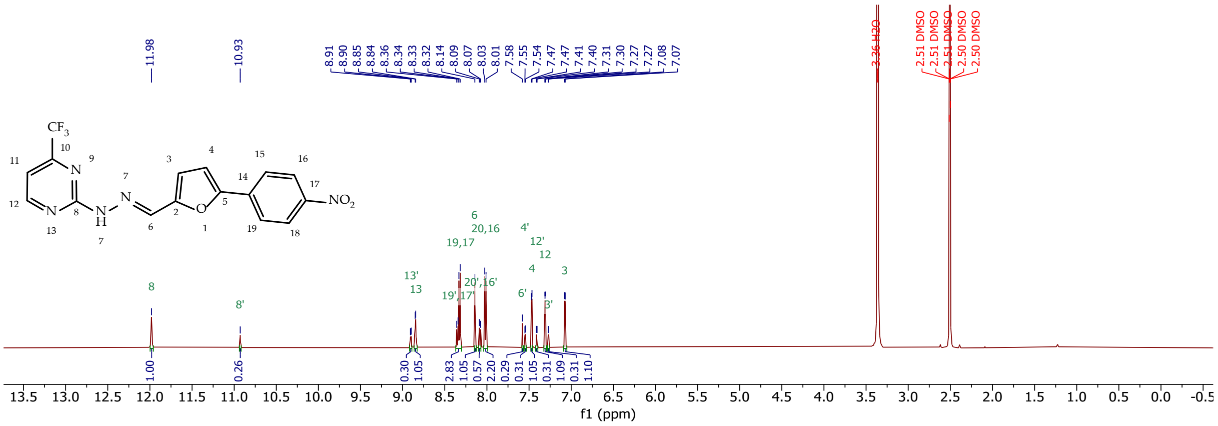

Figure S59: ^{1}H NMR (600 MHz, DMSO-d6, 298 K) of compound **14**

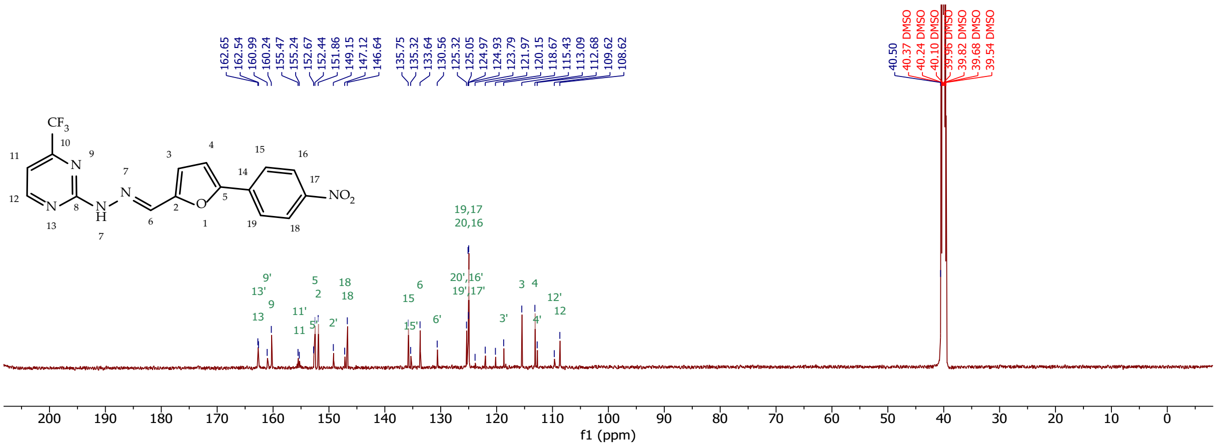

Figure S60: ^{13}C NMR (150 MHz, DMSO-d6, 298 K) of compound **14**

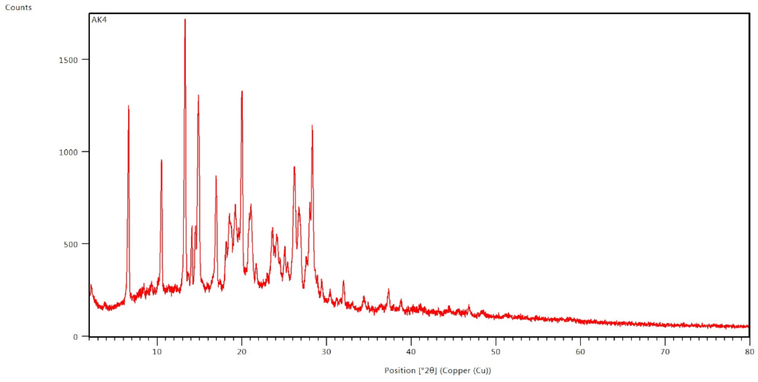

Figure S61: Powder XRD of compound **14**

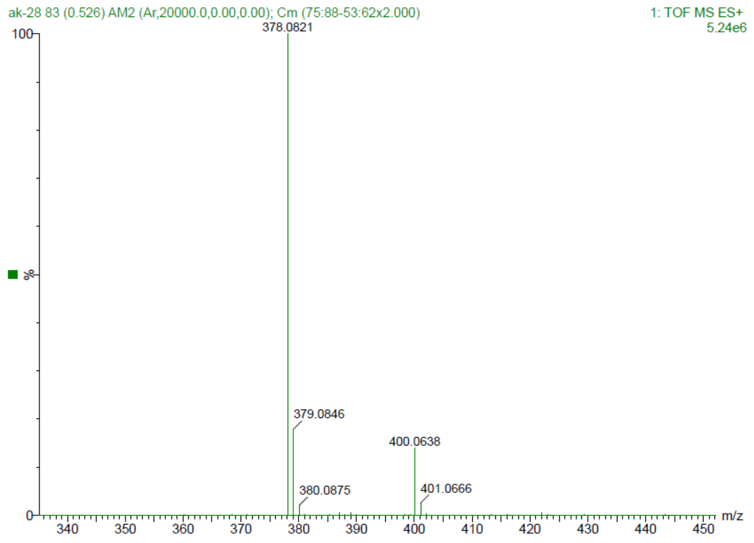

Figure S62: HRMS of compound **14**

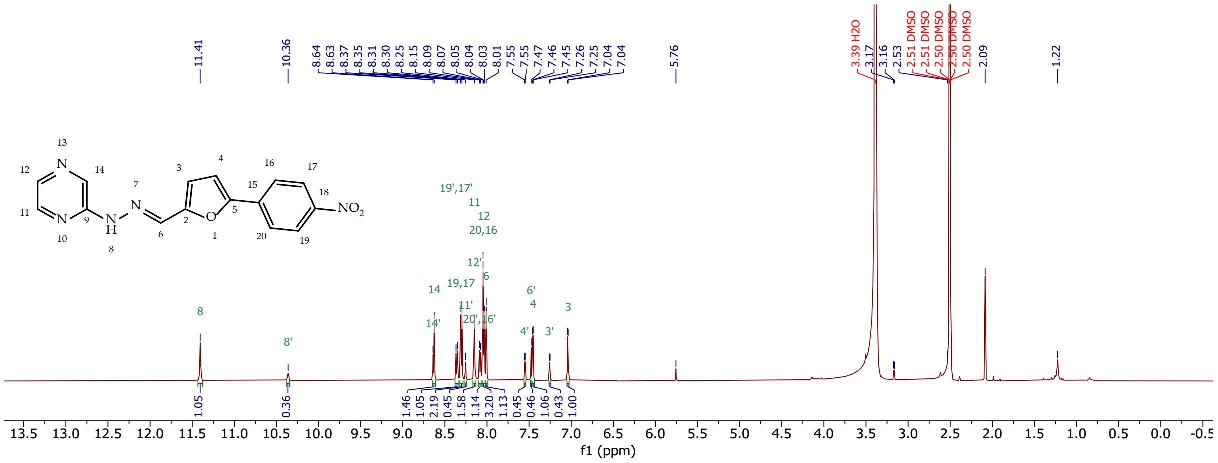

Figure S63: ^{1}H NMR (600 MHz, DMSO-d6, 298 K) of compound **15**

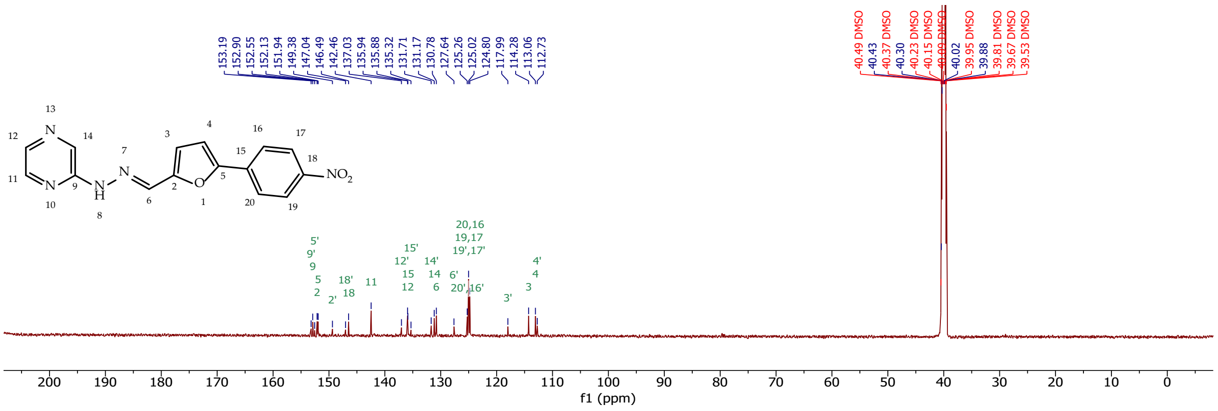

Figure S64: ^{13}C NMR (150 MHz, DMSO-d6, 298 K) of compound **15**

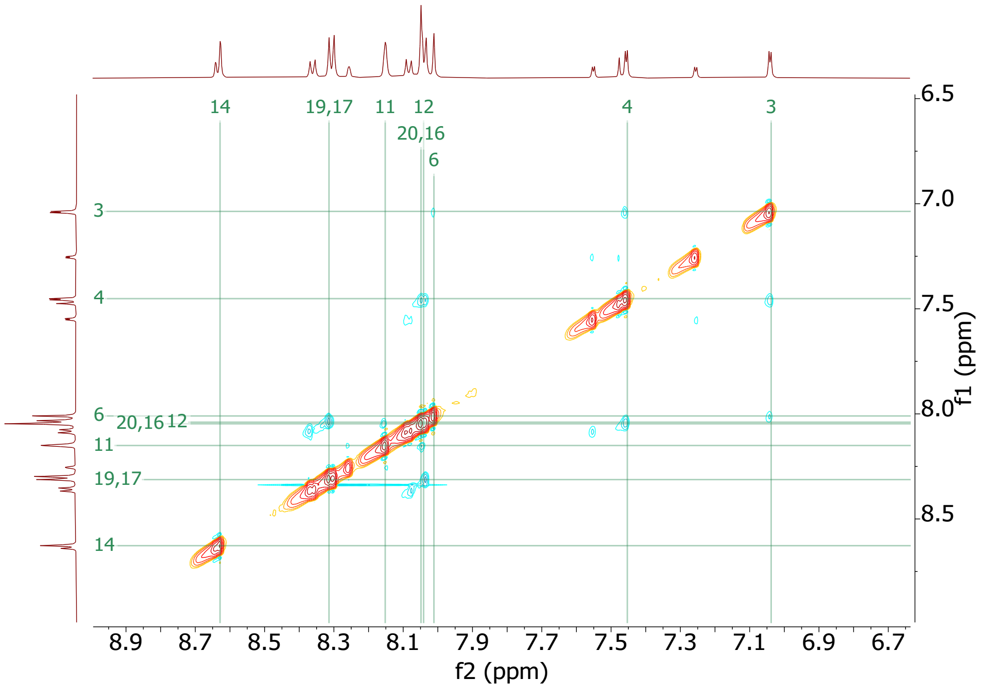

Figure S65a: ROESY NMR (DMSO-d6, 298 K) of compound **15**

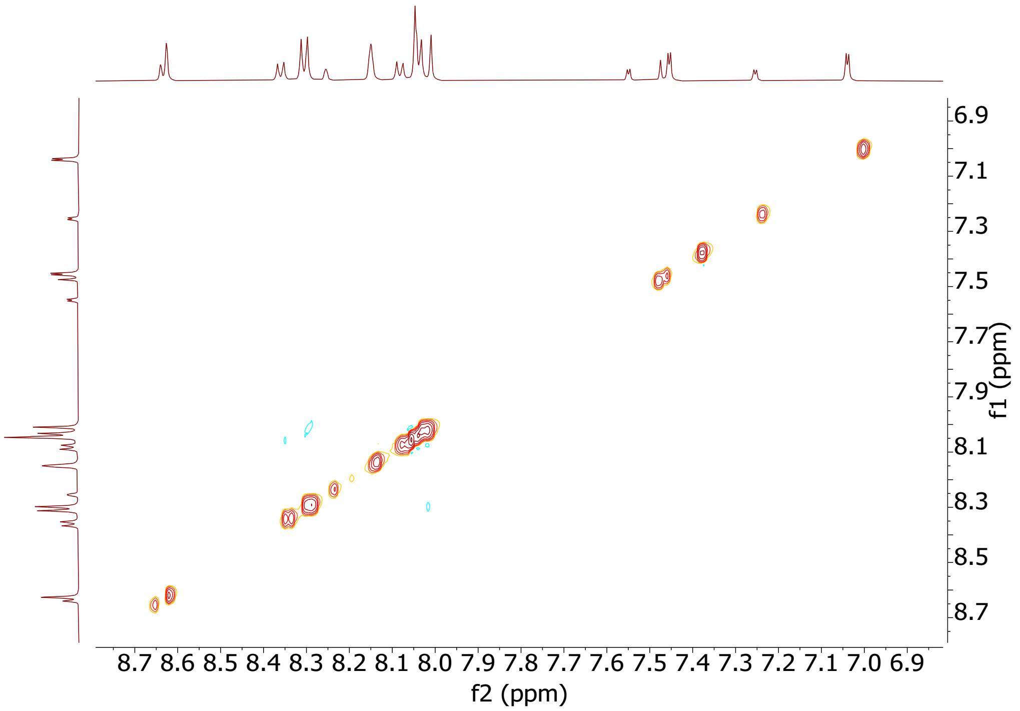

Figure S65b: ROESY NMR (DMSO-d6, 348 K) of compound **15**

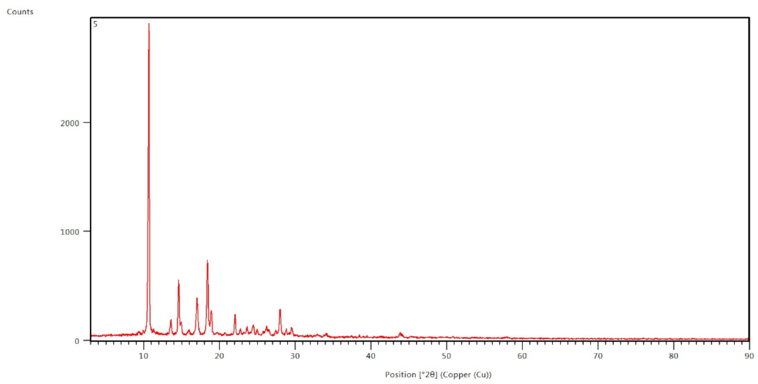

Figure S66: Powder XRD of compound **15**

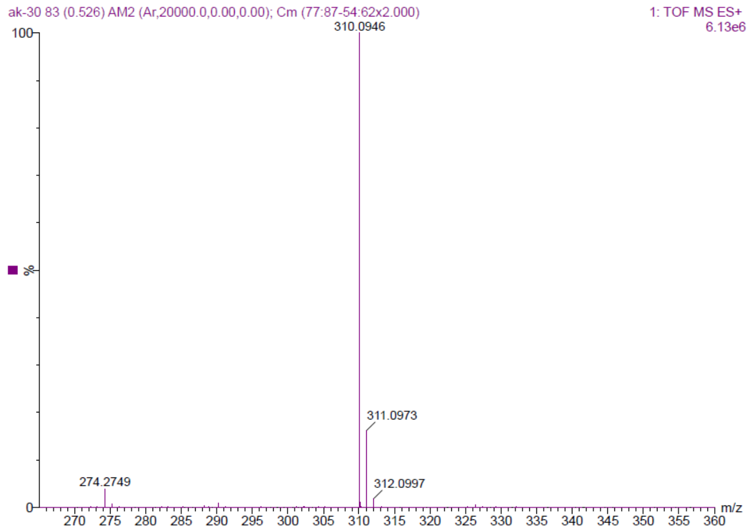

Figure S67: HRMS of compound **15**

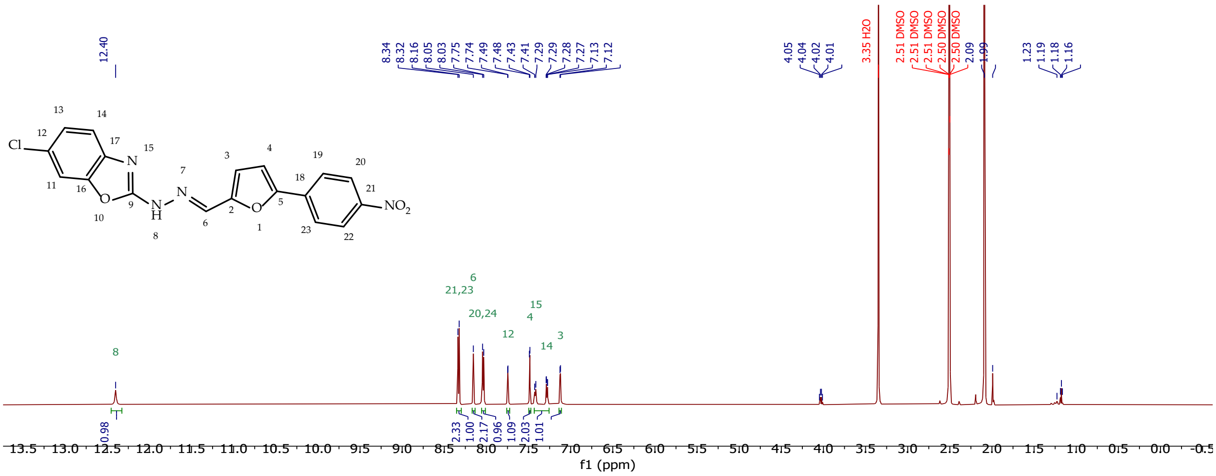

Figure S68: ^{1}H NMR (600 MHz, DMSO-d6, 298 K) of compound **16**

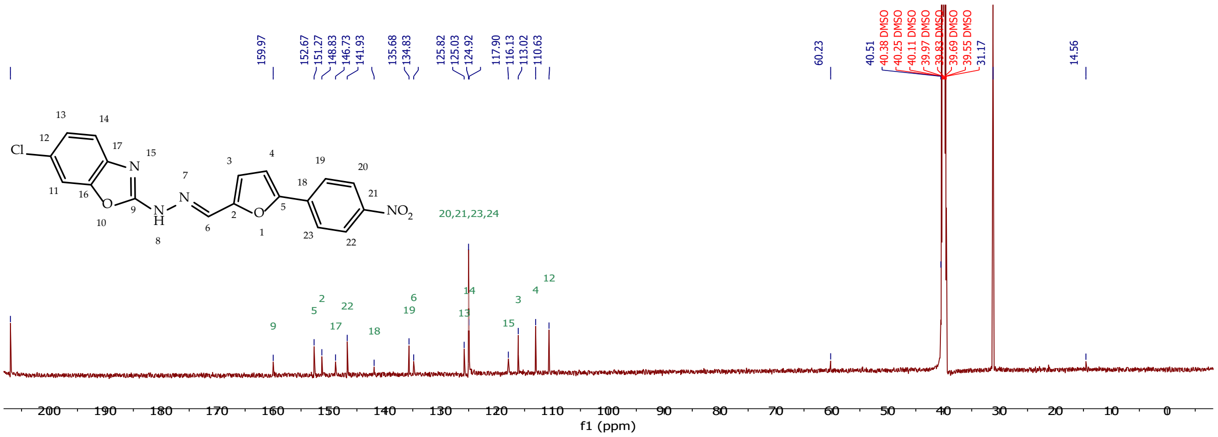

Figure S69: ^{13}C NMR (150 MHz, DMSO-d6, 298 K) of compound **16**

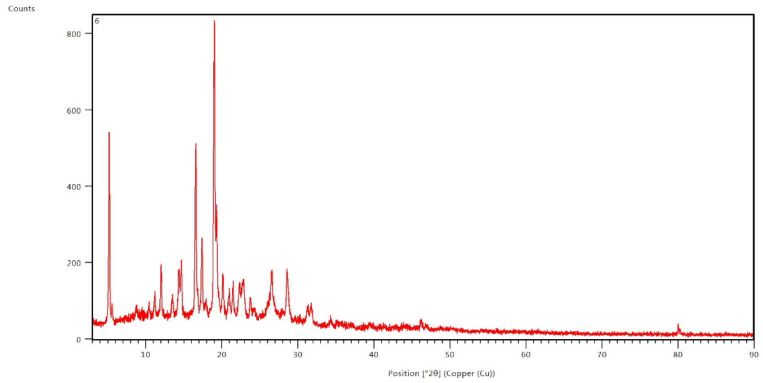

Figure S70: Powder XRD of compound **16**

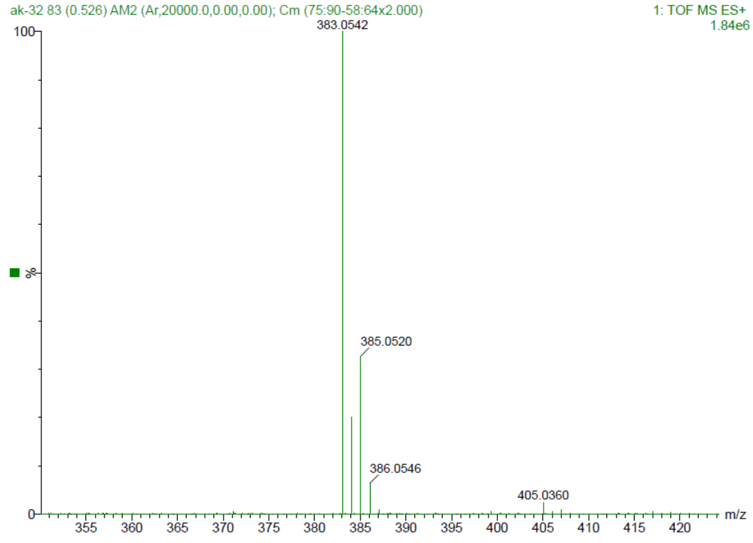

Figure S71: HRMS of compound **16**

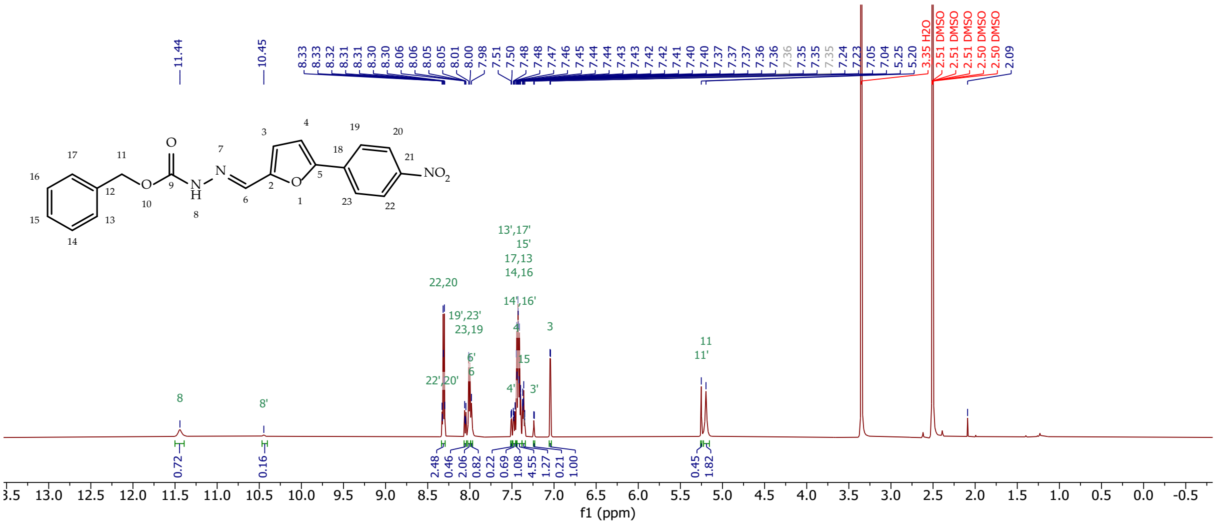

Figure S72: ^{1}H NMR (600 MHz, DMSO-d6, 298 K) of compound **17**

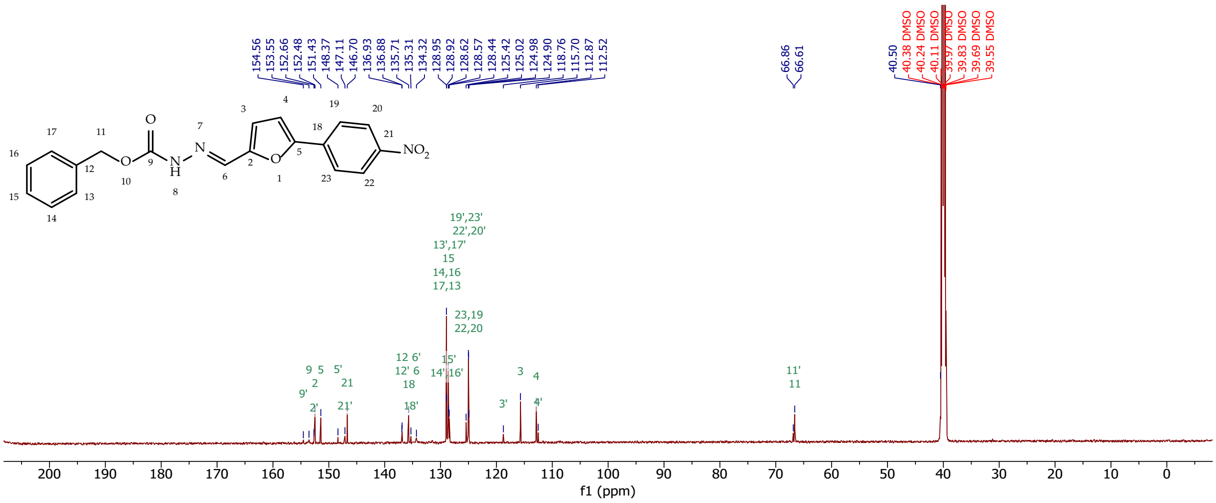

Figure S73: ^{13}C NMR (150 MHz, DMSO-d6, 298 K) of compound **17**

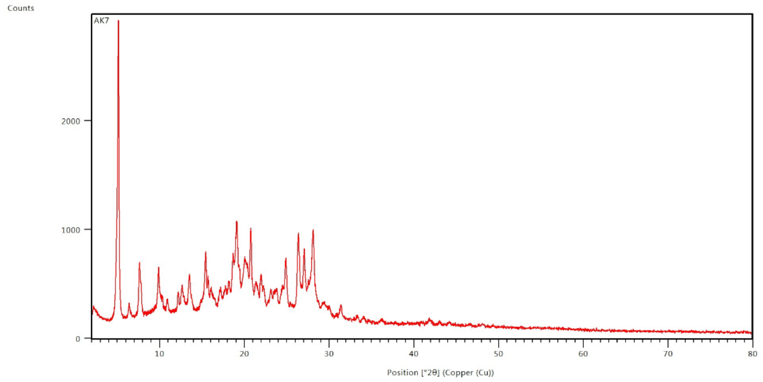

Figure S74: Powder XRD of compound **17**

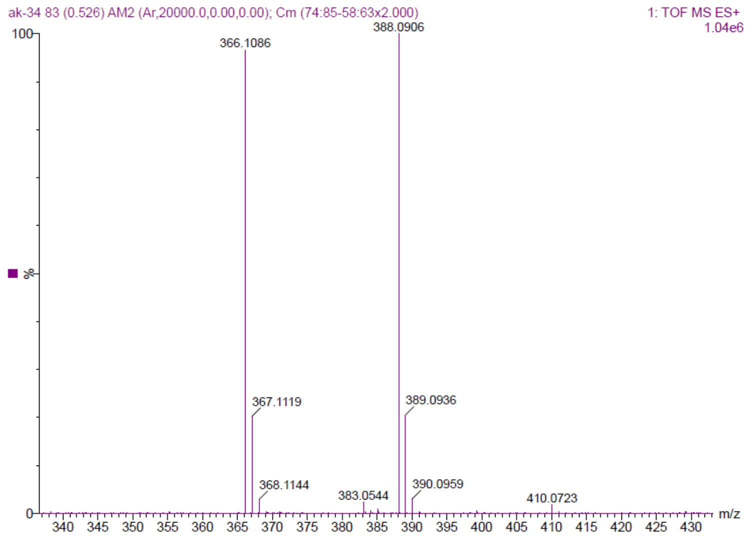

Figure S75: HRMS of compound **17**

Table S1a. ^{1}H NMR Spectroscopic Data of **1**–**7** in DMSO-d6 (δ in ppm, J in Hz)

No.	1	1′	2	3	4	5	6	6′	7	7′
CH_3	3.84, s	3.73, s								
2	7.33, d (1.9)	7.39, d (1.9)	7.30, s							
3				7.21, d (4.0)	7.07, d (4)	7.06, d (3.9)	7.81, d (3.9)	7.79, d (3.9)	7.12, t (4.3)	7.12, t (4.3)
4				7.79, d (4.0)	7.84 – 7.75, m	7.65, d (3.9)	7.41, d (4.0)	7.38, d (4.0)	7.76, d (4.0)	7.75, d (4.0)
5	7.12, dd (8.2) (1.9)	7.07, d (1.9)	6.84, m							
6	6.86, d (8.1)	6.78, d (8.1)	7.06, d (8.2)	7.18 – 7.15, m	7.10, s	6.95, d (16.0)	7.51, d (16.0)	7.44, d (15.5)	7.02 – 6.96, m	7.11 – 7.03, m
7	8.35, s	7.98, s	8.31, s	7.18 – 7.15, m	7.26, dd (16.0) (9.7)	7.12 – 7.08, m	7.26, dd (16.0) (9.6)	6.88, dd (15.5) (9.7)	7.11 – 7.03, m	7.11 – 7.03, m
8	11.88, s	9.77, s		8.27, dd (6.9) (1.9)	8.28, d (9.7)	8.00, d (9.3)	8.59, d (9.6)	8.63, d (9.7)	7.83, d (9.3)	8.03 – 8.00, m
9			11.46, s							
10				12.20, s	12.21, s				11.70, s	11.42, s
12	7.84 – 7.78, m	7.68, m	7.79, d (8.3)						3.98, s	3.63, s
13	8.81 – 8.75, m	8.73, m	6.84, m	7.85 – 7.80, m		7.46, d (8.0)	4.35, s	4.23, s		
14				8.81 – 8.77, m	8.20, s	7.31, t (7.6)			7.22, d (2.4)	7.24, d (2.4)
15	8.81 – 8.75, m	8.73, m	6.84, m		7.84 – 7.75, m	7.13, t (7.6)			10.93, s	10.89, s
16	7.84 – 7.78, m	7.68, m		8.81 – 8.77, m	7.84 – 7.75, m	7.74, d (7.8)			7.35, t (8.3)	7.35, t (8.3)
17				7.85 – 7.80, m	7.84 – 7.75, m				7.11 – 7.03, m	7.11 – 7.03, m
18					8.31, d (7.9)				7.02 – 6.96, m	7.02 – 6.96, m
19			7.79, d (8.3)						7.54, d (7.9)	7.56, d (7.9)

Table S1b. ^{1}H NMR Spectroscopic Data of **8**–**17** in DMSO-d6 (δ in ppm, J in Hz)

No.	8	9	10	11	12	12′	13	13′	14	14′	15	15′	16	17	17′
3	7.21, d (3.6)	7.28, d (3.7)	7.01, d (3.7)	7.58, d (3.8)	7.12 – 7.07, m	7.12 – 7.07, m	6.99, d (3.6)	7.23 – 7.19, m	7.07, d (3.7)	7.27, d (3.7)	7.04, d (3.6)	7.25, d (3.8)	7.12, d (3.7)	7.04, d (3.7)	7.24, d (3.8)

4	7.50, d (3.7)	7.50, d (3.7)	7.35, d (3.7)	7.62, d (3.7)	7.48, d (3.7)	7.45, d (3.6)	7.45, d (3.6)	7.55, d (3.8)	7.47, d (3.7)	7.55, d (3.7)	7.45, d (3.6)	7.55, d (3.7)	7.49, d (3.7)	7.46 – 7.39, m	7.51, d (3.7)
6	8.42, s	8.35, s	8.13, s	8.65, s	7.93, s	8.20, s	7.99, s	7.41, s	8.14, s	7.58, s	8.01, s	7.47, s	8.16, s	7.98, s	7.98, s
8	12.19, s	12.15, s			11.42, s	11.67, s	11.31, s	10.29, s	11.98, s	10.93, s	11.41, s	10.36, s	12.40, s	11.44, s	10.45, s
10					4.06, s	3.66, s									
11	7.83, d (5.8)		7.73, d (7.8)	4.36, s			8.23, d (2.4)	8.33, d (2.4)			8.16 – 8.14, m	8.25, s		5.20, s	5.25, s
12	8.80, d (5.8)	8.19, s	7.12, t (5.1)		7.28, d (2.4)	7.26, d (2.4)			7.31, d (4.9)	7.41, d (4.9)	8.05 – 8.03, m	8.16 – 8.14, m	7.75, d (2.1)		
13		7.74- 7.79, m	7.31, t (7.1)		10.92, s	10.95, s	7.86, dd (9.0) (2.5)	7.91, dd (8.9) (2.5)	8.85, d (4.9)	8.90, d (4.9)				7.46 – 7.39, m	7.49 – 7.46, m
14	8.80, d (5.8)	7.81, d (7.3)	7.43, d (7.9)	8.12, d (8.9)	7.34, d (8.3)	7.36, d (7.4)	7.23 – 7.19, m	7.27, d (8.9)			8.63, s	8.64, s	7.28, dd (8.4) (2.0)	7.46 – 7.39, m	7.46 – 7.39, m
15	7.83, d (5.8)	7.79- 7.74, m		8.36, d (8.9)	7.05, t (7.5)	7.12 – 7.07, m							7.42, d (8.3)	7.38 – 7.34, m	7.38 – 7.34, m
16		8.33 – 8.30, m			6.94, t (7.5)	7.00, t (7.3)	8.01, d (9.0)	8.07, d (8.7)	8.02, d (8.9)	8.08, d (8.9)	8.05 – 8.03, m	8.08, d (8.9)		7.46 – 7.39, m	7.46 – 7.39, m
17	8.05, d (8.8)			8.36, d (8.9)	7.67, d (7.9)	7.58, d (8.0)	8.31, d (8.9)	8.36, d (8.9)	8.32, d (9.0)	8.35, d (8.9)	8.31, d (8.8)	8.36, d (9.0)		7.46 – 7.39, m	7.49 – 7.46, m
18	8.32, d (8.9)			8.12, d (8.9)											
19			8.01, d (9.0)				8.31, d (8.9)	8.36, d (8.9)	8.32, d (9.0)	8.35, d (8.9)	8.31, d (8.8)	8.36, d (9.0)		8.01, d (8.5)	8.08 – 8.03, m
20	8.32, d (8.9)	8.13, d (8.5)	8.30, d (8.9)				8.01, d (9.0)	8.07, d (8.7)	8.02, d (8.9)	8.08, d (8.9)	8.05 – 8.03, m	8.08, d (8.9)	8.04, d (8.8)	8.35 – 8.28, m	8.35 – 8.28, m
21	8.05, d (8.8)	8.33 – 8.30, m			8.05, d (8.9)	8.01, d (8.8)							8.33, d, (8.9)		
22			8.30, d (8.9)		8.33, d (8.9)	8.31, d (8.9)								8.35 – 8.28, m	8.35 – 8.28, m
23		8.33 – 8.30, m	8.01, d (9.0)										8.33, d (8.9)	8.01, d (8.5)	8.08 – 8.03, m
24		8.13, d (8.5)			8.33, d (8.9)	8.31, d (8.9)							8.04, d (8.8)		
25					8.05, d (8.9)	8.01, d (8.8)									

Table S2a. ^{13}C NMR Spectroscopic Data of **1**–**7** in DMSO-d6 (δ in ppm)

No.	1	2	3	4	5	6	6′	7	7′
CH_3	56.04, CH_3	55.99, CH_3							
1	125.86 (1C	126.36, C							
2	109.56, CH	109.26, CH	155.21, C	155.70, C	155.73, C	153.80, C	153.42, C	155.33, C	155.40, C
3	149.77, C	148.49, C	114.21, CH	114.03, CH	113.24, CH	115.38, CH	115.25, CH	113.85, CH	113.85, CH
4	148.54, C	149.30, C	115.84, CH	116.10, CH	115.48, CH	116.51, CH	117.93, CH	115.88, CH	115.95, CH
5	115.94, CH	115.88, CH	151.91, C	151.78, C	151.86, C	152.47, C	152.77, C	151.77, C	151.77, C
6	122.90, CH	122.43, CH	125.20, CH	122.82, CH	122.17, CH	132.06, CH	132.45, CH	123.35, CH	123.97, CH
7	150.03, CH	147.86, CH	130.75, CH	132.21, CH	130.92, CH	127.87, CH	122.18, CH	130.84, CH	131.03, CH
8			149.55, C	153.35, CH	145.46, CH	169.97, CH	168.99, CH	143.60, CH	146.81, CH
10	161.77, C=O	162.99, C=O							
11	141.16, C	124.51, C	162.17, C=O	149.56, C	167.22, C	197.45, C=S	196.90, C=S	173.32, C=O	167.91, C=O
12	121.95, CH	130.01, CH	140.62, C					29.32, CH_2	31.16, CH_2
13	150.76, CH	115.45, CH	122.02, CH		118.04, CH	35.23, CH_2	36.01, CH_2	108.24, C	108.32, C
14		161.06, C	150.84, CH	139.07, CH	126.45, CH	170.03, C=O	169.62, C=O	124.56, CH	124.46, CH

15	150.76, CH	115.45, CH		127.10, CH	122.41, CH				
16	121.95, CH		150.84, CH	133.24, CH	121.95, CH			111.79, CH	111.87, CH
17			122.02, CH	132.52, CH				121.41, CH	123.35, CH
18				124.40, CH	122.26, C[1]			118.93, CH	118.83, CH
19		130.01, CH		127.09, C	129.55, C[1]			119.16, CH	119.05, CH
20				127.68, CH				127.81, C	127.54, C
21								136.43, C	136.53, C

[1] Rotation of the benzothiazole frame makes for broad and hard to identify peaks.

Table S2b. ^{13}C NMR Spectroscopic Data of **8–17** in DMSO-d6 (δ in ppm)

No.	**8**	**9**	**10**	**11**	**12**	**12′**	**13**	**13′**	**14**	**14′**	**15**	**15′**	**16**	**17**	**17′**
2	151.13, C	152.47, C	152.03, C	148.51, C	151.50, C	151.47, C	152.41, C	149.64, C	151.86, C	149.15, C	152.13, C	149.38, C	151.27, C	152.48, C	152.66, C
3	117.51, CH	115.66, CH	114.68, CH	124.15, CH	115.81, CH	116.45, CH	113.85, CH	117.57, CH	115.43, CH	118.67, CH	114.28, CH	117.99, CH	116.13, CH	115.70, CH	118.76, CH
4	112.96, CH	113.19, CH	112.68, CH	113.08, CH	112.93, CH	112.89, CH	113.15, CH	112.78, CH	113.09, CH	112.68, CH	113.06, CH	112.73, CH	113.02, CH	112.87, CH	112.52, CH
5	153.21, C	153.25, C	152.60, C	155.55, C	152.42, C	153.08, C	151.77, C	152.38, C	152.44, C	152.67, C	151.94, C	152.55, C	152.67, C	151.43, C	148.37, C
6	138.53, CH	141.85, CH	134.64, C	158.46, CH	132.30, CH	135.98, CH	129.85, CH	126.35, CH	133.64, CH	130.56, CH	130.78, CH	127.64, CH	134.83, CH	134.32, CH	134.32, CH
9	162.15, C=O	149.40, C	167.12, C	197.32, C=S	173.28, C=O	167.78, C=O	155.93, C	156.22, C	160.24, C	160.99, C	152.90, C	153.19, C	159.97, C	153.55, C=O	154.56, C=O
10	140.78, C				29.57, CH_2	32.20, CH_2			155.25, C, q (34.8)						
CF_3									121.06, CF_3, q (271.0)						
11	121.99, CH		121.95, CH	35.27, CH_2	108.43, C	108.41, C	148.66, CH	148.66, CH	108.62, CH	109.62, CH	142.46, CH	142.46, CH		66.61, CH_2	66.86, CH_2
12	150.87, CH	138.73, CH	122.22, CH	170.14, C=O	124.60, CH	124.43, CH	109.33, CH	110.58, CH	162.65, CH	162.54, CH	135.94, CH	137.03, CH	110.63, CH	136.93, C	136.88, C
13		127.05, CH	126.42, CH	134.91, C			140.86, CH	141.12, CH				125.82 (C		128.95, CH	128.44, CH
14	150.87, CH	133.07, CH	117.60, CH	125.99, CH	111.78, CH	111.87, CH	108.81, CH	109.42, CH			131.17, CH	131.71, CH	124.92, CH	128.95, CH	128.92, CH
15	121.99, CH	132.43, CH		125.10, CH	118.76, CH	118.91, CH	135.91, C	135.35, C	135.75, C	135.32, C	135.88, C	135.32, C	117.90, CH	128.62, CH	128.57, CH
16	135.57, C	124.31, CH	129.10, C	147.54, C	121.38, CH	121.54, CH	124.68, CH	125.16, CH	124.93, CH	125.32, CH	124.80, CH	125.26, CH		128.95, CH	128.92, CH
17	125.20, CH	126.36, C	149.20, C	125.10, CH	119.26, CH	119.09, CH	125.03, CH	125.03, CH	125.05, CH	124.97, CH	125.02, CH	125.02, CH	148.83, C	128.95, CH	128.44, CH
18	125.04, CH	127.58, C	135.95, CH	125.99, CH	127.84, C	127.56, C	146.44, C	146.99, C	146.64, C	147.12, C	146.49, C	147.04, C	141.93, C	135.71, C	135.31, C
19	146.87, C	135.98, C	124.97, CH		136.42, C	136.58, C	125.03, CH	125.03, CH	125.05, CH	124.97, CH	125.02, CH	125.02, CH	135.68, C	124.98, CH	125.42, CH
20	125.04, CH	124.98, CH	124.81, CH		135.76, C	135.67, C	124.68, CH	125.16, CH	124.93, CH	125.32, CH	124.80, CH	125.26, CH	125.03, CH	125.02, CH	124.90, CH
21	125.20, CH	124.93, CH	147.13, C		124.90, CH	125.03, CH							125.03, CH	146.70, C	147.11, C
22		146.56, C	124.81, CH		125.08, CH	125.03, CH							146.73, C	125.02, CH	124.90, CH
23		124.93, CH	124.97, CH		146.72, C	146.74, C							125.03, CH	124.98, CH	125.42, CH
24		124.98, CH			125.08, CH	125.03, CH							125.03, CH		
25					124.90, CH	125.03, CH									
